# Autozygosity islands and ROH patterns in Nellore lineages: evidence of selection for functionally important traits

**DOI:** 10.1186/s12864-018-5060-8

**Published:** 2018-09-17

**Authors:** Elisa Peripolli, Julia Metzger, Marcos Vinícius Antunes de Lemos, Nedenia Bonvino Stafuzza, Sabrina Kluska, Bianca Ferreira Olivieri, Fabieli Louise Braga Feitosa, Mariana Piatto Berton, Fernando Brito Lopes, Danísio Prado Munari, Raysildo Barbosa Lôbo, Cláudio de Ulhoa Magnabosco, Fernando Di Croce, Jason Osterstock, Sue Denise, Angélica Simone Cravo Pereira, Fernando Baldi

**Affiliations:** 1 Departamento de Zootecnia, Faculdade de Ciências Agrárias e Veterinárias, UNESP Univ Estadual Paulista Júlio de Mesquita Filho, Jaboticabal, 14884-900 Brazil; 2 Institute for Animal Breeding and Genetics, University of Veterinary Medicine Hannover, 30559 Hannover, Germany; 3 Departamento de Ciências Exatas, Faculdade de Ciências Agrárias e Veterinárias, UNESP Univ Estadual Paulista Júlio de Mesquita Filho, Jaboticabal, 14884-900 Brazil; 4 Associação Nacional de Criadores e Pesquisadores (ANCP), Ribeirão Preto, 14020-230 Brazil; 5 Embrapa Cerrados, Planaltina, 73310-970 Brazil; 6 Zoetis, Kalamazoo, 49007 USA; 7 Universidade de São Paulo, Departamento de Nutrição e Produção Animal, Pirassununga, 13635-900 Brazil

**Keywords:** *Bos indicus*, Indicine, Genomic inbreeding, Gene ontology

## Abstract

**Background:**

The aim of this study was to assess genome-wide autozygosity in a Nellore cattle population and to characterize ROH patterns and autozygosity islands that may have occurred due to selection within its lineages. It attempts also to compare estimates of inbreeding calculated from ROH (F_ROH_), genomic relationship matrix (F_GRM_), and pedigree-based coefficient (F_PED_).

**Results:**

The average number of ROH per animal was 55.15 ± 13.01 with an average size of 3.24 Mb. The Nellore genome is composed mostly by a high number of shorter segments accounting for 78% of all ROH, although the proportion of the genome covered by them was relatively small. The genome autozygosity proportion indicates moderate to high inbreeding levels for classical standards, with an average value of 7.15% (178.70 Mb). The average of F_PED_ and F_ROH_, and their correlations (− 0.05 to 0.26) were low. Estimates of correlation between F_GRM_-F_PED_ was zero, while the correlation (− 0.01 to − 0.07) between F_GRM_-F_ROH_ decreased as a function of ROH length, except for F_ROH > 8Mb_ (− 0.03). Overall, inbreeding coefficients were not high for the genotyped animals. Autozygosity islands were evident across the genome (*n* = 62) and their genomic location did not largely differ within lineages. Enriched terms (*p* < 0.01) associated with defense response to bacteria (GO:0042742), immune complex reaction (GO:0045647), pregnancy-associated glycoproteins genes (GO:0030163), and organism growth (GO:0040014) were described within the autozygotic islands.

**Conclusions:**

Low F_PED-_F_ROH_ correlation estimates indicate that F_PED_ is not the most suitable method for capturing ancient inbreeding when the pedigree does not extend back many generations and F_ROH_ should be used instead. Enriched terms (*p* < 0.01) suggest a strong selection for immune response. Non-overlapping islands within the lineages greatly explain the mechanism underlying selection for functionally important traits in Nellore cattle.

**Electronic supplementary material:**

The online version of this article (10.1186/s12864-018-5060-8) contains supplementary material, which is available to authorized users.

## Background

Brazilian livestock and agriculture production have a prominent impact upon the world’s food commerce. Brazilian beef production is one of the largest players in the world and produced roughly 9.56 million tons of carcass weight equivalents in 2015 [1]. The vast majority of the bovine based population reared for meat production in Brazil is composed mostly of indicine cattle (*Bos taurus indicus*). According to the Brazilian Zebu Breeders Association (ABCZ, http://www.abcz.com.br) such population is around 80% of the total cattle. Given the physical and physiological characteristics that they possess which greatly explain their better adaptation towards grazing systems in tropical environments [2–4], it is not surprisingly that much use of the indicine cattle has been made in these regions.

The Nellore breed has the largest number of animals (horned and polled) among the indicine cattle raised in Brazil, followed by Guzerat and Gyr. Most of Nellore importation was from India during the last century and lasted up to the seventies when the importation was banned [5]. The Nellore population in Brazil is the result of less than 7000 heads of purebred imported animals [6]. The major importation took place in 1962, when exceptional bulls were brought over the country standing out as progenitors of the main Nellore lineages [7]. Magnabosco et al. [8] reported the existence of six predominant lineages of Nellore breed (Karvadi Imp; Taj Mahal Imp; Kurupathy Imp; Golias Imp; Godhavari Imp, and Rastã Imp) that contributed to the development of the current Brazilian Nellore population. These lineages were derived from outstanding bulls named Karvadi, Taj Mahal, Kurupathy, Golias, Godhavari and Rastã which gained fame as breeders given their high rates of productive and reproductive performance [7]. Although the selection criteria used to improve the Nellore cattle among Brazilian breeding programs are closely linked and mainly associated with reproductive and carcass quality traits, there is evidence of different genetic patterns among the lineages based on the selection criterion used to improve each of them over time [9, 10]. In this manner, a question can be raised whether the genetic progress is going or not towards the same direction within the lineages raised in Brazil.

Genetic evaluations of Nellore cattle using BLUP (Best Linear Unbiased Prediction) methodology have established significant progress since the eighties, when several genetic evaluation programs started to expand in Brazil [11]. Despite the reduced number of animals imported from India, Pereira et al. [12] have reported an average inbreeding coefficient of 3% in a Nellore population, indicating that these animals have been under relative control for at least three decades. Therefore, breeding programs are always seeking for strategies to preserve populations, and there is a growing interest in characterizing and monitoring genome-wide autozygosity to maintain the genetic diversity [13, 14], allowing a long-term conservation of genetic resources and sustainability in animal breeding programs.

Runs of homozygosis (ROH) have been widely applied to quantify individual autozygosity in livestock [15–20] given their high correlation (~ 0.7) [21]. A small number of studies have described the autozygosity in Nellore cattle and most of them do not make use of a large sample size. Karimi [22] identified region patterns with a high prevalence of ROH in taurine and indicine breeds and made use of merely 134 Nellore samples. Additionally, Zavarez et al. [19] reported the distribution of genome-wide autozygosity levels based on ROH in only 1278 Nellore cows genotyped for over 777,000 markers.

Since homozygous stretches printed on the genome may have arisen as a result of artificial selection, autozygosity based on ROH can strongly disclose the understanding of genetic selection [18]. ROH patterns are not seen to be randomly distributed across the genomes [23] and genomic regions sharing ROH patterns potentially contain alleles associated with genetic improvement in livestock [24]. The correlation of ROH and selection for productivity was first identified by Kim et al. [25]. Furthermore, ROH has been successfully utilized as a measure of inbreeding by estimating the level of autozygosity in the genome [15, 16, 25–28].

Up to date, studies characterizing genome-wide autozygosity in the main Nellore lineages are incipient. Hence, this study was carried out to assess genome-wide autozygosity in a Nellore cattle population to identify and characterize ROH patterns as well as to identify autozygosity islands that may have occurred due to selection for functionally important traits in different Nellore lineages and verify whether these lineages differ or not from one another. It attempts also to compare estimates of molecular inbreeding calculated from ROH (F_ROH_), genomic relationship matrix (F_GRM_), and from pedigree-based coefficient (F_PED_).

## Results

### Genome-wide distribution of runs of homozygosity

**Fig. 1:**
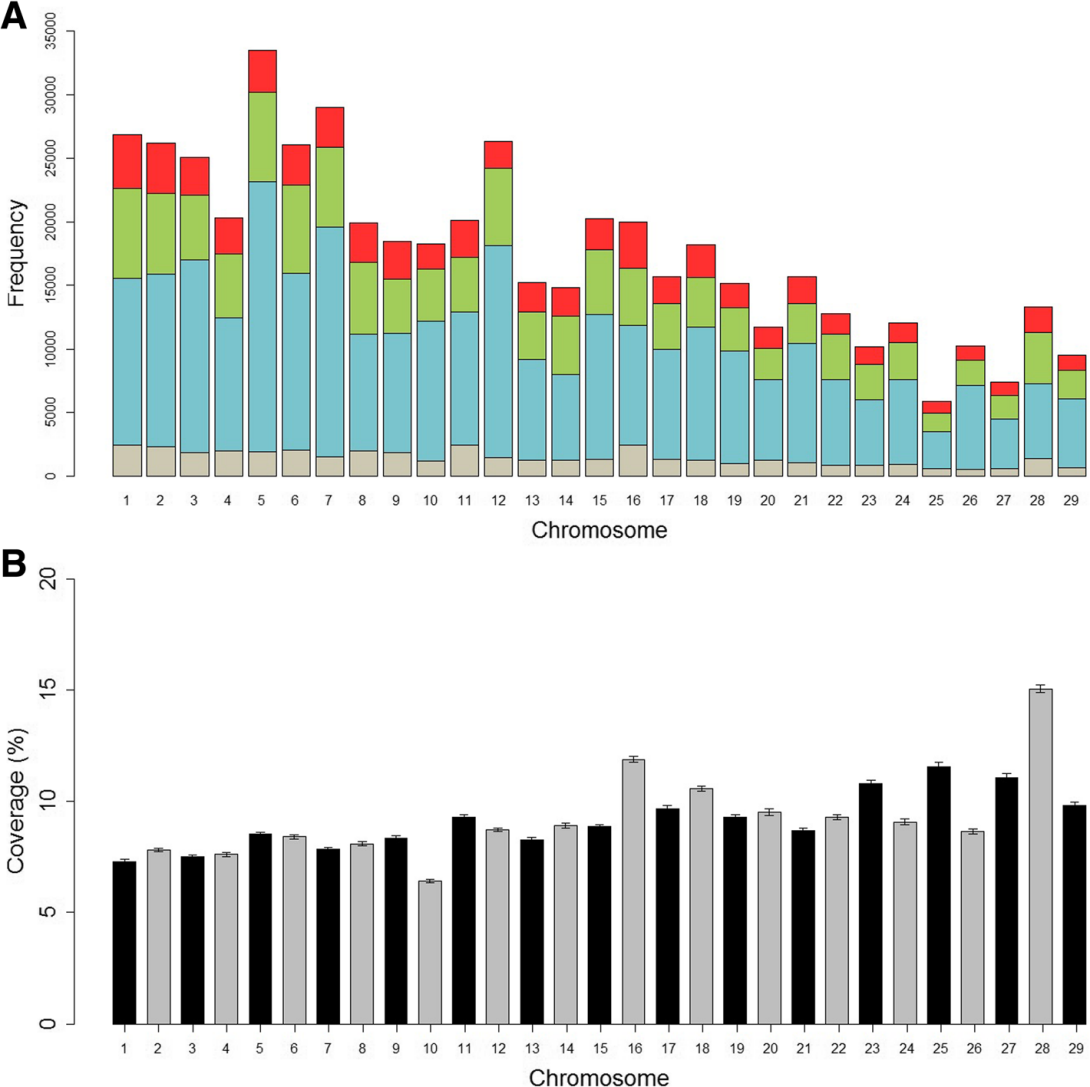
Runs of homozygosity distribution and coverage for each chromosome in Nellore cattle. **a**. Frequency distribution of the number of ROH in different length classes: blue (ROH_1–2 Mb_), green (ROH_2–4 Mb_), red (ROH_4–8Mb_), and grey (ROH_> 8 Mb_). **b**. Average percentage of chromosome coverage by runs of homozygosity of minimum length of 1 Mb. The error bars indicate standard error

On individual animal basis, the average number of ROH per animal, considering the genotyped animals (*n* = 9386), was 55.15 ± 13.01 with an average size of 3.24 Mb. The longest ROH was 99.30 Mb in length (28,778 SNPs) on *Bos Taurus* autosome (BTA) 5. The number of ROH per chromosome was also greater for BTA5 (33,492 segments) (Fig. 1a) and the greatest fraction of chromosome covered with ROH was found on BTA28 (15.06% of chromosomal length within an ROH) (Fig. 1b).

ROH analysis for the different length classes for the genotyped animals (*n* = 9386) revealed that the Nellore genome is composed mostly of a high number of shorter segments (ROH_1–2 Mb_ and ROH_2–4 Mb_), which accounted for approximately 78% of all ROH detected and roughly contributed to 43% of the cumulative ROH length (Table 1). Shorter and medium (ROH_4–8 Mb_) ROH displayed a similar genome coverage and also a cumulative ROH length, with values varying from 20.53 to 22.88%. Despite the total length of ROH being composed mostly of a high number of short segments, the proportion of the genome covered by them was relatively small when compared to larger ROH (ROH_> 8 Mb_).

**Table 1:** Descriptive statistics of runs of homozygosity number (*n*ROH) and length (in Mb) for four different length classes (ROH_1–2 Mb_, ROH_2–4 Mb_, ROH_4–8 Mb_, and ROH_> 8 Mb_)

Class	*n* ROH	(%)	Mean Length	Standard Deviation	Genome Coverage (%)	Cumulative ROH Length (%)
ROH_1–2 Mb_	285,085	55.07	1.34	0.27	1.63	22.88
ROH_2–4 Mb_	123,254	23.81	2.79	0.56	1.47	20.53
ROH_4–8 Mb_	68,407	13.21	5.53	1.11	1.63	22.59
ROH_> 8 Mb_	40,925	7.91	13.93	7.18	2.58	34.00

The most autozygous animal exhibited a ROH genome coverage encompassing 718.96 Mb of the total autosomal genome extension (UMD3.1) covered by markers (28.75% of the cattle genome), totaling 92 ROH ≥ ROH_1–2 Mb_. On average, 7.15% (178.70 Mb) of the genome was considered to be a region of homozygosity.

### Pedigree and genomic inbreeding

**Fig. 2:**
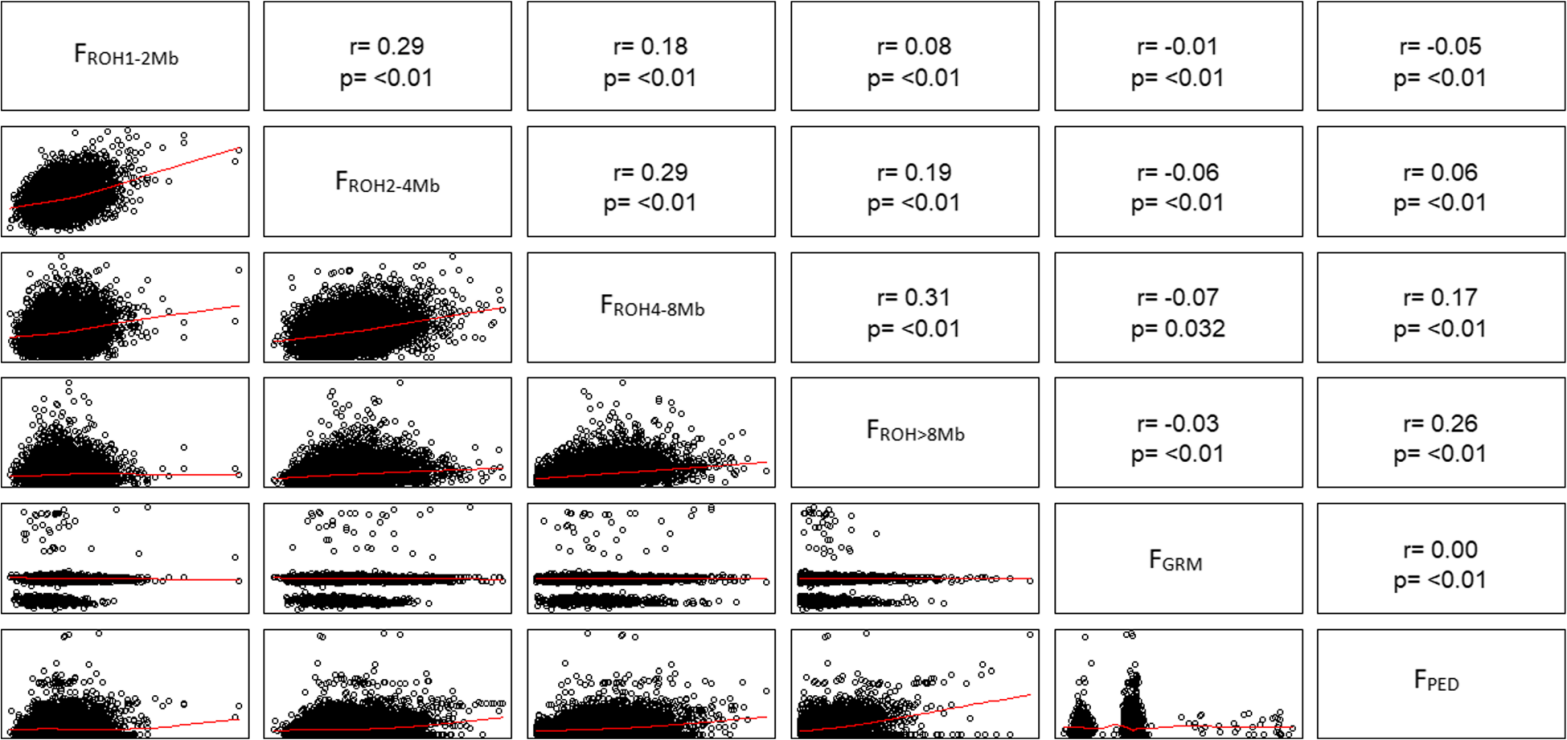
Scatterplots (lower panel) and Spearmann’s correlations (upper panel) of genomic inbreeding coefficients F_ROH_ (F_ROH 1–2 Mb_, F_ROH 2–4 Mb_, F_ROH 4–8 Mb_, and F_ROH > 8 Mb_) and F_GRM_, and pedigree-based inbreeding coefficients (F_PED_)

**Fig. 3:**
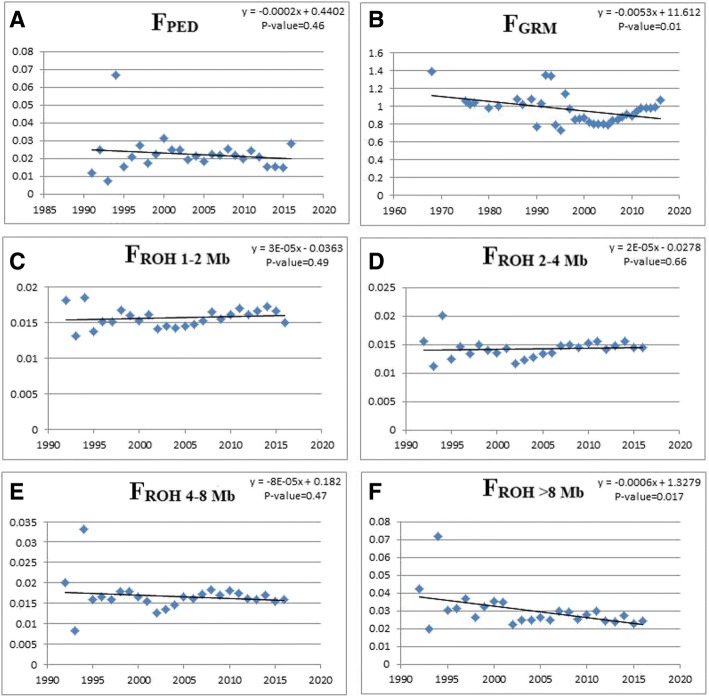
Inbreeding evolution over the past 30 years for pedigree-based inbreeding (F_PED_), genomic relationship matrix approach (F_GRM_), and F_ROH_ (F_ROH1–2 Mb_, F_ROH2–4 Mb_, F_ROH4–8 Mb_, and F_ROH > 8 Mb_) coefficients and their respective regression equations and *p*-values. The X-axis represents the years and the Y-axis shows the inbreeding coefficients. Each blue dot represents the inbreeding average per year

Descriptive statistics for F_PED_ and F_ROH_ coefficients for the genotyped animals (*n* = 9386) are presented in Table 2. The average F_PED_ and F_ROH_ were low in the studied population, and it is noteworthy that 94.20% of the genotyped animals exhibited a F_PED_ below 5%. Low correlations were observed between F_PED_-F_ROH_ and it gradually increased as a function of ROH length (Fig. 2). No estimates of correlation were found between F_GRM_-F_PED_ and those between F_GRM_-F_ROH_ decreased as a function of ROH length. The inbreeding evolution (Fig. 3) demonstrates a significant (*p* < 0.01) decay in F_GRM_ and F_ROH > 8 Mb_.

**Table 2:** Number of genotyped animals (*n*) and descriptive statistics of the pedigree-based inbreeding coefficient (F_PED_) and runs of homozygosity-based inbreeding coefficient (F_ROH_) for different lenghts (F_ROH1–2_, F_ROH2–4_, F_ROH4–8_, and F_ROH > 8 Mb_)

Coefficient	Mean	Median	Minimum	Maximum	Coefficient of Variation (%)	*n*
F_PED_	0.017	0.013	0.000	0.258	3.387	8502
F_ROH1–2 Mb_	0.016	0.016	0.000	0.199	27.14	9387
F_ROH2–4 Mb_	0.014	0.014	0.000	0.100	37.71	9352
F_ROH4–8 Mb_	0.016	0.015	0.001	0.059	47.81	9281
F_ROH > 8 Mb_	0.025	0.021	0.003	0.222	77.03	8836

F_PED_ and F_ROH_ averages for each Nellore lineage (*n* = 8646) are presented in Table 3. The highest F_PED_ (*p* < 0.05) values were observed for Karvadi, Golias, and Godhavari lineages. F_ROH_ estimates were close to F_PED_ and they did not differ (p < 0.05) for Karvadi and Godhavari lineages.

**Table 3:** Average mean (number of observations) of pedigree-based inbreeding coefficient (F_PED_) and runs of homozygosity-based inbreeding coefficient (F_ROH_) for different lenghts (F_ROH1–2_, F_ROH2–4_, F_ROH4–8_, and F_ROH > 8 Mb_) for six Nellore lineages

Coefficient	Karvadi	Golias	Godhavari	Taj Mahal	Akasamu	Nagpur
F_PED_^1^	0.020^a^ (7282)	0.019^a^ (178)	0.020^a^ (90)	0.016^ab^ (103)	0.011^b^ (42)	–
F_ROH1–2 Mb_	0.016^a^ (7853)	0.014^c^ (288)	0.015^ab^ (205)	0.014^bc^ (149)	0.014^c^ (79)	0.014^c^ (50)
F_ROH2–4 Mb_	0.014^a^ (7810)	0.012^b^ (284)	0.014^a^ (198)	0.012^b^ (144)	0.011^b^ (73)	0.012^b^ (44)
F_ROH4–8 Mb_	0.015^a^ (7664)	0.014^b^ (266)	0.016^a^ (185)	0.014^b^ (136)	0.014^b^ (70)	0.012^b^ (40)
F_ROH > 8 Mb_	0.025^a^ (7443)	0.022^bc^ (245)	0.024^ab^ (171)	0.018^c^ (130)	0.022^bc^ (70)	0.017^c^ (34)

F_PED_ was not available for the Nagpur lineage. Means sharing a common letter within a row were not significantly different (*p* < 0.05) from one another

### Autozygosity islands in Nellore lineages

Autozygosity islands were evident across the genome, and their distributions along the genome vary in length and position across chromosomes. A total of 62 regions with 100 outlying consecutive SNPs were identified for the genotyped animals (*n* = 9386) in almost all autosomes, with the exception of BTA2, BTA11, BTA18, BTA25, and BTA28 (Additional file 1). Overall, the mean length was 1.40 ± 0.85 Mb, and the longest island was observed on BTA7 (107,000,000:111,700,000 bp) encompassing 4.70 Mb of length. Interestingly, BTA7 also contained the highest number of islands (*n* = 8) followed by BTA1, BTA12 and BTA20, all-encompassing five islands each.

To verify if the autozygosity islands possess genes related to environmental adaptation processes, those 62 autozygosity islands were overlapped with 9803 CNVRs strongly associated with adaptation for Nellore cattle described by Lemos et al. [29]. Only 338 CNVRs were observed within the autozygosity islands, and the overlapping regions harbored 484 genes with described functions.

When analyzing the autozygosity islands within the lineages (*n* = 8646), the Karvadi lineage showed the highest number of islands (*n* = 54), followed by Godhavari (*n* = 31), Golias (*n* = 26), Taj Mahal (*n* = 18), Akasamu (*n* = 13) and Nagpur (*n* = 6). It should be noted that overlapping islands were observed in between the lineages (Additional files 2 and 3). Interestingly, the region on BTA7 encompassing 51,610,000 to 52,930,000 bp in length was found to be described in all lineages. Non-overlapping autozygosity islands were also observed in some lineages in specific genomic regions and were screened for gene content (Additional file 4). These regions could be an indicative of selection signatures or it may reflect inbreeding events within a lineage [26].

### Functional annotation of genes

As most of autozygosity islands identified for the genotyped animals (*n* = 9386) overlapped with those described for the Nellore lineages (Additional file 5), the analysis performed using the DAVID v.6.8 [30, 31] comprised 946 genes identified for the genotyped animals (Table 4). Additional file 6 describes the set of genes involved in each GO term and KEGG pathway.

**Table 4:** Gene Ontology (GO) terms and KEGG pathways annotation analysis enriched (*P* < 0.01) based on autozygosity islands set of genes

Terms	Genes	*P*-value
GO Biological Process		
(GO:0042742) Defense response to bacteria	14	7.07E-5
(GO:0030163) Protein catabolic process	9	6.33E-4
(GO:0070200) Establishment of protein localization to telomere	4	1.70E-3
(GO:0040014) Regulation of multicellular organism growth	6	2.68E-3
(GO:0045647) Negative regulation of erythrocyte differentiation	4	4.46E-3
(GO:0030901) Midbrain development	6	4.84E-3
GO Molecular Function		
(GO:0008289) Lipid binding	13	2.07E-4
(GO:0004190) Aspartic-type endopeptidase activity	9	3.24E-4
GO Cellular Component		
(GO:0005776) Autophagosome	8	3.07E-3
(GO:0005634) Nucleus	155	6.11E-3
(GO:0005815) Microtubule organizing center	10	8.36E-3
(GO:0005730) Nucleolus	41	8.50E-3
KEGG pathway		
(bta01100) Metabolic pathways	72	4.21E-4

To obtain a broad functional insight into the set of genes (*n* = 484) observed within the autozygosity islands and CNVRs overlapping regions, an enrichment analysis was also performed. An enhancement of genes involved in several GO terms (four biological processes, one molecular function, and none cellular component process) was significant (*p* ≤ 0.01) and one for KEEG (Additional file 7). Despite the large number of overlapping regions, and consequently, the large number of genes found in these regions, no significant GO term and KEGG pathway was found commonly associated in both studies and neither associated in some way with environmental adaptation processes.

## Discussion

### Genome-wide distribution of runs of homozygosity

The longest ROH was described on BTA5, however, results in taurine and indicine cattle [20, 25, 32] have reported the longest on BTA8. Corroborating with the results, Peripolli et al. [20] observed the greatest number of ROH on BTA5 in indicine cattle, however, studies have described the greatest number on BTA1 [24, 32, 33]. BTA5, which presented the longest and the greater number of ROH, has been reported to harbor QTL related to weight [34, 35], reproduction [36, 37], and milk fat yield traits [37, 38] in cattle.

**Fig. 4:**
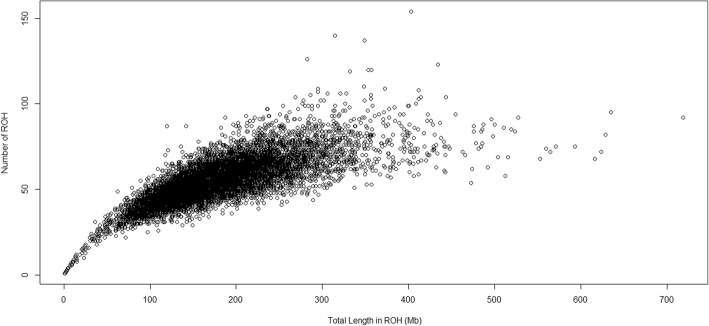
Relationship between the number of runs of homozygosity (ROH) per individual and the total length of the genome covered by them. Each hollow circle stands for one animal

Dissimilarity among animals was observed between the number of ROH and the length of the genome covered by ROH (Fig. 4). Animals exhibiting the same homozygous genome length displayed a variable number of ROH. This pattern was also described by Mészáros et al. [39], who attributed this event as a consequence of the distinct distances from the common ancestor. Therefore, when considering animals with the same homozygous genome length, we can infer that those displaying more ROH have an increased distance with the common ancestor since these segments are expected to be shorter due to repeated meiosis events that break up ROH through recombination [40].

The highest autozygosity value per animal was similar to those reported in the literature for dairy breeds [20, 24, 32, 41]. Conversely, Marras et al. [18] described that dairy breeds had a higher sum of all ROH than did beef breeds, and Purfield et al. [24] observed that dairy breeds were the most autozygous animals among several studied breeds. In addition, the autozygotic proportion of the genome described for this population seems to indicate moderate to high inbreeding levels for classical standards. Similar results were described by Marras et al. [18] for Marchigiana beef cattle (7%) and Peripolli et al. [20] for Gyr dairy cattle (7.10%). Compared to Zavarez et al. [19] study on a Nellore population whose findings showed a value of 4.58%, this sample of Nellore animals presented a higher average autosomal coverage. The high autozygosity value per animal and homozygous proportion of the genome observed for this population might be a result of the small number of imported progenitors to speed up the genetic progress and develop the first Nellore lineages during the major importation in the sixties. Furthermore, the formation of lineages can be made by the use of consanguinity in which the same breeder is mated with its descendants along the generations aiming to fix genes related to important traits [8].

### Pedigree and genomic inbreeding

F_PED_ was lower than results reported by Barbosa et al. [42] and higher than those described by Santana et al. [43], with values of 8.32% and 1.42% for inbred Nellore populations, respectively.

F_ROH_ can disclose the age of the inbreeding given the approximate correlation between the length of the ROH and the distance with the common ancestor due to recombination events over time. Therefore, calculated F_ROH_ are expected to correspond to the reference ancestral population dating 50 (F_ROH1–2 Mb_), 20 (F_ROH2–4 Mb_), 12.5 (_FROH4–8 Mb_), and 6 (F_ROH > 8Mb_) generations ago by considering that 1 cM equals to 1 Mb [44]. According to Zavarez et al. [19], incomplete pedigree cannot account for inbreeding caused by distant ancestors and estimates based on F_PED_ are only comparable with F_ROH_ calculated over large ROH. F_PED_ estimate was then compared with F_ROH > 8 Mb_, and the genome autozygotic proportion from F_ROH > 8 Mb_ exceeded F_PED_. This variation can be attributed to the fact that the pedigree might not have been deep enough to allow F_PED_ to capture the relatedness since its average depth is close to four generations, whereas F_ROH > 8 Mb_ reflects an inbreeding that occurred nearly six generations ago. Furthermore, F_PED_ does not take into account the stochastic events of recombination during meiosis [26] and pedigree relatedness does not show the actual relatedness among individuals since it is estimated from statistical expectations of the probable identical by descendent (IBD) genomic proportion [45].

F_PED_-F_ROH_ correlations were seen to be higher when longer ROH reflecting recent relatedness were included in F_ROH_ estimates. It is noticeable to highlight that most of the pedigree records did not extend back many generations, therefore, correlations with shorter ROH reflecting ancient relatedness tended to be lower and those with longer ROH reflecting recent relatedness had a tendency to be higher [18, 46]. Additionally, several authors have reported a high correlation between F_PED_-F_ROH_ when a deeper number of described generations are available in the pedigree [15, 16, 18, 24, 33].

No estimates of correlation between F_GRM_-F_PED_ may be explained by considering that individuals from sub-populations for which allele frequencies diverge from the entire population may have been estimated to have high F_GRM_ [47], which may have led to biased correlation. According to Zhang et al. [48], inbreeding coefficients based on methods using allele frequency are sensitive compared to ROH-based methods, especially for populations with divergent allele frequencies. Correlations between F_GRM_-F_ROH_ decreased as a function of ROH length, and Zavarez et al. [19] associated it with the properties of the G matrix, which is based on individual loci, whereas F_ROH_ is based on chromosomal segments.

The inbreeding evolution stress out a significant (*p* < 0.01) decline in F_ROH > 8 Mb_ and it is worth highlighting that it reflects inbreeding up to six generations prior (~ 30 years). The reduction in this coefficient since the 1990’s happened together with the foundation of the Nellore Brazil Breeding program in 1988 (ANCP, http://www.ancp.org.br). These results pointed out, that mating decisions were taken since this time by the breeders to avoid mating between relatives, decreasing the genomic inbreeding level in this population over time. The F_ROH 4–8 Mb_ reflects inbreeding up to 12.5 generations prior (~ 60 years) and the slight reduction in this coefficient since the 1960’s happened together with the beginning of bull evaluation for weight gain in test stations. The results obtained for F_ROH1–2 Mb_ and F_ROH2–4 Mb_ showed that mating decisions before the major importations might have favored the increasing of inbreeding.

Inbreeding coefficients were not high for the genotyped animals with lineages records (*n* = 8646), with values around to 2%. According to Pereira [49], the lineage diversification within a breed can provide substantial gains for selection by reducing inbreeding rates and restoring the genetic variability. The use of Karvadi, Golias, and Godhavari lineages can be evidenced by the high inbreeding rates described for them when compared to other lineages. According to Oliveira et al. [7], when considering a small number of progenitors in a studied breed, the prevalence use of some ancestors can be explained by their marginal contribution in the reference population. Hence, when assessing the marginal contribution of each lineage to the ANCP Nellore cattle population, an eminent contribution of Karvadi and Godhavari lineages can be observed (10.44 and 1.48%, respectively), agreeing with F_ROH_ estimates. Lineages such as Golias, Taj Mahal, Akasamu, and Nagpur did not show an expressive marginal contribution, and interestingly, displayed lower inbreeding averages (*p* < 0.05) for F_ROH1–2 Mb_, F_ROH2–4 Mb_, and F_ROH4–8 Mb_.

### Autozygosity islands in Nellore lineages

Autozygosity islands in the genotyped animals (*n* = 9386) were seen overlapping with previous studies on several cattle breeds (Additional file 8). Within these studies, islands were not reported overlapping only with those described for Nellore cattle. Remarkably, Sölkner et al. [50] and Szmatoła et al. [41] displayed islands in common on BTA7 encompassing the same chromosomal region around 51–53 Mb, and Szmatoła et al. [41] also described islands located on the same chromosomal region on BTA7 (42–44 Mb) in Holstein, Red Polish, Simmental and Limousin cattle breeds. Sölkner et al. [50] and Gaspa et al. [51] exhibited overlapping islands around 1.3–1.9 Mb on BTA21. Overlapping islands between these studies and the current one (43,510,000:43,592,173 – BTA7; 51,574,295:52,353,000 - BTA7, and 1,360,390: 1,829,761 – BTA21) were inspected in detail. These islands are suggested to harbor targets of positive selection in cattle [52] and may be used to identify regions of the genome under selection, and to map genes that affect traits of interest [18]. Further, ROH islands were found overlapping in cattle breeds selected for different purposes, suggesting that selection pressure can also be undergoing on traits other than those specific to dairy or beef traits.

When examining in detail, the region encompassing 51–52 Mb on BTA7 harbored relevant genes for beef cattle production. Among them, we highpoint the *CTNNA1* gene which has been associated with myostatin expression level in skeletal muscle of Holstein-Friesian bulls [53]. Myostatin is a key protein that plays an essential role in regulating skeletal muscle growth, and it is considered to be one of the most important factors responsible for meat productivity traits in cattle [54]. The *MATR3* gene was also described within the overlapping region and has been related to fat deposition in cattle [55, 56]. It is also worth highlighting the *ECSCR* gene. This gene regulates insulin sensitivity and predisposition to obesity [57]. Besides, the protein encoded by this gene is primarily found in endothelial cells and blood vessels (provided by RefSeq, Jun 2014). Endothelial cells are the important players in angiogenesis, a physiological process by which new blood vessels develop from pre-existing vasculature [58]. Blood vessels dilate to dissipate heat to external environment by a process denominated vasodilation. In this regard, the *ECSCR* gene might be a key role in elucidating the better tolerance of some cattle breeds to heat stress, i.e. *Bos taurus indicus*. The increased number of blood vessels through the angiogenic process allows more blood to be dissipated, decreasing the body temperature.

Overlapping islands within the lineages (*n* = 8646) were described in this study and two reasons might have leaded to this result. First, the Nellore cattle sampled in Brazil is derived from the Ongole cattle imported from the Indian district of Andhra Pradesh [59]. Prior such importations, the Ongole cattle was already notorious in India due to their greater adaptation upon high temperatures, ability to carry lower burdens of cattle tick and tolerate poor feed management [60]. Therefore, these overlapping regions might reflect the acquired adaptedness of zebu cattle in tropical environments due to natural selection over the time [61]. Second, these findings support the concept that despite having different lineages within the Nellore breed, the genetic progress of economically important traits goes toward the same direction and IBD genomic regions harboring traits of interest are being conserved over time.

The region on BTA7 described to be overlapping in all lineages (51,610,000:52930000 bp) harbored five genes (*CTNNA1*, *LRRTM2*, *SIL1*, *MATR3*, *and PAIP2*). Among them, the *CTNNA1* (Catenin Alpha 1) gene has been described associated with myostatin expression level and molecular function in skeletal muscle in Holstein-Friesian bulls [53]. Furthermore, the *LRRTM2* (Leucine Rich Repeat Transmembrane Neuronal 2) gene was found related to maturation of male germ cells and male fertility [62, 63].

Non-overlapping islands within the lineages were explored for gene content and among the genes identified within the regions we can highpoint those described in Table 5. Remarkably, six genes were also reported in Nellore-specific studies associated with carcass traits [64] (*PPM1*), age at first calving [65] (*NPBWR1, OPRK1, and MRPL1*), and birth weight [66] (*RPS20 and TGS1*).

**Table 5:** Gene content of non-overlapping ROH islands within the Nellore lineages highlighted according to their function

Lineage	Gene	Function	Author
Godhavari	*LAMB4*	Immune System	[91]
Karvadi	*RFX4*	Immune System	[92]
Godhavari	*IFRD, PPM1B, DTX4, MTMR7*	Productive traits	[64, 92–95]
Taj Mahal	*CAPZA2*	Productive traits	[96]
Karvadi	*ZBTB20, RPS20, STAC3, STAT6, RIC8B, LYPLA1, XKR4, TMEM68, TGS1*	Productive traits	[66, 92, 97–102]
Godhavari	*NAMPT*	Reproductive traits	[103, 104]
Godhavari	*PPM1B, JMJD1C*	Reproductive traits	[105, 106]
Karvadi	*RFX4, NPBWR1, OPRK1, MRPL15*	Reproductive traits	[65, 107]
Karvadi	*DRD3, ZBTB20*	Reproductive traits	[108, 109]
Karvadi	*CSNK1A1, TBC1D12*	Thermotolerance	[110, 111]

Despite having non-overlapping autozygosity islands within the lineages, several genes have been found described associated with productive and reproductive traits within the lineages. Productive related-genes were mainly associated with average daily gain (*IFRD1*), muscle (*PPM1B* and *STAC3*), fat (*DTX4* and *XKR4*), body and birth weight *(MTMR7*, *RPS20*, and *TGS1*), meat and carcass quality traits (*MTMR7*, *CAPZA2*, *STAT6*, and *RIC8B*), and feed intake (*LYPLA1* and *TMEM68*). Reproductive related-genes largely encompassed those linked to heifer’s fertility (*RFX4*), age at first calving (*NPBWR1*, *OPRK1*, and *MRPL15*), and oocyte maturation and expression (*NAMPT* and *JMJD1C*).

Although they were not located in the same genomic regions, these autozygosity islands showed an enrichment of genes involved in cattle growth, meat and carcass quality traits, immune system, and thermotolerance functions. These findings help to reinforce the concept that the genetic progress goes towards the same direction within the lineages and different genetic patterns among the lineages based on the selection criterion used to improve each of them could not be identified in this study.

### Functional annotation of genes

The analyses performed on DAVID revealed only the metabolic pathways (bta01100) KEGG pathway as significant (*p* < 0.01), while the Gene Ontology analyses showed several enriched terms for the ROH gene list. The defense response to bacteria (GO:0042742) on biological process encompasses several reactions triggered in response to the presence of a bacteria that act to protect the cell or organism. We highlighted the beta-defensin genes (*DEFB1, DEFB4A, DEFB5, DEFB6, DEFB7, DEFB10,* and *DEFB13*) that encode host defense peptides that are critical to protection against bacterial, viral and fungal infections, and acts as an important link between innate and adaptive immune responses [67]. In addition to their antimicrobial properties, beta-defensins have an important role in several functions including regulation of the immune response, fertility, reproduction, and embryo development [67, 68].

The negative regulation of erythrocyte differentiation (GO:0045647) on biological process is defined as any process that stops, prevents, or reduces the frequency, rate or extent of erythrocyte differentiation. Erythrocytes were described by Nelson [69] as belonging to the immune complex reaction (bacteria, complement, and antibody). In fish and chickens, erythrocytes have been shown to facilitate the clearance of pathogens by macrophages [70], and could produce specific signaling molecules such as cytokines in response to binding [71, 72].

The protein catabolic process (GO:0030163) includes chemical reactions and pathways resulting in the breakdown of mature proteins, which play an important role in the immune and inflammatory response. Khansefid et al. [73] identified the protein catabolic process enriched in genes significantly associated with residual feed intake in Angus and Holstein cattle breeds. Regarding the genes related to protein catabolic process identified in our study, most of them are pregnancy-associated glycoproteins genes (PAG) (Supplementary file 6) mapped on BTA29. Goszczynski et al. [74] identified eight genes belonging to the PAG gene family within ROH islands in Retinta cattle breed, while Szmatoła et al. [41] identified sixteen PAG genes in Holstein cattle breed. PAG glycoproteins are one major group of the proteins secreted from trophoblast cells of the placenta into the maternal blood shortly after implantation and are detectable throughout gestation [56]. These proteins have been used to monitor embryonic viability as biochemical pregnancy markers in the cow’s blood or milk [75] as well as placental functions in cattle [76, 77]. Significant reductions in PAG concentrations during the late embryonic/early fetal period are associated with pregnancy failures in cattle [76, 78]. PAG proteins also play an important role in implantation, placentogenesis, fetal antigen sequestering, and fetal–maternal interactions [76, 79–81]. Modifications in circulating PAG concentrations also were associated with several parameters linked to pregnancy loss in cattle, including parity, artificial insemination service number, milk yield, and metabolic diseases [82].

The regulation of multicellular organism growth (GO:0040014) biological process encompasses any process that modulates the frequency, rate or extent of growth of the body of an organism so that it reaches its usual body size, while the midbrain development (GO:0030901) biological process encompass the process whose specific outcome is the progression of the midbrain over time, from its formation to the mature structure.

## Conclusions

This study is the first of its kind to bring out results characterizing genome-wide autozygosity in the main Nellore lineages. The average F_PED_ and F_ROH_ of different lengths were low in the studied population, however, the autozygotic proportion in the genome indicates moderate to high inbreeding levels. Low correlations between F_PED-_F_ROH_ may be partly due to the relatively superficial depth of the pedigree, emphasizing the concept that autozygosity based on ROH should be used as an accurate estimator of ancient individual inbreeding levels [15, 24, 33, 83]. Overall, inbreeding coefficients were not high within the lineages and the findings obtained in this study suggest that lineages displaying an eminent marginal contribution in the reference population also display the highest F_ROH_ values, i.e. Karvadi and Godhavari.

Genomic regions that are selection targets tend to generate autozygosity islands and several of them have been described in the Nellore genome. Most remarkable is the clear evidence of autozygosity islands patterns within the lineages, suggesting that IBD genomic regions have been selected for the same traits over time. Autozygosity islands harbored enriched terms in which we highlight the defense response to bacteria (GO:0042742) and the negative regulation of erythrocyte differentiation (GO:0045647), which might help to better elucidate the greater adaptation of indicine cattle in host environment given its association with immune responses mechanisms. Additionally, non-overlapping autozygosity islands within the lineages were found to contain genes related to cattle growth, reproduction, and meat and carcass quality traits. The results of this study give a comprehensive insight about the autozygosity patterns in the main Nellore lineages and their potential role in explaining selection for functionally important traits in cattle. Despite having different lineages within the Nellore breed, it has clearly shown that selection is going towards the same direction and different genetic patterns could not be described.

## Methods

### Animals and genotyping

The animals used in this study comprise a dataset and progeny test program from the National Association of Breeders and Researchers (ANCP – Ribeirão Preto-SP, Brazil). The progeny test program headed by ANCP aims to disseminate semen of genetically superior Nellore young bulls evaluated for sexual precocity, growth, morphologic composition, feed efficiency, and carcass quality traits.

Nellore animals were genotyped with the low-density panel (CLARIFDE® Nelore 2.0) containing over 20,000 markers (*n* = 7729 animals); GGP-LD BeadChip (GeneSeek® Genomic Profiler 30 K) that contains 30,106 markers (*n* = 201 animals); Illumina BovineSNP50® Beadchip (Illumina Inc., San Diego, CA, USA) containing 54,001 markers (*n* = 58 animals); GGPi BeadChip (GeneSeek® Genomic Profiler Indicus) that contains 74,153 markers (*n* = 487 animals); and with Illumina BovineHD BeadChip (Illumina Inc., San Diego, CA, USA) containing 777,962 markers (*n* = 911 animals). Imputation was implemented using the FIMPUTE 2.2 software [84] and all genotypes were imputed to a panel containing 735,044 markers. A reference population with 963 sires and dams genotyped with the Illumina BovineHD BeadChip (Illumina Inc., San Diego, CA, USA) was used. Prior imputation, markers were edited for call rate (< 90%) for the genotyped and the reference populations. SNPs unsigned to any chromosome and those assigned to sexual chromosomes were removed from the dataset. After editing, a total of 9386 animals and 735,044 SNP markers were retained for the analyses. Genotyped animals with lineages records (*n* = 8646) were categorized as follows: Karvadi Imp (*n* = 7860), Golias Imp (*n* = 290), Godhavari Imp (*n* = 210), Taj Mahal Imp (*n* = 150), Akasamu Imp (*n* = 81), and Nagpur Imp (*n* = 55). Lineages were classified using the PEDIG package [85], which estimates the average consanguinity between a set of individuals and a reference group. The reference group encompassed founder’s animals from the Nellore base population in which the Nellore lineages were derived from.

### Runs of homozygosity

Individual ROH was identified using PLINK v1.90 software [86], which uses a sliding window approach to scan each individual’s genotype at each marker position to detect homozygous segments [44]. The parameters and thresholds applied to define ROH were set as follows: a sliding window of 50 SNPs across the genome, a minimum number of 100 consecutive SNPs included in a ROH, a minimum ROH length of 1 Mb, a maximum gap between consecutive homozygous SNPs of 0.5 Mb, one SNP per 50 kb, and a maximum of five SNPs with missing genotypes and up to one heterozygous genotype in a ROH. ROH were classified into four length classes: 1–2, 2–4, 4–8, and > 8 Mb, identified as ROH_1–2 Mb_, ROH_2_–_4 Mb_ ROH_4–8 Mb_, and ROH_> 8 Mb,_ respectively. ROH were performed separately for all genotyped animals (*n* = 9386) and for each Nellore lineage (*n* = 8646).

### Pedigree and genomic inbreeding coefficients

Pedigree-based inbreeding coefficients (F_PED_) were estimated using pedigree records from a dataset containing 45,917 animals born between 1934 and 2017. The pedigree dataset was provided by the National Association of Breeders and Researchers (ANCP – Ribeirão Preto-SP, Brazil). The average pedigree depth was approximately four generations, with a maximum depth value of nine. The F_PED_ was estimated for both datasets (n = 9386 and n = 8646) through the software INBUPGF90 [87]. Genomic inbreeding coefficients based on ROH (F_ROH_) were estimated for each animal and both datasets, according to the genome autozygotic proportion described by McQuillan et al. [21]:

$$ {F}_{ROH}=\frac{\sum_{j=1}^n{L}_{ROH j}}{L_{total}} $$

where L_ROHj_ is the length of ROH_j_, and L_total_ is the total size of the autosomes covered by markers. L_total_ was taken to be 2,510,605,962 bp, based on the consensus map. For each animal, F_ROH_ (F_ROH1–2 Mb_, F_ROH2–4 Mb_, F_ROH4–8 Mb_, and F_ROH > 8 Mb_) was calculated based on ROH distribution of four minimum different lengths (ROH_j_): 1–2, 2–4, 4–8, and > 8 Mb, respectively. A second measure of genomic inbreeding was calculated just for the whole dataset (n = 9386) using the Genomic relationship matrix (G) (F_GRM_). The G matrix was calculated according to VanRaden et al. [88] as follows:

$$ G=\frac{ZZ^{\hbox{'}}}{2{\sum}_{i=1}^n{P}_i\left(1-{P}_i\right)} $$

where Z is a genotype matrix that contains the 0-2*p* values for homozygotes, 1–2*p* for heterozygotes, and 2-2*p* for opposite homozygotes, where *P*_*i*_ is the reference allele frequency at locus *i*th. The diagonal elements of the matrix G represent the relationship of the animal with itself, thus, it was used to assess the genomic inbreeding coefficient. Spearman method was used to estimate correlations between the inbreeding measures.

### Identification and gene prospection in autozygosity islands

Autozygosity islands were defined as regions where SNPs were outliers according to boxplot distribution for each autosome (Additional files 9 and 10). A file generated by PLINK v1.90 software [86] which specifies how many times each SNP appeared in an ROH was used and regions displaying at least 100 consecutive outlier SNPs were then classified as an autozygosity island. Raw data regarding how many times each SNP appeared in an ROH was log-transformed (Log_10_). Autozygosity islands were identified separately for all genotyped animals (n = 9386) and for each Nellore lineage (n = 8646).

The gene content of the autozygosity islands was identified using the UMD3.1 bovine genome assembly from the Ensembl BioMart tool [89]. Database for Annotation, Visualization, and Integrated Discovery (DAVID) v6.8 tool [30, 31] was used to identify significant (*p* ≤ 0.01) Gene Ontology (GO) terms and KEGG (Kyoto Encyclopedia of Genes and Genomes) pathways using the list of genes from autozygosity islands and the *Bos taurus taurus* annotation file as background.

Autozygosity islands previously identified for the genotyped animals were overlapped with copy number variation regions (CNVRs) described for Nellore cattle by Lemos et al. [29]. Overlap analysis was carried out using the Bioconductor package *GenomicRanges* [90].

## Additional files

Additional file 1: Autozygosity islands across the Nellore cattle genome. (DOCX 20 kb)

Additional file 2: Autozygosity islands within the Nellore lineages by chromosome: Karvadi (red), Golias (Black), Godhavari (Green), Taj Mahal (blue), Akasamu (purple), and Nagpur (yellow). (PDF 31 kb)

Additional file 3: Overlapping autozygosity islands within the Nellore lineages. (DOCX 28 kb)

Additional file 4: Non-overlapping autozygosity islands within the Nellore lineages. (DOCX 26 kb)

Additional file 5: Autozygosity islands within the genotyped animals (red) and those with lineages records (black). (PDF 29 kb)

Additional file 6: Gene Ontology terms and KEGG pathways annotation analysis enriched (*P* < 0.01) based on autozygosity islands set of genes identified for the genotyped animals (*n* = 9386). (DOCX 17 kb)

Additional file 7: Gene Ontology terms annotation analysis enriched (P < 0.01) based on copy number variation regions (CNVRs) and autozygosity islands overlapping regions set of genes identified for the genotyped animals (*n* = 9386). (DOCX 15 kb)

Additional file 8: Runs of homozygosity islands described in several cattle breeds located within those observed in the present study. (DOCX 22 kb)

Additional file 9: Outliers SNPs for the genotyped animals (n = 9386) according to Boxplot distribution. (PDF 227 kb)

Additional file 10: Outliers SNPs for each Nellore lineage (*n* = 8646) according to Boxplot distribution. (PDF 340 kb)

## Abbreviations

ANCP: Associação Nacional de Criadores e Pesquisadores
BLUP: Best Linear Unbiased Prediction
BTA: *Bos taurus* autosome
CNV: Copy number variation
CNVRs: Copy number variation regions
DAVID: Database for Annotation, Visualization, and Integrated Discovery
F_GRM_: Genomic relationship matrix-based estimates of inbreeding
F_PED_: Pedigree-based estimates of inbreeding
F_ROH_: ROH-based estimates of inbreeding
G: Genomic relationship matrix
GO: Gene Ontology
IBD: Identical by descent
KEEG: Kyoto Encyclopedia of Genes and Genomes
NCBI: National Center for Biotechnology Information
QTL: Quantitative trait loci
ROH: Runs of homozygosity
SNP: Single Nucleotide Polymorphism
