# Supplementary material for: Autozygosity islands and ROH patterns in Nellore lineages: evidence of selection for functionally important traits
Source: BMC Genomics. 2018 Sep 17;19:680. doi: 10.1186/s12864-018-5060-8 (PMC6142381; doi:10.1186/s12864-018-5060-8)
Supplement: Supplementary file 1 — Autozygosity islands across the Nellore cattle genome. (DOCX 20 kb) [file 12864_2018_5060_MOESM1_ESM.docx]

| Additional 1: Autozygosity islands across the genome of Nellore cattle | | | |
| --- | --- | --- | --- |
| **BTA^1^** | **Start (bp)** | **End (bp)** | **Length (bp)** |
| 1 | 850,000 | 2,464,000 | 1,614,000 |
| 1 | 30,950,000 | 32,090,000 | 1,140,000 |
| 1 | 39,450,000 | 40,200,000 | 750,000 |
| 1 | 40,340,000 | 40,870,000 | 530,000 |
| 1 | 59,210,000 | 60,300,000 | 1,090,000 |
| 3 | 66,110,000 | 67,510,000 | 1,400,000 |
| 3 | 75,810,000 | 77,440,000 | 1,630,000 |
| 3 | 101,400,000 | 101,800,000 | 400,000 |
| 4 | 54,280,000 | 55,800,000 | 1,520,000 |
| 5 | 47,000,000 | 48,130,000 | 1,130,000 |
| 5 | 56,360,000 | 57,500,000 | 1,140,000 |
| 5 | 70,060,000 | 71,090,000 | 1,030,000 |
| 6 | 80,560,000 | 81,390,000 | 830,000 |
| 7 | 21,390,000 | 22,480,000 | 1,090,000 |
| 7 | 39,680,000 | 40,180,000 | 500,000 |
| 7 | 43,510,000 | 44,180,000 | 670,000 |
| 7 | 44,450,000 | 46,300,000 | 1,850,000 |
| 7 | 51,140,000 | 54,040,000 | 2,900,000 |
| 7 | 62,370,000 | 63,450,000 | 1,080,000 |
| 7 | 84,410,000 | 85,140,000 | 730,000 |
| 7 | 107,000,000 | 111,700,000 | 4,700,000 |
| 8 | 25,940,000 | 28,340,000 | 2,400,000 |
| 9 | 3,971,000 | 5,182,000 | 1,211,000 |
| 10 | 45,560,000 | 46,090,000 | 530,000 |
| 10 | 52,840,000 | 55,180,000 | 2,340,000 |
| 12 | 25,540,000 | 27,080,000 | 1,540,000 |
| 12 | 27,350,000 | 30,040,000 | 2,690,000 |
| 12 | 34,990,000 | 37,070,000 | 2,080,000 |
| 12 | 37,080,000 | 39,800,000 | 2,720,000 |
| 12 | 56,790,000 | 57,850,000 | 1,060,000 |
| 13 | 50,200,000 | 50,960,000 | 760,000 |
| 13 | 62,650,000 | 66,140,000 | 3,490,000 |
| 14 | 23,240,000 | 25,800,000 | 2,560,000 |
| 15 | 80,230,000 | 81,420,000 | 1,190,000 |
| 16 | 66,730,000 | 70,880,000 | 4,150,000 |
| 17 | 35,300,000 | 36,480,000 | 1,180,000 |
| 17 | 38,360,000 | 39,210,000 | 850,000 |
| 17 | 41,020,000 | 42,160,000 | 1,140,000 |
| 19 | 27,190,000 | 28,140,000 | 950,000 |
| 19 | 33,780,000 | 35,400,000 | 1,620,000 |
| 19 | 42,680,000 | 44,010,000 | 1,330,000 |
| 20 | 13,670,000 | 14,460,000 | 790,000 |
| 20 | 30,480,000 | 31,620,000 | 1,140,000 |
| 20 | 36,560,000 | 37,640,000 | 1,080,000 |
| 20 | 56,890,000 | 58,010,000 | 1,120,000 |
| 20 | 70,600,000 | 71,890,000 | 1,290,000 |
| 21 | 8,725 | 1,916,000 | 1,907,275 |
| 21 | 64,790,000 | 65,890,000 | 1,100,000 |
| 22 | 15,320,000 | 16,220,000 | 900,000 |
| 22 | 16,600,000 | 17,850,000 | 1,250,000 |
| 22 | 34,220,000 | 34,840,000 | 620,000 |
| 22 | 43,540,000 | 43,880,000 | 340,000 |
| 23 | 14,910 | 1,253,000 | 1,238,090 |
| 23 | 36,560,000 | 37,640,000 | 1,080,000 |
| 24 | 42,930,000 | 44,750,000 | 1,820,000 |
| 24 | 61,530,000 | 61,880,000 | 350,000 |
| 26 | 1,984,000 | 3,214,000 | 1,230,000 |
| 26 | 15,670,000 | 16,640,000 | 970,000 |
| 26 | 21,270,000 | 23,010,000 | 1,740,000 |
| 26 | 41,730,000 | 42,340,000 | 610,000 |
| 27 | 4,845,000 | 6,405,000 | 1,560,000 |
| 29 | 38,730,000 | 39,820,000 | 1,090,000 |

^1^ BTA: *Bos taurus* autosome.
