# Supplementary material for: Autozygosity islands and ROH patterns in Nellore lineages: evidence of selection for functionally important traits
Source: BMC Genomics. 2018 Sep 17;19:680. doi: 10.1186/s12864-018-5060-8 (PMC6142381; doi:10.1186/s12864-018-5060-8)
Supplement: Supplementary file 2 — Autozygosity islands within the Nellore lineages by chromosome: Karvadi (red), Golias (Black), Godhavari (Green), Taj Mahal (blue), Akasamu (purple), and Nagpur (yellow). (PDF 31 kb) [file 12864_2018_5060_MOESM2_ESM.pdf]

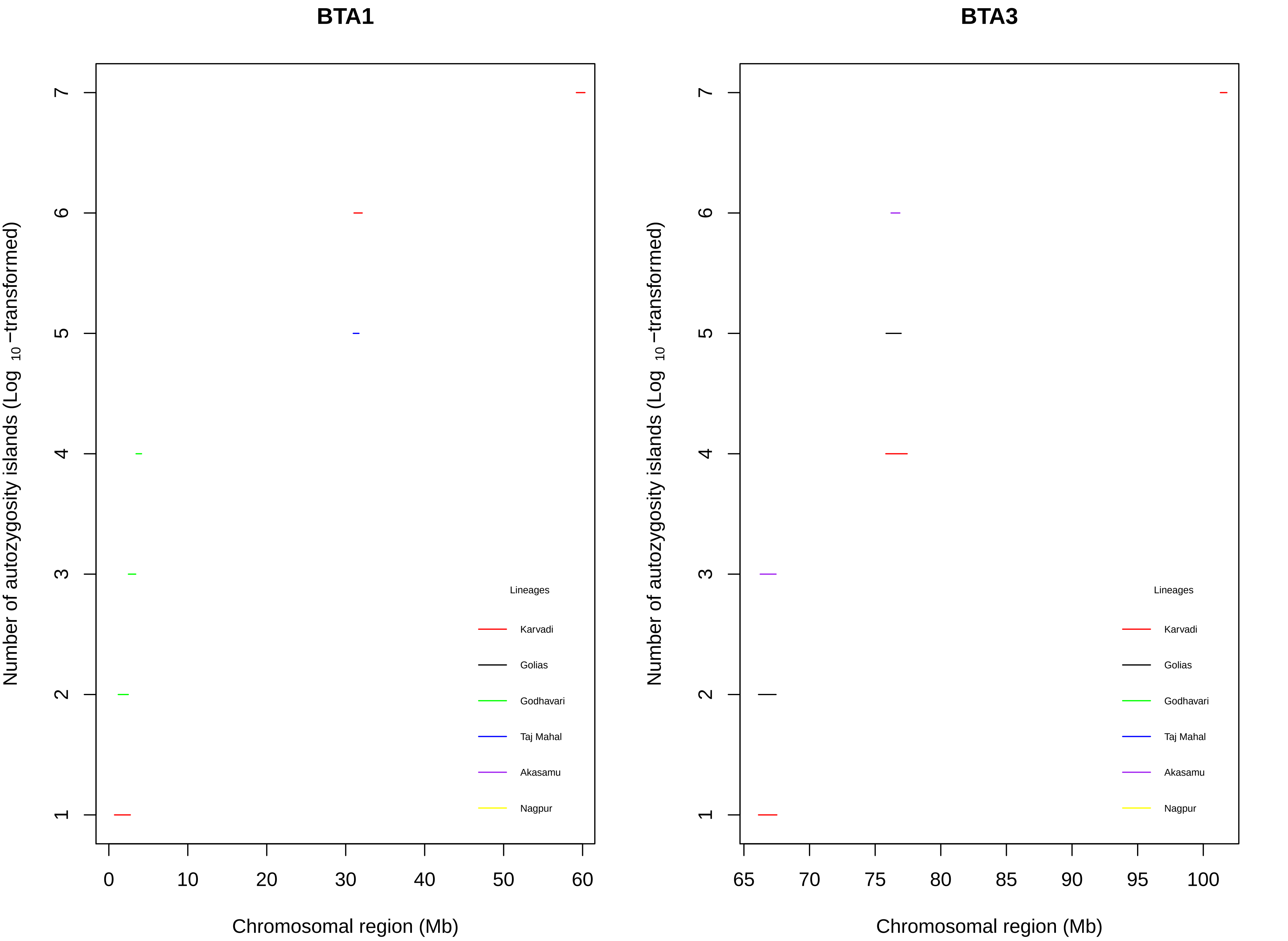
BTA1
BTA3
Number of autozygosity islands (Log 10 −transformed)
Chromosomal region (Mb)
Lineages
Karvadi
Golias
Godhavari
Taj Mahal
Akasamu
Nagpur
1
2
3
4
5
6
7
0
10
20
30
40
50
60
65
70
75
80
85
90
95
100

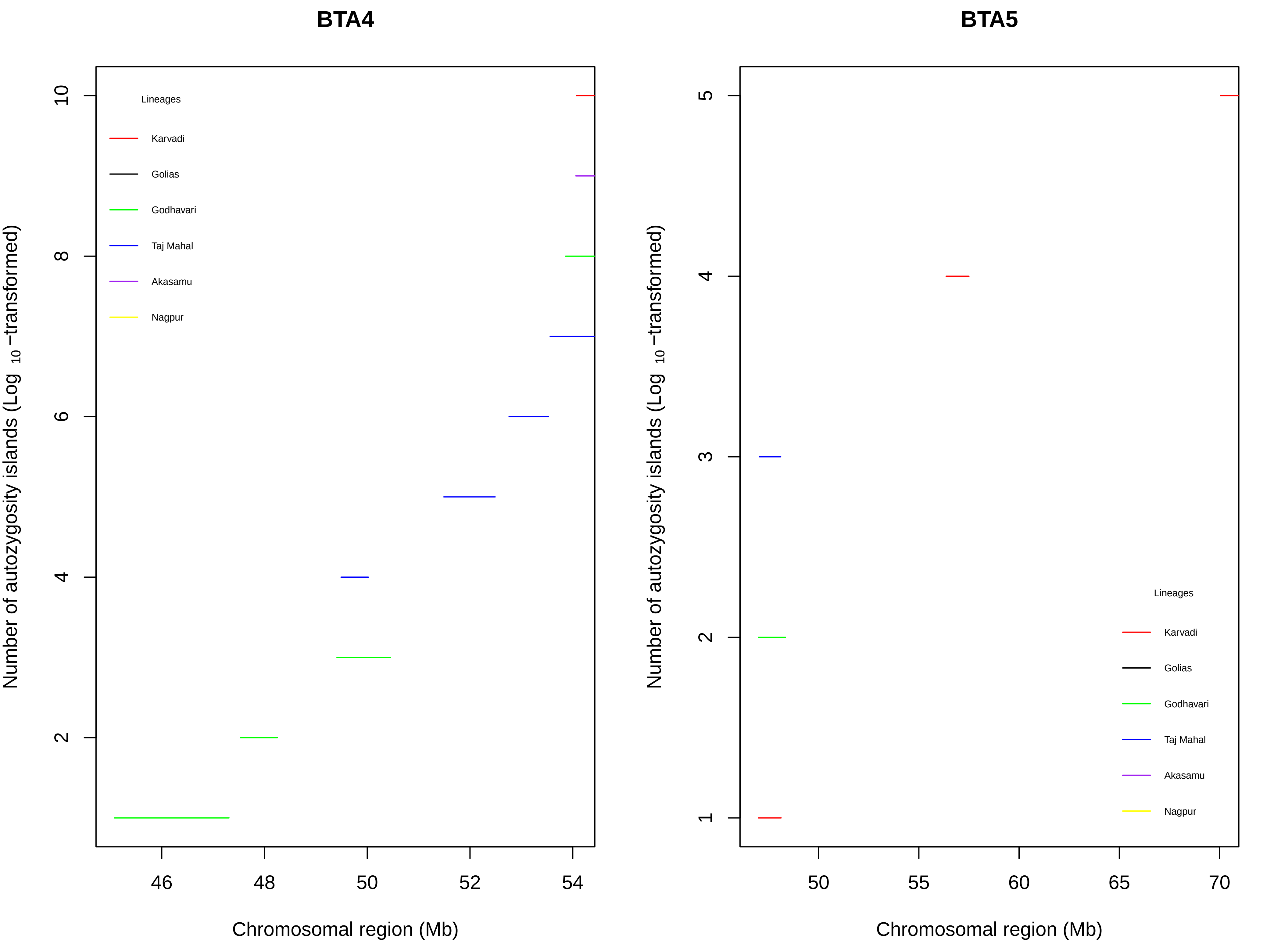
BTA4
BTA5
Lineages
Karvadi
Golias
Godhavari
Taj Mahal
Akasamu
Nagpur
Number of autozygosity islands (Log 10 −transformed)
Chromosomal region (Mb)
46
48
50
52
54
2
4
6
8
10
50
55
60
65
70
1
2
3
4
5

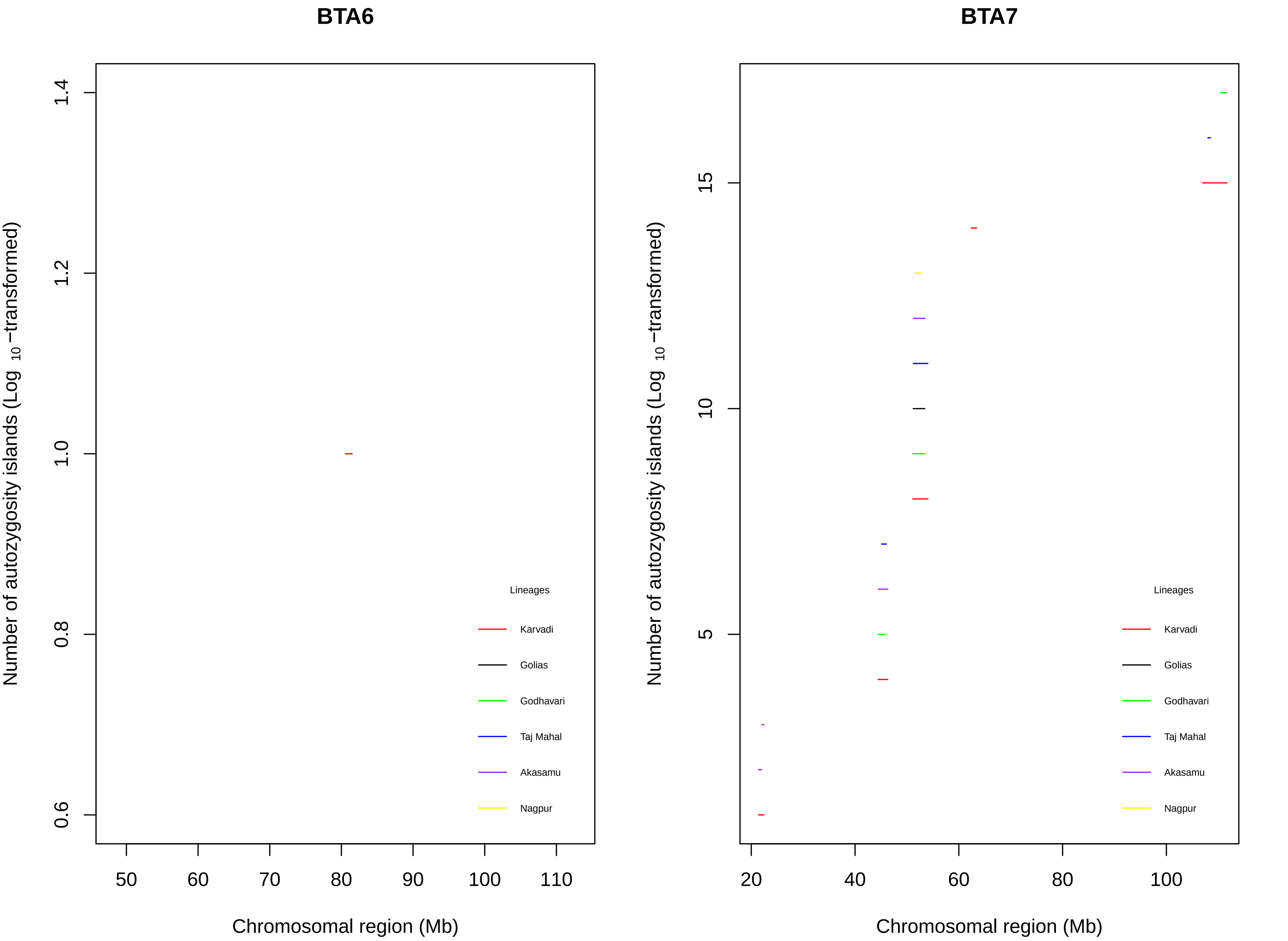

BTA6
BTA7
Number of autozygosity islands (Log 10 −transformed)
0.6
0.8
1.0
1.2
1.4
5
10
15
Lineages
Karvadi
Golias
Godhavari
Taj Mahal
Akasamu
Nagpur
50
60
70
80
90
100
110
20
40
60
80
100
Chromosomal region (Mb)

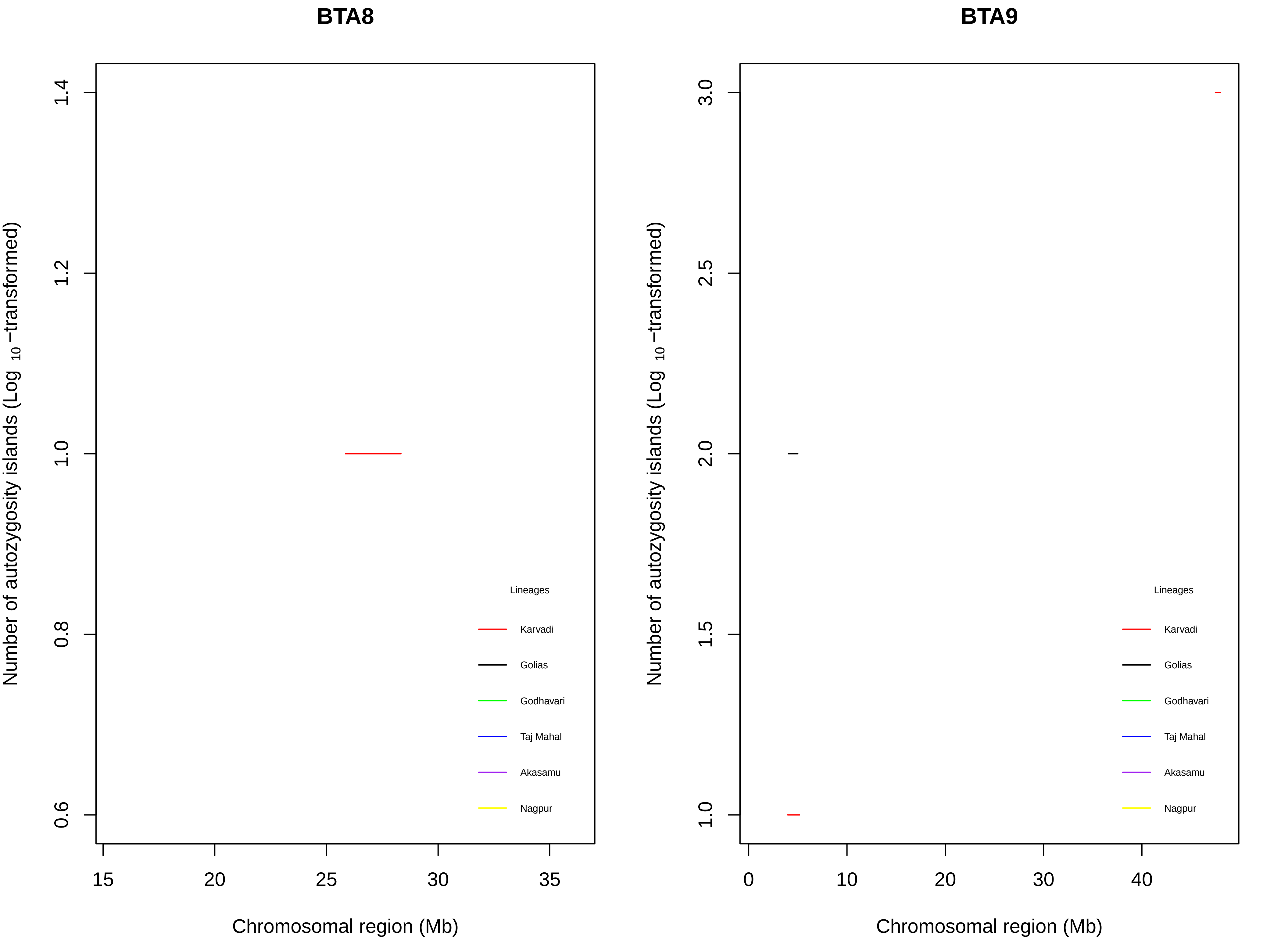
BTA8
BTA9
Number of autozygosity islands (Log 10 −transformed)
Chromosomal region (Mb)
Lineages
Karvadi
Golias
Godhavari
Taj Mahal
Akasamu
Nagpur
0.6
0.8
1.0
1.2
1.4
15
20
25
30
35
1.0
1.5
2.0
2.5
3.0
0
10
20
30
40

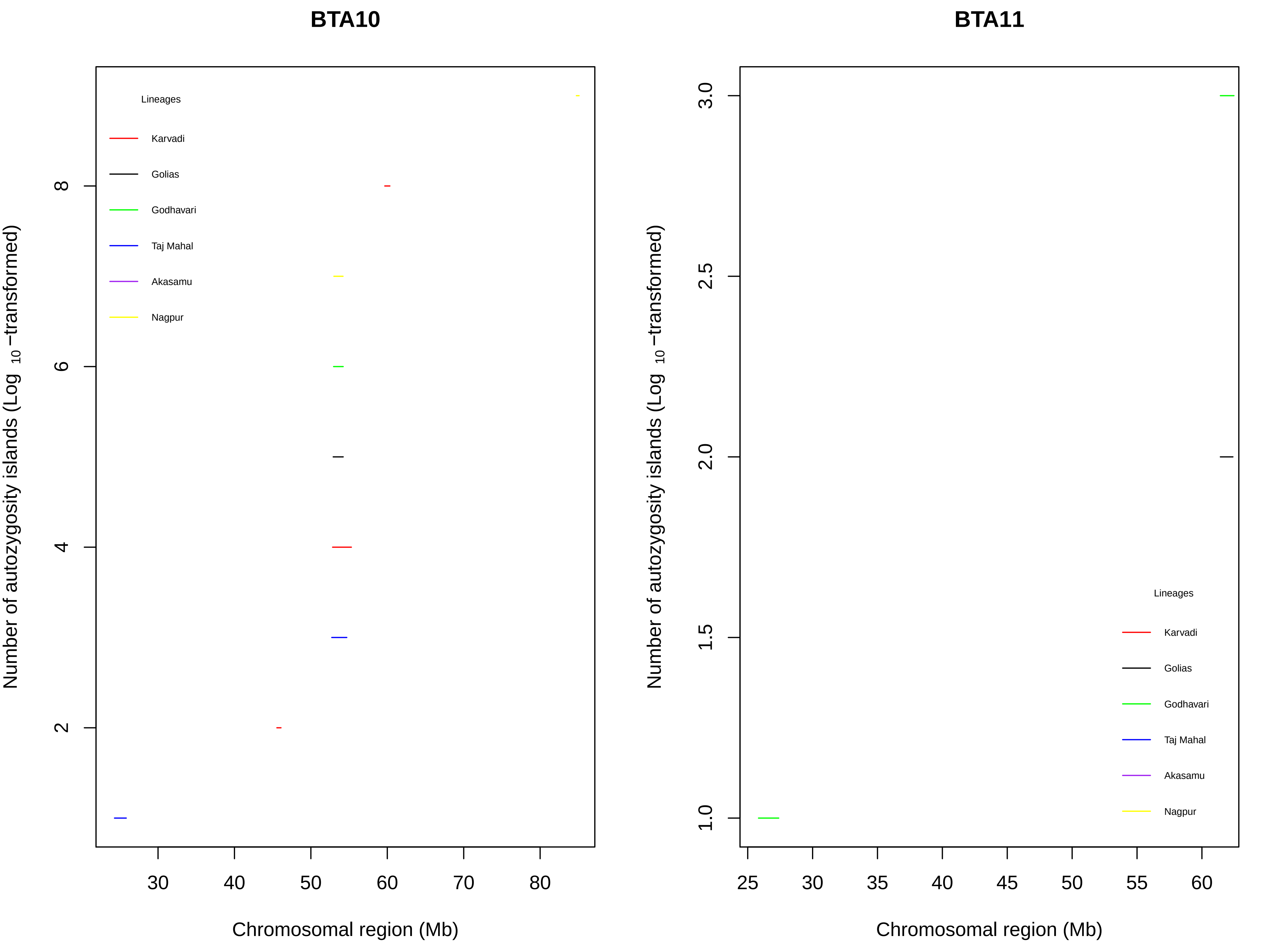

BTA10
BTA11
Lineages
Karvadi
Golias
Godhavari
Taj Mahal
Akasamu
Nagpur
Number of autozygosity islands (Log 10 −transformed)
Chromosomal region (Mb)

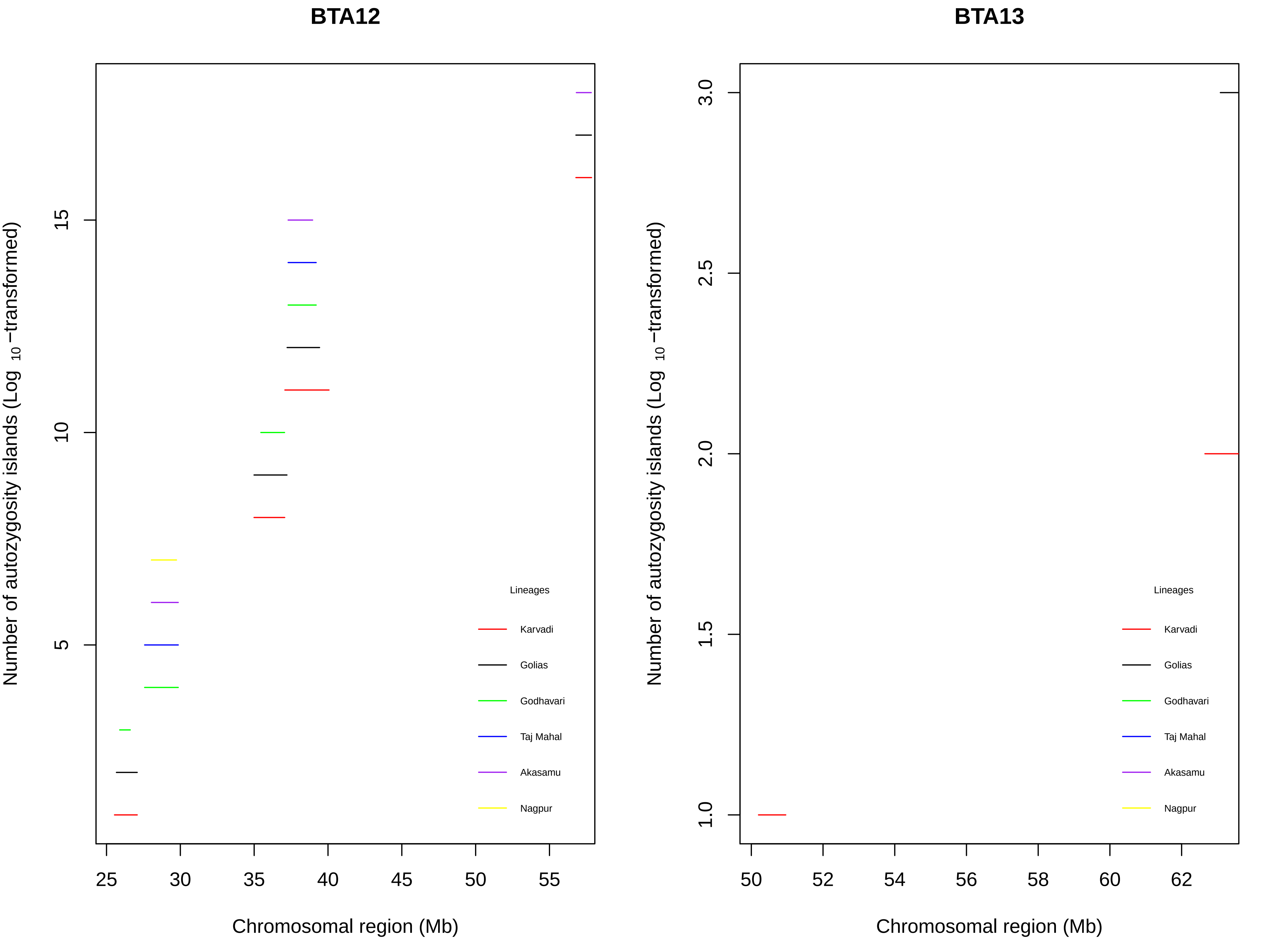
BTA12
BTA13
Number of autozygosity islands (Log 10 −transformed)
Chromosomal region (Mb)
Lineages
Karvadi
Golias
Godhavari
Taj Mahal
Akasamu
Nagpur
5
10
15
25
30
35
40
45
50
55
1.0
1.5
2.0
2.5
3.0
52
54
56
58
60
62

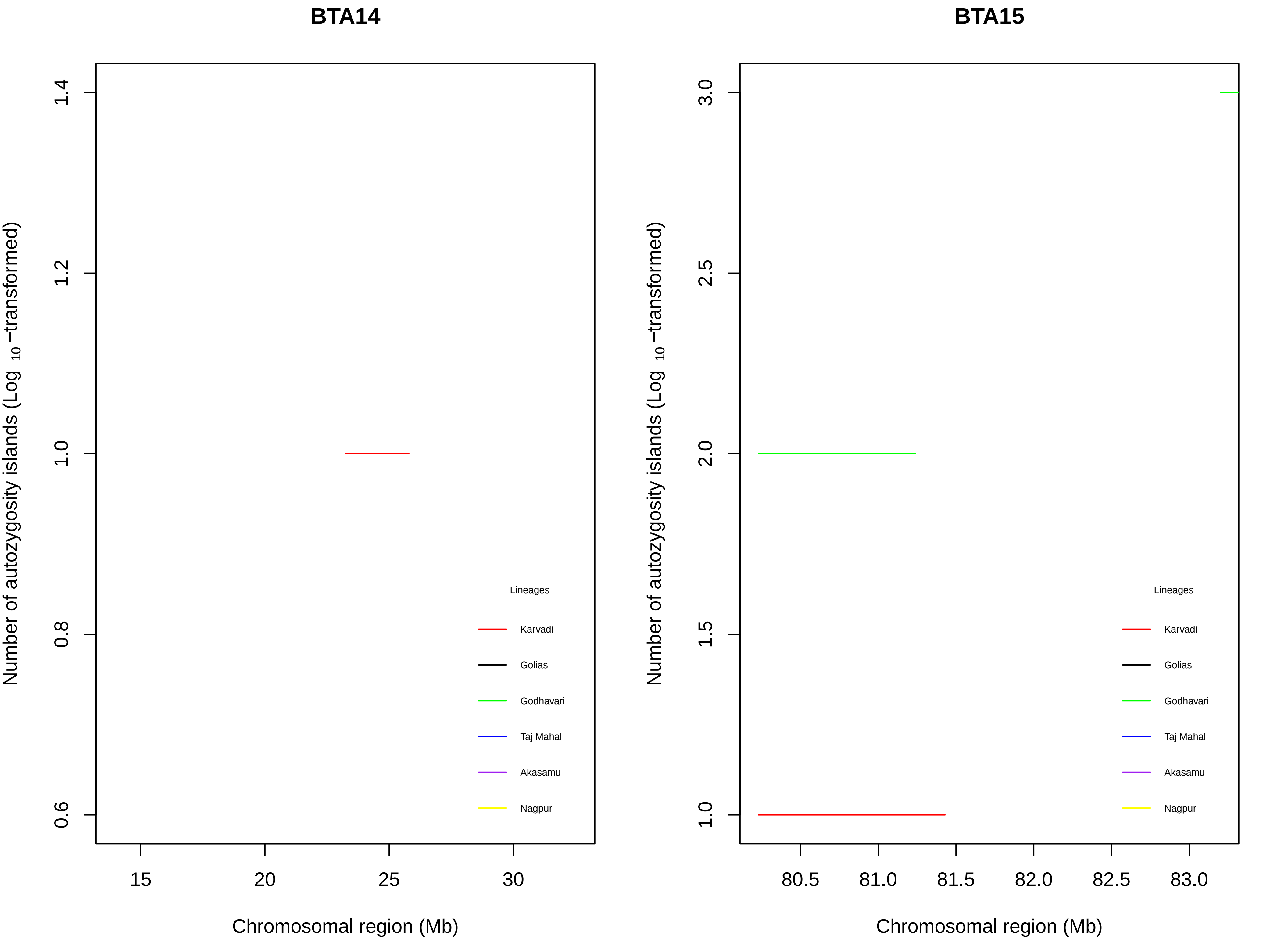
BTA14
BTA15
Number of autozygosity islands (Log 10 −transformed)
Chromosomal region (Mb)
Lineages
Karvadi
Golias
Godhavari
Taj Mahal
Akasamu
Nagpur
0.6
0.8
1.0
1.2
1.4
15
20
25
30
1.0
1.5
2.0
2.5
3.0
80.5
81.0
81.5
82.0
82.5
83.0

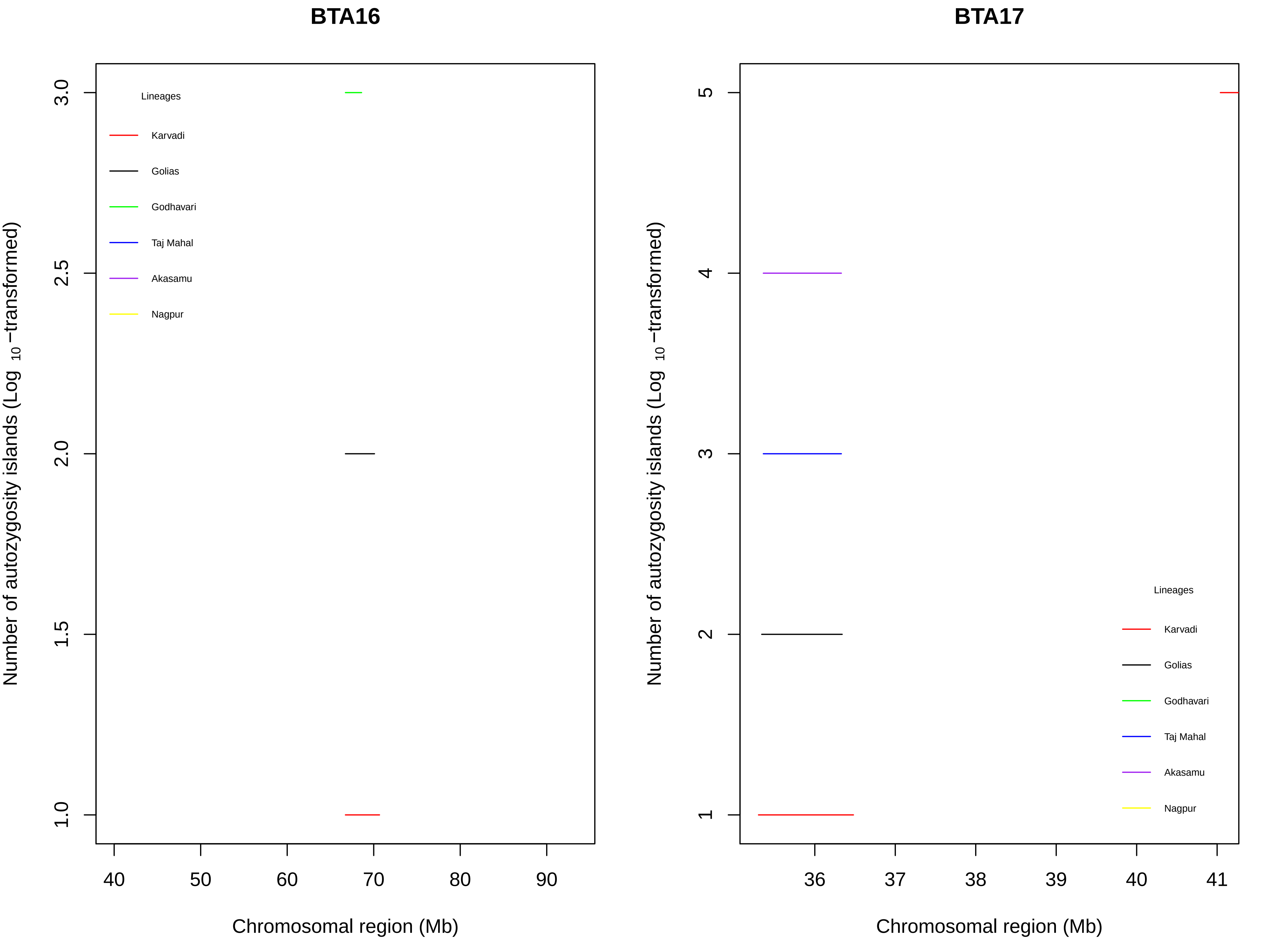

BTA16
BTA17
Number of autozygosity islands (Log 10 −transformed)
Chromosomal region (Mb)
Lineages
Karvadi
Golias
Godhavari
Taj Mahal
Akasamu
Nagpur
3.0
2.5
2.0
1.5
1.0
40
50
60
70
80
90
5
4
3
2
1
36
37
38
39
40
41

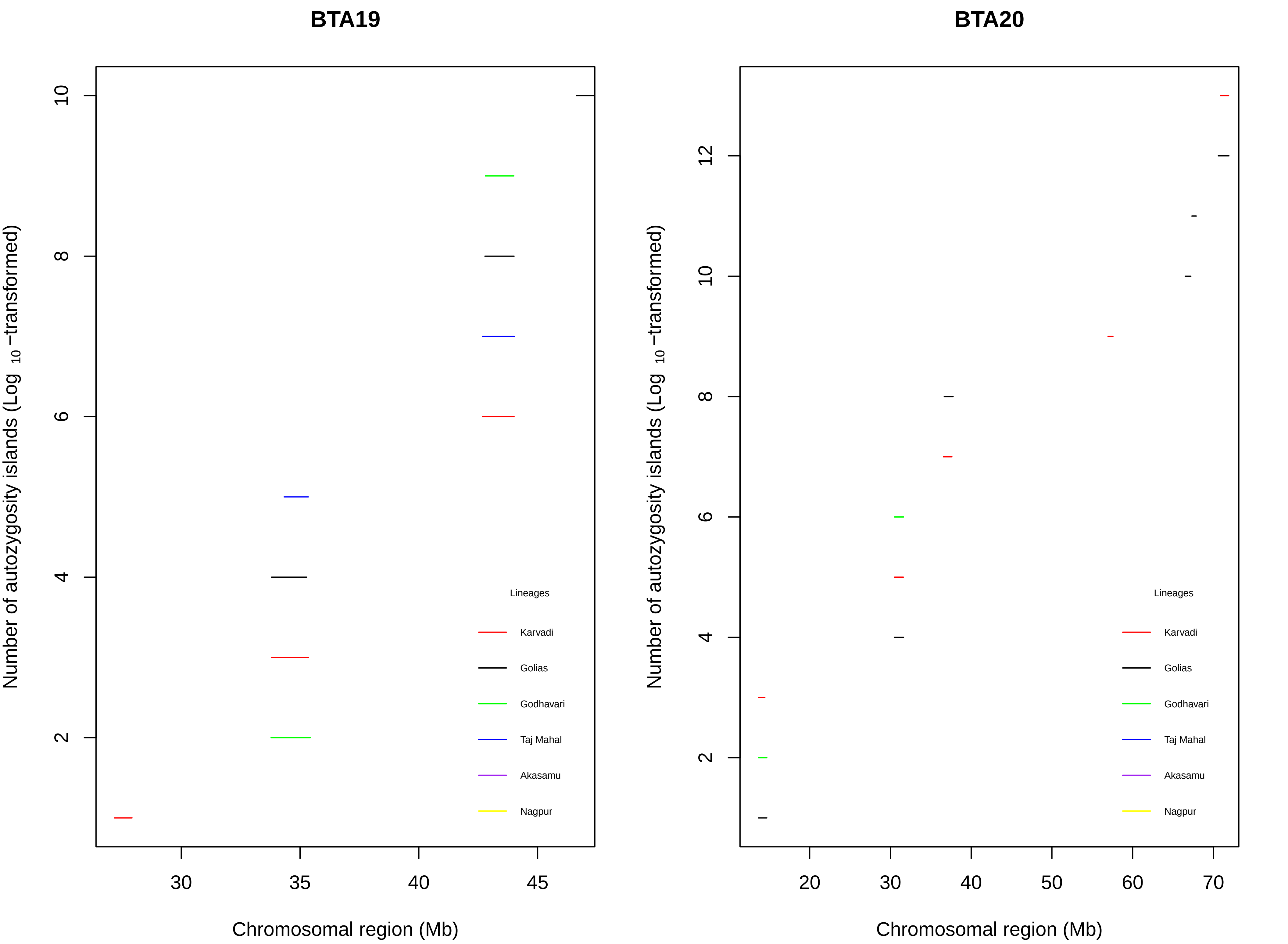

BTA19
BTA20
Number of autozygosity islands (Log 10 −transformed)
Chromosomal region (Mb)
Lineages
Karvadi
Golias
Godhavari
Taj Mahal
Akasamu
Nagpur

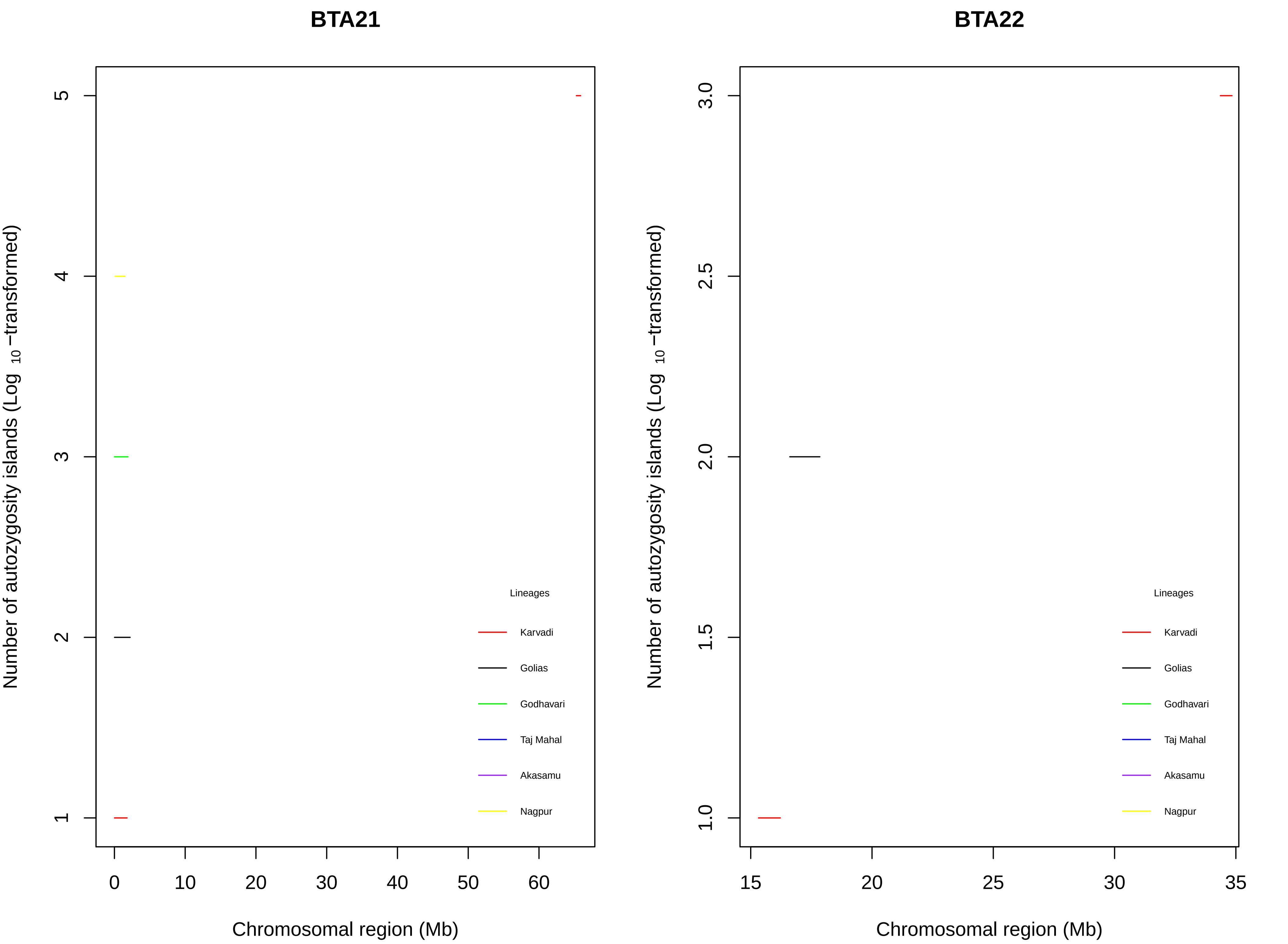
BTA21
BTA22
Number of autozygosity islands (Log 10 −transformed)
Chromosomal region (Mb)
Lineages
Karvadi
Golias
Godhavari
Taj Mahal
Akasamu
Nagpur
1
2
3
4
5
0
10
20
30
40
50
60
1.0
1.5
2.0
2.5
3.0
15
20
25
30
35

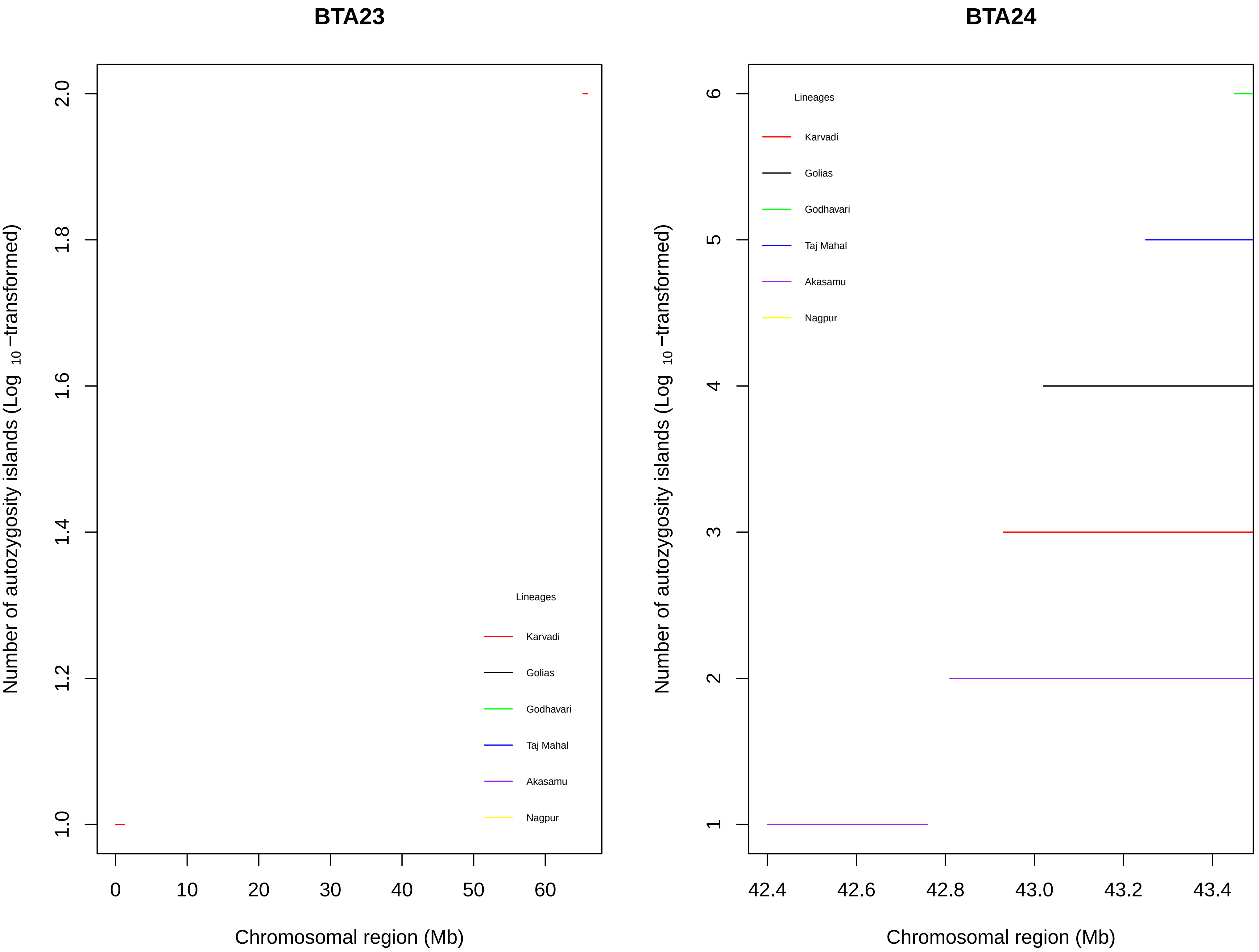

BTA23
BTA24
Number of autozygosity islands (Log 10 −transformed)
Chromosomal region (Mb)
Lineages
Karvadi
Golias
Godhavari
Taj Mahal
Akasamu
Nagpur
2.0
1.8
1.6
1.4
1.2
1.0
0
10
20
30
40
50
60
6
5
4
3
2
1
42.4
42.6
42.8
43.0
43.2
43.4

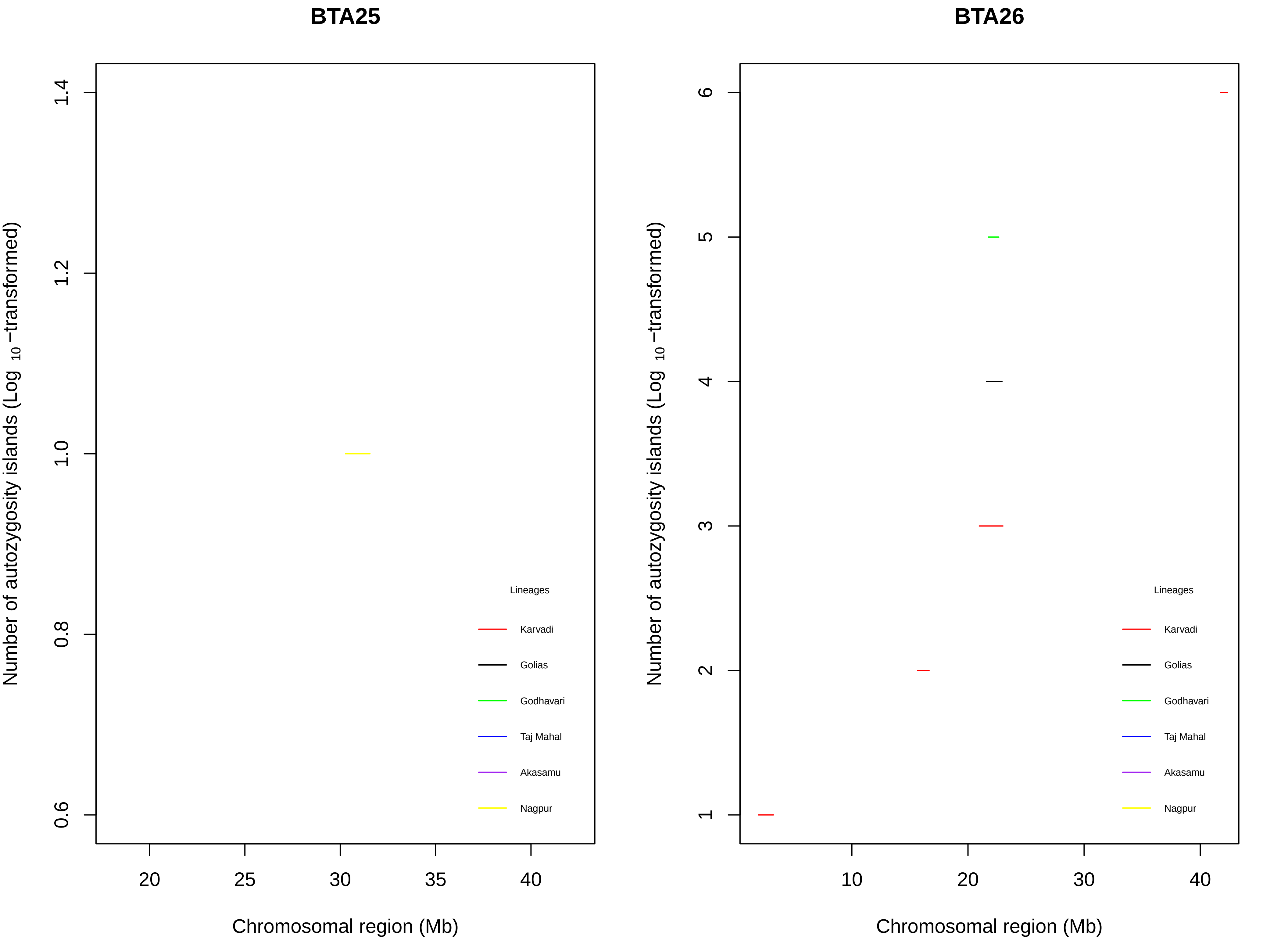
BTA25
BTA26
Number of autozygosity islands (Log 10 −transformed)
1.4
1.2
1.0
0.8
0.6
6
5
4
3
2
1
20
25
30
35
40
10
20
30
40
Chromosomal region (Mb)
Lineages
Karvadi
Golias
Godhavari
Taj Mahal
Akasamu
Nagpur

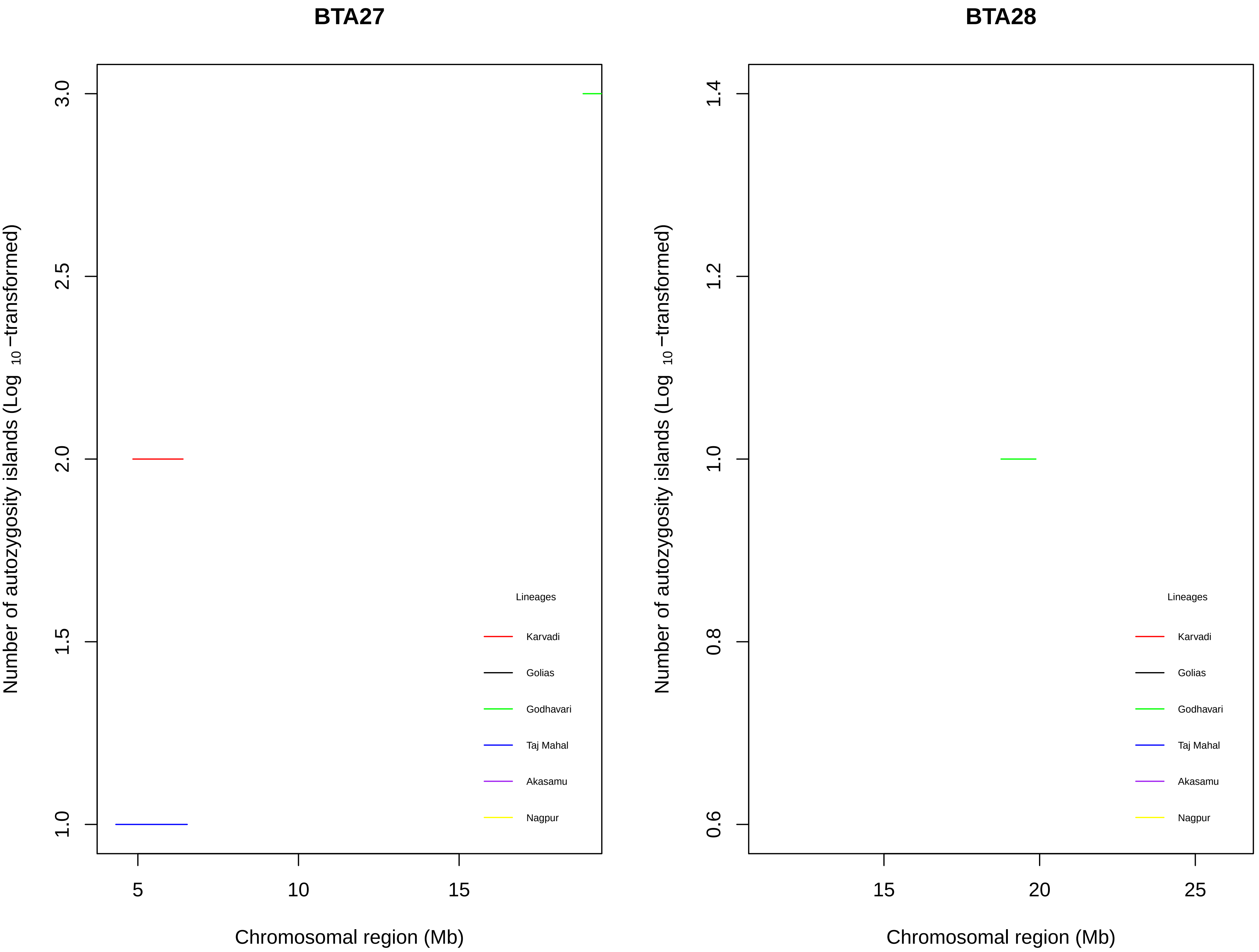
BTA27
BTA28
Number of autozygosity islands (Log 10 −transformed)
Chromosomal region (Mb)
Lineages
Karvadi
Golias
Godhavari
Taj Mahal
Akasamu
Nagpur
3.0
2.5
2.0
1.5
1.0
1.4
1.2
1.0
0.8
0.6
5
10
15
20
25

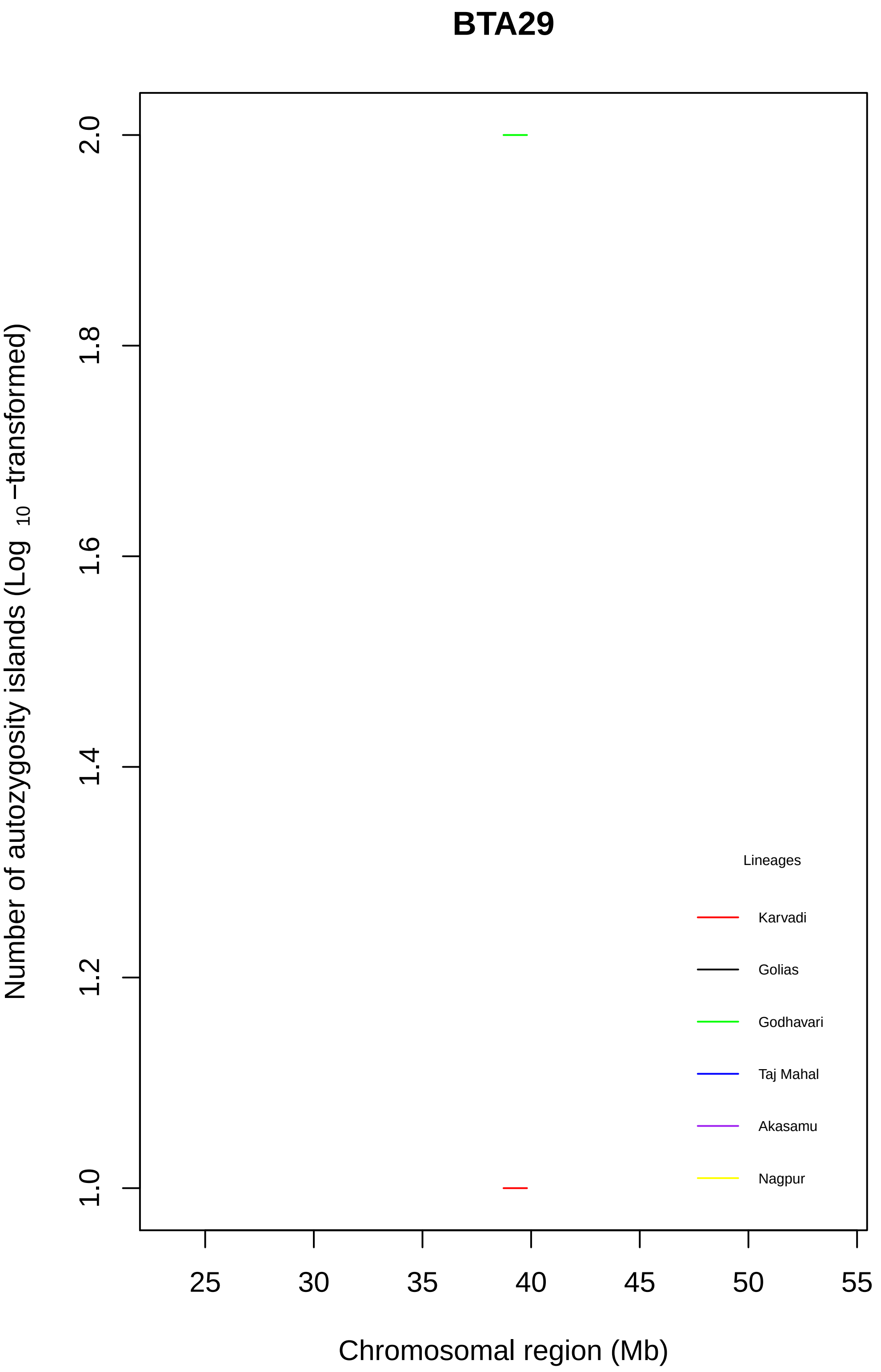

BTA29
Number of autozygosity islands (Log 10 −transformed)
2.0
1.8
1.6
1.4
1.2
1.0
25
30
35
40
45
50
55
Chromosomal region (Mb)
Lineages
Karvadi
Golias
Godhavari
Taj Mahal
Akasamu
Nagpur
