# Supplementary material for: Autozygosity islands and ROH patterns in Nellore lineages: evidence of selection for functionally important traits
Source: BMC Genomics. 2018 Sep 17;19:680. doi: 10.1186/s12864-018-5060-8 (PMC6142381; doi:10.1186/s12864-018-5060-8)
Supplement: Supplementary file 3 — Overlapping autozygosity islands within the Nellore lineages. (DOCX 28 kb) [file 12864_2018_5060_MOESM3_ESM.docx]

| Additional 3: Overlapping autozygosity islands within the Nellore lineages | | | | |
| --- | --- | --- | --- | --- |
| **BTA^1^** | **Start (bp)** | **End (bp)** | **Length (bp)** | **Lineages** |
| 1 | 1,185,000 | 2,466,000 | 1,281,001 | Karvadi, Godhavari |
| 1 | 2,472,000 | 2,725,000 | 253,001 | Karvadi, Godhavari |
| 1 | 31,050,000 | 31,680,000 | 630,001 | Taj Mahal, Karvadi |
| 3 | 66,110,000 | 66,239,999 | 130,000 | Karvadi, Golias |
| 3 | 66,240,000 | 67,450,000 | 1,210,001 | Karvadi, Golias, Akasamu |
| 3 | 75,830,000 | 76,209,999 | 380,000 | Karvadi, Golias |
| 3 | 76,210,000 | 76,880,000 | 670,001 | Karvadi, Golias, Akasamu |
| 3 | 76,880,001 | 76,980,000 | 100,000 | Karvadi, Golias |
| 4 | 49,490,000 | 50,020,000 | 530,001 | Godhavari, Taj Mahal |
| 4 | 53,860,000 | 54,059,999 | 200,000 | Taj Mahal, Godhavari |
| 4 | 54,060,000 | 54,069,999 | 10,000 | Taj Mahal, Godhavari, Akasamu |
| 4 | 54,070,000 | 55,690,000 | 1,620,001 | Taj Mahal, Godhavari, Akasamu, Karvadi |
| 4 | 55,690,001 | 55,800,000 | 110,000 | Taj Mahal, Godhavari, Karvadi |
| 4 | 55,800,001 | 55,820,000 | 20,000 | Taj Mahal, Godhavari |
| 5 | 47,000,000 | 47,049,999 | 50,000 | Godhavari, Karvadi |
| 5 | 47,050,000 | 48,110,000 | 1,060,001 | Godhavari, Karvadi, Taj Mahal |
| 5 | 48,110,001 | 48,130,000 | 20,000 | Godhavari, Karvadi |
| 7 | 21,410,000 | 21,990,000 | 580,001 | Karvadi, Akasamu |
| 7 | 22,020,000 | 22,430,000 | 410,001 | Karvadi, Akasamu |
| 7 | 44,470,000 | 44,489,999 | 20,000 | Karvadi, Godhavari |
| 7 | 44,490,000 | 45,119,999 | 630,000 | Karvadi, Godhavari, Akasamu |
| 7 | 45,120,000 | 45,830,000 | 710,001 | Karvadi, Godhavari, Akasamu, Taj Mahal |
| 7 | 45,830,001 | 46,050,000 | 220,000 | Karvadi, Akasamu, Taj Mahal |
| 7 | 46,050,001 | 46,300,000 | 250,000 | Karvadi, Akasamu |
| 7 | 51,140,000 | 51,209,999 | 70,000 | Karvadi, Godhavari |
| 7 | 51,210,000 | 51,229,999 | 20,000 | Karvadi, Godhavari, Golias |
| 7 | 51,230,000 | 51,249,999 | 20,000 | Karvadi, Godhavari, Golias, Taj Mahal |
| 7 | 51,250,000 | 51,609,999 | 360,000 | Karvadi, Godhavari, Golias, Taj Mahal, Akasamu |
| 7 | 51,610,000 | 52,930,000 | 1,320,001 | Karvadi, Godhavari, Golias, Taj Mahal, Akasamu, Nagpur |
| 7 | 52,930,001 | 53,440,000 | 510,000 | Karvadi, Godhavari, Golias, Taj Mahal, Akasamu |
| 7 | 53,440,001 | 53,490,000 | 50,000 | Karvadi, Godhavari, Taj Mahal, Akasamu, |
| 7 | 53,490,001 | 54,040,000 | 550,000 | Karvadi, Taj Mahal |
| 7 | 108,000,000 | 108,500,000 | 500,001 | Karvadi, Taj Mahal |
| 7 | 110,400,000 | 111,600,000 | 1,200,001 | Karvadi, Godhavari |
| 9 | 4,033,000 | 5,005,000 | 972,001 | Karvadi, Golias |
| 10 | 52,840,000 | 52,919,999 | 80,000 | Taj Mahal, Karvadi |
| 10 | 52,920,000 | 52,969,999 | 50,000 | Taj Mahal, Karvadi, Golias |
| 10 | 52,970,000 | 53,009,999 | 40,000 | Taj Mahal, Karvadi, Golias, Godhavari |
| 10 | 53,010,000 | 54,210,000 | 1,200,001 | Taj Mahal, Karvadi, Golias, Godhavari, Nagpur |
| 10 | 54,210,001 | 54,230,000 | 20,000 | Taj Mahal, Karvadi, Golias, Godhavari |
| 10 | 54,230,001 | 54,700,000 | 470,000 | Taj Mahal, Karvadi |
| 11 | 61,420,000 | 62,390,000 | 970,001 | Godhavari, Golias |
| 12 | 25,670,000 | 25,889,999 | 220,000 | Karvadi, Golias |
| 12 | 25,890,000 | 26,610,000 | 720,001 | Karvadi, Godhavari |
| 12 | 26,610,001 | 27,080,000 | 470,000 | Karvadi, Golias |
| 12 | 27,580,000 | 28,039,999 | 460,000 | Godhavari, Taj Mahal |
| 12 | 28,040,000 | 29,740,000 | 1,700,001 | Godhavari, Nagpur |
| 12 | 29,740,001 | 29,860,000 | 120,000 | Godhavari, Akasamu |
| 12 | 34,990,000 | 35,449,999 | 460,000 | Golias, Karvadi |
| 12 | 35,450,000 | 37,060,000 | 1,610,001 | Golias, Karvadi, Godhavari |
| 12 | 37,060,001 | 37,070,000 | 10,000 | Golias, Karvadi |
| 12 | 37,080,000 | 37,220,000 | 140,001 | Golias, Karvadi |
| 12 | 37,230,000 | 37,299,999 | 70,000 | Karvadi, Golias |
| 12 | 37,300,000 | 38,960,000 | 1,660,001 | Karvadi, Akasamu |
| 12 | 38,960,001 | 39,200,000 | 240,000 | Karvadi, Taj Mahal |
| 12 | 39,200,001 | 39,430,000 | 230,000 | Karvadi, Golias |
| 12 | 56,790,000 | 56,819,999 | 30,000 | Karvadi, Golias |
| 12 | 56,820,000 | 57,830,000 | 1,010,001 | Karvadi, Golias, Akasamu |
| 12 | 57,830,001 | 57,840,000 | 10,000 | Karvadi, Golias |
| 13 | 63,080,000 | 64,510,000 | 1,430,001 | Karvadi, Golias |
| 15 | 80,230,000 | 81,240,000 | 1,010,001 | Karvadi, Godhavari |
| 16 | 66,730,000 | 68,600,000 | 1,870,001 | Karvadi, Godhavari |
| 16 | 68,600,001 | 70,090,000 | 1,490,000 | Karvadi, Golias |
| 17 | 35,340,000 | 35,359,999 | 20,000 | Karvadi, Golias |
| 17 | 35,360,000 | 36,330,000 | 970,001 | Karvadi, Golias, Taj Mahal, Akasamu |
| 17 | 36,330,001 | 36,340,000 | 10,000 | Karvadi, Golias |
| 19 | 33,800,000 | 34,329,999 | 530,000 | Godhavari, Karvadi, Golias |
| 19 | 34,330,000 | 35,280,000 | 950,001 | Godhavari, Karvadi, Golias, Taj Mahal |
| 19 | 35,280,001 | 35,350,000 | 70,000 | Godhavari, Karvadi, Taj Mahal |
| 19 | 42,680,000 | 42,779,999 | 100,000 | Taj Mahal, Karvadi |
| 19 | 42,780,000 | 42,799,999 | 20,000 | Taj Mahal, Karvadi, Golias |
| 19 | 42,800,000 | 44,000,000 | 1,200,001 | Taj Mahal, Karvadi, Golias, Godhavari |
| 19 | 44,000,001 | 44,010,000 | 10,000 | Taj Mahal, Karvadi, Golias |
| 20 | 13,670,000 | 13,679,999 | 10,000 | Golias, Godhavari |
| 20 | 13,680,000 | 14,450,000 | 770,001 | Golias, Godhavari, Karvadi |
| 20 | 14,450,001 | 14700,000 | 250,000 | Golias, Godhavari |
| 20 | 30,510,000 | 31,600,000 | 1,090,001 | Golias, Godhavari, Karvadi |
| 20 | 31,600,001 | 31,630,000 | 30,000 | Golias, Godhavari |
| 20 | 36,660,000 | 37,620,000 | 960,001 | Karvadi, Golias |
| 20 | 70,860,000 | 71,890,000 | 1,030,001 | Golias, Karvadi |
| 21 | 8,725 | 112,599 | 103,875 | Golias, Godhavari, Karvadi |
| 21 | 112,600 | 1,483,000 | 1,370,401 | Golias, Godhavari, Karvadi, Nagpur |
| 21 | 1,483,001 | 1,790,000 | 307,000 | Golias, Godhavari, Karvadi |
| 21 | 1,790,001 | 1,916,000 | 126,000 | Golias, Godhavari |
| 24 | 42,930,000 | 43,019,999 | 90,000 | Akasamu, Karvadi |
| 24 | 43,020,000 | 43,249,999 | 230,000 | Akasamu, Karvadi, Golias |
| 24 | 43,250,000 | 43,449,999 | 200,000 | Akasamu, Karvadi, Golias, Taj Mahal |
| 24 | 43,450,000 | 43,930,000 | 480,001 | Akasamu, Karvadi, Golias, Taj Mahal, Godhavari |
| 24 | 43,930,001 | 44,030,000 | 100,000 | Akasamu, Karvadi, Golias, Taj Mahal |
| 24 | 44,030,001 | 44,080,000 | 50,000 | Akasamu, Karvadi |
| 26 | 21,590,000 | 21,749,999 | 160,000 | Karvadi, Golias |
| 26 | 21,750,000 | 22,660,000 | 910,001 | Karvadi, Godhavari |
| 26 | 22,660,001 | 22,930,000 | 270,000 | Karvadi, Golias |
| 27 | 4,845,000 | 6,405,000 | 1,560,001 | Taj Mahal, Karvadi |
| 29 | 38,730,000 | 39,810,000 | 1,080,001 | Karvadi, Godhavari |
| ^1^ BTA: *Bos taurus* autosome. | | | | |
