# Supplementary material for: Autozygosity islands and ROH patterns in Nellore lineages: evidence of selection for functionally important traits
Source: BMC Genomics. 2018 Sep 17;19:680. doi: 10.1186/s12864-018-5060-8 (PMC6142381; doi:10.1186/s12864-018-5060-8)
Supplement: Supplementary file 4 — Non-overlapping autozygosity islands within the Nellore lineages. (DOCX 26 kb) [file 12864_2018_5060_MOESM4_ESM.docx]

| Additional 4: Non-overlapping autozygosity islands within the Nellore lineages | | | | | |
| --- | --- | --- | --- | --- | --- |
| **BTA^1^** | **Start (bp)** | **End (bp)** | **Length (bp)** | **Lineage** | **Genes** |
| 1 | 59,210,000 | 60,300,000 | 1,090,001 | Karvadi | [*DRD3*](http://www.ensembl.org/bos_taurus/Gene/Summary?db=core;g=ENSBTAG00000002132)*,* [*TIGIT*](http://www.ensembl.org/bos_taurus/Gene/Summary?db=core;g=ENSBTAG00000011921)*,* [*ZBTB20*](http://www.ensembl.org/bos_taurus/Gene/Summary?db=core;g=ENSBTAG00000011928)*,* |
| 3 | 101,300,000 | 101,800,000 | 500,001 | Karvadi | [*TESK2*](http://www.ensembl.org/bos_taurus/Gene/Summary?db=core;g=ENSBTAG00000011244)*,* [*TOE1*](http://www.ensembl.org/bos_taurus/Gene/Summary?db=core;g=ENSBTAG00000011243)*,* [*MUTYH*](http://www.ensembl.org/bos_taurus/Gene/Summary?db=core;g=ENSBTAG00000011242)*,* [*HPDL*](http://www.ensembl.org/bos_taurus/Gene/Summary?db=core;g=ENSBTAG00000011241)*,* [*ZSWIM5*](http://www.ensembl.org/bos_taurus/Gene/Summary?db=core;g=ENSBTAG00000012648)*,* [*UROD*](http://www.ensembl.org/bos_taurus/Gene/Summary?db=core;g=ENSBTAG00000012644)*,* [*HECTD3*](http://www.ensembl.org/bos_taurus/Gene/Summary?db=core;g=ENSBTAG00000019013)*,* [*EIF2B3*](http://www.ensembl.org/bos_taurus/Gene/Summary?db=core;g=ENSBTAG00000019010)*,* [*TCH2*](http://www.ensembl.org/bos_taurus/Gene/Summary?db=core;g=ENSBTAG00000024137) |
| 4 | 45,080,000 | 47,310,000 | 2,230,001 | Godhavari | [*RELN*](http://www.ensembl.org/bos_taurus/Gene/Summary?db=core;g=ENSBTAG00000003658)*,* [*ORC5*](http://www.ensembl.org/bos_taurus/Gene/Summary?db=core;g=ENSBTAG00000040058)*,* [*LHFPL3*](http://www.ensembl.org/bos_taurus/Gene/Summary?db=core;g=ENSBTAG00000000189)*,* [*KMT2E*](http://www.ensembl.org/bos_taurus/Gene/Summary?db=core;g=ENSBTAG00000009207)*,* [*SRPK2*](http://www.ensembl.org/bos_taurus/Gene/Summary?db=core;g=ENSBTAG00000002175)*,* [*PUS7*](http://www.ensembl.org/bos_taurus/Gene/Summary?db=core;g=ENSBTAG00000007743)*,* [*RINT1*](http://www.ensembl.org/bos_taurus/Gene/Summary?db=core;g=ENSBTAG00000008302) |
| 4 | 47,530,000 | 48,250,000 | 720,001 | Godhavari | [*NAMPT*](http://www.ensembl.org/bos_taurus/Gene/Summary?db=core;g=ENSBTAG00000015509) |
| 4 | 49,410,000 | 49,489,999 | 80,000 | Godhavari | [*LAMB4*](http://www.ensembl.org/bos_taurus/Gene/Summary?db=core;g=ENSBTAG00000033169) |
| 4 | 51,490,000 | 52,490,000 | 1,000,001 | Taj Mahal | [*ST7*](http://www.ensembl.org/bos_taurus/Gene/Summary?db=core;g=ENSBTAG00000010756)*,* [*CAPZA2*](http://www.ensembl.org/bos_taurus/Gene/Summary?db=core;g=ENSBTAG00000004072)*,* [*MET*](http://www.ensembl.org/bos_taurus/Gene/Summary?db=core;g=ENSBTAG00000006161)*,* [*CAV1*](http://www.ensembl.org/bos_taurus/Gene/Summary?db=core;g=ENSBTAG00000017869)*, CAV12, TES* |
| 4 | 52,760,000 | 53,530,000 | 770,001 | Taj Mahal | [*TFEC*](http://www.ensembl.org/bos_taurus/Gene/Summary?db=core;g=ENSBTAG00000014228) |
| 4 | 55,820,001 | 55,940,000 | 120,000 | Godhavari | [*LSMEM1*](http://www.ensembl.org/bos_taurus/Gene/Summary?db=core;g=ENSBTAG00000010555)*,* [*IFRD1*](http://www.ensembl.org/bos_taurus/Gene/Summary?db=core;g=ENSBTAG00000010549) |
| 5 | 56,360,000 | 57,500,000 | 1,140,001 | Karvadi | [*R3HDM2*](http://www.ensembl.org/bos_taurus/Gene/Summary?db=core;g=ENSBTAG00000018361)*,* [*STAC3*](http://www.ensembl.org/bos_taurus/Gene/Summary?db=core;g=ENSBTAG00000018358)*,* [*NDUFA4L2*](http://www.ensembl.org/bos_taurus/Gene/Summary?db=core;g=ENSBTAG00000031503)*,* [*SHMT2*](http://www.ensembl.org/bos_taurus/Gene/Summary?db=core;g=ENSBTAG00000031500)*,* [*NXPH4*](http://www.ensembl.org/bos_taurus/Gene/Summary?db=core;g=ENSBTAG00000047650)*,* [*LRP1*](http://www.ensembl.org/bos_taurus/Gene/Summary?db=core;g=ENSBTAG00000010830)*,* [*STAT6*](http://www.ensembl.org/bos_taurus/Gene/Summary?db=core;g=ENSBTAG00000006335)*,* [*NAB2*](http://www.ensembl.org/bos_taurus/Gene/Summary?db=core;g=ENSBTAG00000006324)*,* [*NEMP1*](http://www.ensembl.org/bos_taurus/Gene/Summary?db=core;g=ENSBTAG00000014659)*,* [*MYO1A*](http://www.ensembl.org/bos_taurus/Gene/Summary?db=core;g=ENSBTAG00000014655)*,* [*TAC3*](http://www.ensembl.org/bos_taurus/Gene/Summary?db=core;g=ENSBTAG00000021807)*,* [*ZBTB39*](http://www.ensembl.org/bos_taurus/Gene/Summary?db=core;g=ENSBTAG00000038498)*, G*[*PR182*](http://www.ensembl.org/bos_taurus/Gene/Summary?db=core;g=ENSBTAG00000039157)*,* [*RDH16*](http://www.ensembl.org/bos_taurus/Gene/Summary?db=core;g=ENSBTAG00000001392)*,* [*SDR9C7*](http://www.ensembl.org/bos_taurus/Gene/Summary?db=core;g=ENSBTAG00000013443)*,* |
| 5 | 70,040,000 | 71,090,000 | 1,050,001 | Karvadi | [*TCP11L2*](http://www.ensembl.org/bos_taurus/Gene/Summary?db=core;g=ENSBTAG00000002127)*, P*[*OLR3B*](http://www.ensembl.org/bos_taurus/Gene/Summary?db=core;g=ENSBTAG00000004781)*,* [*RFX4*](http://www.ensembl.org/bos_taurus/Gene/Summary?db=core;g=ENSBTAG00000025837)*,* [*RIC8B*](http://www.ensembl.org/bos_taurus/Gene/Summary?db=core;g=ENSBTAG00000000147)*,* [*TMEM263*](http://www.ensembl.org/bos_taurus/Gene/Summary?db=core;g=ENSBTAG00000014411)*,* [*MTERF2*](http://www.ensembl.org/bos_taurus/Gene/Summary?db=core;g=ENSBTAG00000010144)*,* [*CRY1*](http://www.ensembl.org/bos_taurus/Gene/Summary?db=core;g=ENSBTAG00000010149)*,* [*BTBD11*](http://www.ensembl.org/bos_taurus/Gene/Summary?db=core;g=ENSBTAG00000027064) |
| 6 | 80,560,000 | 81,500,000 | 940,001 | Karvadi | *-* |
| 7 | 62,370,000 | 63,440,000 | 1,070,001 | Karvadi | [*SH3TC2*](http://www.ensembl.org/bos_taurus/Gene/Summary?db=core;g=ENSBTAG00000017151)*,* [*ABLIM3*](http://www.ensembl.org/bos_taurus/Gene/Summary?db=core;g=ENSBTAG00000004896)*,* [*AFAP1L1*](http://www.ensembl.org/bos_taurus/Gene/Summary?db=core;g=ENSBTAG00000019948)*,* [*GRPEL2*](http://www.ensembl.org/bos_taurus/Gene/Summary?db=core;g=ENSBTAG00000019950)*,* [*PCYOX1L*](http://www.ensembl.org/bos_taurus/Gene/Summary?db=core;g=ENSBTAG00000019951)*,* [*IL17B*](http://www.ensembl.org/bos_taurus/Gene/Summary?db=core;g=ENSBTAG00000010578)*,* [*CSNK1A1*](http://www.ensembl.org/bos_taurus/Gene/Summary?db=core;g=ENSBTAG00000005326)*,* [*ARHGEF37*](http://www.ensembl.org/bos_taurus/Gene/Summary?db=core;g=ENSBTAG00000015419)*,* [*PPARGC1B*](http://www.ensembl.org/bos_taurus/Gene/Summary?db=core;g=ENSBTAG00000012943)*,* [*PDE6A*](http://www.ensembl.org/bos_taurus/Gene/Summary?db=core;g=ENSBTAG00000012945)*,* [*SLC26A2*](http://www.ensembl.org/bos_taurus/Gene/Summary?db=core;g=ENSBTAG00000014615)*,* [*HMGXB3*](http://www.ensembl.org/bos_taurus/Gene/Summary?db=core;g=ENSBTAG00000012768)*,* [*CSF1R*](http://www.ensembl.org/bos_taurus/Gene/Summary?db=core;g=ENSBTAG00000012771) |
| 8 | 25,850,000 | 28,340,000 | 2,490,001 | Karvadi | [*SH3GL2*](http://www.ensembl.org/bos_taurus/Gene/Summary?db=core;g=ENSBTAG00000014103)*,* [*CNTLN*](http://www.ensembl.org/bos_taurus/Gene/Summary?db=core;g=ENSBTAG00000001847)*,* [*BNC2*](http://www.ensembl.org/bos_taurus/Gene/Summary?db=core;g=ENSBTAG00000003669) |
| 9 | 47,470,000 | 47,980,000 | 510,001 | Karvadi | *-* |
| 10 | 24,300,000 | 25,840,000 | 1,540,001 | Taj Mahal | [*TRAV17*](http://www.ensembl.org/bos_taurus/Gene/Summary?db=core;g=ENSBTAG00000022229)*, TRAV178* |
| 10 | 45,550,000 | 46,090,000 | 540,001 | Karvadi | [*ZNF609*](http://www.ensembl.org/bos_taurus/Gene/Summary?db=core;g=ENSBTAG00000015808)*,* [*TRIP4*](http://www.ensembl.org/bos_taurus/Gene/Summary?db=core;g=ENSBTAG00000014493)*,* [*PCLAF*](http://www.ensembl.org/bos_taurus/Gene/Summary?db=core;g=ENSBTAG00000039462)*,* [*CSNK1G1*](http://www.ensembl.org/bos_taurus/Gene/Summary?db=core;g=ENSBTAG00000016823)*,* [*PPIB*](http://www.ensembl.org/bos_taurus/Gene/Summary?db=core;g=ENSBTAG00000016822)*,* [*SNX22*](http://www.ensembl.org/bos_taurus/Gene/Summary?db=core;g=ENSBTAG00000016821)*,* [*SNX1*](http://www.ensembl.org/bos_taurus/Gene/Summary?db=core;g=ENSBTAG00000002014)*,* [*FAM96A*](http://www.ensembl.org/bos_taurus/Gene/Summary?db=core;g=ENSBTAG00000002012)*,* [*DAPK2*](http://www.ensembl.org/bos_taurus/Gene/Summary?db=core;g=ENSBTAG00000011820) |
| 10 | 59,670,000 | 60,340,000 | 670,001 | Karvadi | [*SPPL2A*](http://www.ensembl.org/bos_taurus/Gene/Summary?db=core;g=ENSBTAG00000011831)*,* [*TRPM7*](http://www.ensembl.org/bos_taurus/Gene/Summary?db=core;g=ENSBTAG00000031165)*,* [*USP50*](http://www.ensembl.org/bos_taurus/Gene/Summary?db=core;g=ENSBTAG00000011929)*,* [*USP8*](http://www.ensembl.org/bos_taurus/Gene/Summary?db=core;g=ENSBTAG00000011916)*,* [*GABPB1*](http://www.ensembl.org/bos_taurus/Gene/Summary?db=core;g=ENSBTAG00000022801)*,* [*HDC*](http://www.ensembl.org/bos_taurus/Gene/Summary?db=core;g=ENSBTAG00000009911)*,* [*SLC27A2*](http://www.ensembl.org/bos_taurus/Gene/Summary?db=core;g=ENSBTAG00000004303) |
| 10 | 84,740,000 | 85,100,000 | 360,001 | Nagpur | [*DCAF4*](http://www.ensembl.org/bos_taurus/Gene/Summary?db=core;g=ENSBTAG00000008713)*,* [*ZFYVE1*](http://www.ensembl.org/bos_taurus/Gene/Summary?db=core;g=ENSBTAG00000011041)*,* [*RBM25*](http://www.ensembl.org/bos_taurus/Gene/Summary?db=core;g=ENSBTAG00000017177)*,* [*PSEN1*](http://www.ensembl.org/bos_taurus/Gene/Summary?db=core;g=ENSBTAG00000011757)*,* [*PAPLN*](http://www.ensembl.org/bos_taurus/Gene/Summary?db=core;g=ENSBTAG00000011680) |
| 11 | 25,830,000 | 27,390,000 | 1,560,001 | Godhavari | [*THADA*](http://www.ensembl.org/bos_taurus/Gene/Summary?db=core;g=ENSBTAG00000003555)*,* [*PLEKHH2*](http://www.ensembl.org/bos_taurus/Gene/Summary?db=core;g=ENSBTAG00000032521)*,* [*DYNC2LI1*](http://www.ensembl.org/bos_taurus/Gene/Summary?db=core;g=ENSBTAG00000013676)*,* [*ABCG5*](http://www.ensembl.org/bos_taurus/Gene/Summary?db=core;g=ENSBTAG00000016365)*,* [*ABCG8*](http://www.ensembl.org/bos_taurus/Gene/Summary?db=core;g=ENSBTAG00000016366)*,* [*LRPPRC*](http://www.ensembl.org/bos_taurus/Gene/Summary?db=core;g=ENSBTAG00000016368)*,* [*PPM1B*](http://www.ensembl.org/bos_taurus/Gene/Summary?db=core;g=ENSBTAG00000000223)*,* [*SLC3A1*](http://www.ensembl.org/bos_taurus/Gene/Summary?db=core;g=ENSBTAG00000017421)*,* [*PREPL*](http://www.ensembl.org/bos_taurus/Gene/Summary?db=core;g=ENSBTAG00000017429)*,* [*CAMKMT*](http://www.ensembl.org/bos_taurus/Gene/Summary?db=core;g=ENSBTAG00000032519)*,* [*SIX3*](http://www.ensembl.org/bos_taurus/Gene/Summary?db=core;g=ENSBTAG00000027017)*,* [*SIX2*](http://www.ensembl.org/bos_taurus/Gene/Summary?db=core;g=ENSBTAG00000004159) |
| 13 | 50,200,000 | 50,960,000 | 760,001 | Karvadi | *-* |
| 14 | 23,240,000 | 25,800,000 | 2,560,001 | Karvadi | [*NPBWR1*](http://www.ensembl.org/bos_taurus/Gene/Summary?db=core;g=ENSBTAG00000016159)*,* [*OPRK1*](http://www.ensembl.org/bos_taurus/Gene/Summary?db=core;g=ENSBTAG00000000914)*,* [*ATP6V1H*](http://www.ensembl.org/bos_taurus/Gene/Summary?db=core;g=ENSBTAG00000003450)*,* [*RGS20*](http://www.ensembl.org/bos_taurus/Gene/Summary?db=core;g=ENSBTAG00000003454)*,* [*TCEA1*](http://www.ensembl.org/bos_taurus/Gene/Summary?db=core;g=ENSBTAG00000003460)*,* [*LYPLA1*](http://www.ensembl.org/bos_taurus/Gene/Summary?db=core;g=ENSBTAG00000004243)*,* [*MRPL15*](http://www.ensembl.org/bos_taurus/Gene/Summary?db=core;g=ENSBTAG00000001174)*,* [*POLR2K*](http://www.ensembl.org/bos_taurus/Gene/Summary?db=core;g=ENSBTAG00000022539)*,* [*SOX17*](http://www.ensembl.org/bos_taurus/Gene/Summary?db=core;g=ENSBTAG00000005748)*,* [*RP1*](http://www.ensembl.org/bos_taurus/Gene/Summary?db=core;g=ENSBTAG00000011203)*,* [*XKR4*](http://www.ensembl.org/bos_taurus/Gene/Summary?db=core;g=ENSBTAG00000044050)*,* [*TMEM68*](http://www.ensembl.org/bos_taurus/Gene/Summary?db=core;g=ENSBTAG00000005893)*,* [*TGS1*](http://www.ensembl.org/bos_taurus/Gene/Summary?db=core;g=ENSBTAG00000005898)*,* [*LYN*](http://www.ensembl.org/bos_taurus/Gene/Summary?db=core;g=ENSBTAG00000020034)*,* [*RPS20*](http://www.ensembl.org/bos_taurus/Gene/Summary?db=core;g=ENSBTAG00000019147)*,* |
| 15 | 83,200,000 | 84,040,000 | 840,001 | Godhavari | [*LPXN*](http://www.ensembl.org/bos_taurus/Gene/Summary?db=core;g=ENSBTAG00000036260)*,* [*CNTF*](http://www.ensembl.org/bos_taurus/Gene/Summary?db=core;g=ENSBTAG00000003624)*,* [*GLYAT*](http://www.ensembl.org/bos_taurus/Gene/Summary?db=core;g=ENSBTAG00000038323)*,* [*GAT*](http://www.ensembl.org/bos_taurus/Gene/Summary?db=core;g=ENSBTAG00000012540)*,* [*GLYATL2*](http://www.ensembl.org/bos_taurus/Gene/Summary?db=core;g=ENSBTAG00000030847)*,* [*FAM111B*](http://www.ensembl.org/bos_taurus/Gene/Summary?db=core;g=ENSBTAG00000036016)*,* [*DTX4*](http://www.ensembl.org/bos_taurus/Gene/Summary?db=core;g=ENSBTAG00000004046)*,* [*MPEG1*](http://www.ensembl.org/bos_taurus/Gene/Summary?db=core;g=ENSBTAG00000030845)*,* [*OR5A1*](http://www.ensembl.org/bos_taurus/Gene/Summary?db=core;g=ENSBTAG00000027490) |
| 16 | 70,090,001 | 70,670,000 | 580,000 | Karvadi | [*KCNK2*](http://www.ensembl.org/bos_taurus/Gene/Summary?db=core;g=ENSBTAG00000004407)*,* [*CENPF*](http://www.ensembl.org/bos_taurus/Gene/Summary?db=core;g=ENSBTAG00000024449)*,* [*PTPN14*](http://www.ensembl.org/bos_taurus/Gene/Summary?db=core;g=ENSBTAG00000021553) |
| 17 | 41,040,000 | 42,020,000 | 980,001 | Karvadi | [*C17H4orf45*](http://www.ensembl.org/bos_taurus/Gene/Summary?db=core;g=ENSBTAG00000033547)*,* [*FNIP2*](http://www.ensembl.org/bos_taurus/Gene/Summary?db=core;g=ENSBTAG00000011970)*,* [*PPID*](http://www.ensembl.org/bos_taurus/Gene/Summary?db=core;g=ENSBTAG00000016680)*,* [*ETFDH*](http://www.ensembl.org/bos_taurus/Gene/Summary?db=core;g=ENSBTAG00000016679)*,* [*C17H4orf46*](http://www.ensembl.org/bos_taurus/Gene/Summary?db=core;g=ENSBTAG00000033486)*,* [*RXFP1*](http://www.ensembl.org/bos_taurus/Gene/Summary?db=core;g=ENSBTAG00000010306)*,* [*TMEM144*](http://www.ensembl.org/bos_taurus/Gene/Summary?db=core;g=ENSBTAG00000019229)*,* [*FAM198B*](http://www.ensembl.org/bos_taurus/Gene/Summary?db=core;g=ENSBTAG00000017069) |
| 19 | 27,190,000 | 27,930,000 | 740,001 | Karvadi | [*PSMB6*](http://www.ensembl.org/bos_taurus/Gene/Summary?db=core;g=ENSBTAG00000013390)*,* [*GLTPD2*](http://www.ensembl.org/bos_taurus/Gene/Summary?db=core;g=ENSBTAG00000048034)*,* [*VMO1*](http://www.ensembl.org/bos_taurus/Gene/Summary?db=core;g=ENSBTAG00000045946)*,* [*TM4SF5*](http://www.ensembl.org/bos_taurus/Gene/Summary?db=core;g=ENSBTAG00000003733)*,* [*ZMYND15*](http://www.ensembl.org/bos_taurus/Gene/Summary?db=core;g=ENSBTAG00000018316)*,* [*CXCL16*](http://www.ensembl.org/bos_taurus/Gene/Summary?db=core;g=ENSBTAG00000031998)*,* [*MED11*](http://www.ensembl.org/bos_taurus/Gene/Summary?db=core;g=ENSBTAG00000018314)*,* [*ARRB2*](http://www.ensembl.org/bos_taurus/Gene/Summary?db=core;g=ENSBTAG00000035587)*,* [*PELP1*](http://www.ensembl.org/bos_taurus/Gene/Summary?db=core;g=ENSBTAG00000018315)*,* [*ALOX15*](http://www.ensembl.org/bos_taurus/Gene/Summary?db=core;g=ENSBTAG00000011990)*,* [*ALOX12E*](http://www.ensembl.org/bos_taurus/Gene/Summary?db=core;g=ENSBTAG00000031933)*,* [*ALOX12*](http://www.ensembl.org/bos_taurus/Gene/Summary?db=core;g=ENSBTAG00000021933)*,* [*RNASEK*](http://www.ensembl.org/bos_taurus/Gene/Summary?db=core;g=ENSBTAG00000021932)*,* [*C19H17orf49*](http://www.ensembl.org/bos_taurus/Gene/Summary?db=core;g=ENSBTAG00000021931)*,*  [*BCL6B*](http://www.ensembl.org/bos_taurus/Gene/Summary?db=core;g=ENSBTAG00000020854)*,* [*SLC16A13*](http://www.ensembl.org/bos_taurus/Gene/Summary?db=core;g=ENSBTAG00000020853) |
| 19 | 46,630,000 | 47,700,000 | 1,070,001 | Golias | [*MAPT*](http://www.ensembl.org/bos_taurus/Gene/Summary?db=core;g=ENSBTAG00000017512)*,* [*KANSL1*](http://www.ensembl.org/bos_taurus/Gene/Summary?db=core;g=ENSBTAG00000012564)*,* [*CDC27*](http://www.ensembl.org/bos_taurus/Gene/Summary?db=core;g=ENSBTAG00000002726)*,* [*MYL4*](http://www.ensembl.org/bos_taurus/Gene/Summary?db=core;g=ENSBTAG00000021916)*,* [*ITGB3*](http://www.ensembl.org/bos_taurus/Gene/Summary?db=core;g=ENSBTAG00000009987)*,* [*EFCAB3*](http://www.ensembl.org/bos_taurus/Gene/Summary?db=core;g=ENSBTAG00000006943)*,* [*METTL2A*](http://www.ensembl.org/bos_taurus/Gene/Summary?db=core;g=ENSBTAG00000009458)*,* [*TLK2*](http://www.ensembl.org/bos_taurus/Gene/Summary?db=core;g=ENSBTAG00000015713) |
| 20 | 56,950,000 | 57,560,000 | 610,001 | Karvadi | [*MARCH11*](http://www.ensembl.org/bos_taurus/Gene/Summary?db=core;g=ENSBTAG00000037576)*,* [*FBXL7*](http://www.ensembl.org/bos_taurus/Gene/Summary?db=core;g=ENSBTAG00000003219) |
| 20 | 66,510,000 | 67,210,000 | 700,001 | Golias | [*PAPD7*](http://www.ensembl.org/bos_taurus/Gene/Summary?db=core;g=ENSBTAG00000003381)*,* [*SRD5A1*](http://www.ensembl.org/bos_taurus/Gene/Summary?db=core;g=ENSBTAG00000015478)*,* [*NSUN2*](http://www.ensembl.org/bos_taurus/Gene/Summary?db=core;g=ENSBTAG00000015466)*,* [*MED10*](http://www.ensembl.org/bos_taurus/Gene/Summary?db=core;g=ENSBTAG00000008130) |
| 20 | 67,330,000 | 67,880,000 | 550,001 | Golias | [*ICE1*](http://www.ensembl.org/bos_taurus/Gene/Summary?db=core;g=ENSBTAG00000018501) |
| 21 | 65,280,000 | 65,890,000 | 610,001 | Karvadi | [*BCL11B*](http://www.ensembl.org/bos_taurus/Gene/Summary?db=core;g=ENSBTAG00000018019) |
| 22 | 34,360,000 | 34,840,000 | 480,001 | Karvadi | [*KBTBD8*](http://www.ensembl.org/bos_taurus/Gene/Summary?db=core;g=ENSBTAG00000011081) |
| 23 | 43,440 | 1,253,000 | 1,209,561 | Karvadi | [*KHDRBS2*](http://www.ensembl.org/bos_taurus/Gene/Summary?db=core;g=ENSBTAG00000043990) |
| 23 | 65,280,000 | 65,890,000 | 610,001 | Karvadi | *-* |
| 24 | 42,400,000 | 42,760,000 | 360,001 | Akasamu | *APCDD1, NAPG, PIEZO2* |
| 25 | 30,270,000 | 31,560,000 | 1,290,001 | Nagpur | *-* |
| 26 | 1,961,000 | 3,250,000 | 1,289,001 | Karvadi | [*ZWINT*](http://www.ensembl.org/bos_taurus/Gene/Summary?db=core;g=ENSBTAG00000002655) |
| 26 | 15,670,000 | 16,650,000 | 980,001 | Karvadi | [*PLCE1*](http://www.ensembl.org/bos_taurus/Gene/Summary?db=core;g=ENSBTAG00000018966)*,* [*NOC3L*](http://www.ensembl.org/bos_taurus/Gene/Summary?db=core;g=ENSBTAG00000011368)*,* [*TBC1D12*](http://www.ensembl.org/bos_taurus/Gene/Summary?db=core;g=ENSBTAG00000009693)*,* [*HELLS*](http://www.ensembl.org/bos_taurus/Gene/Summary?db=core;g=ENSBTAG00000005979)*,* [*CYP2C18*](http://www.ensembl.org/bos_taurus/Gene/Summary?db=core;g=ENSBTAG00000037559)*,* [*CYP2C87*](http://www.ensembl.org/bos_taurus/Gene/Summary?db=core;g=ENSBTAG00000037795)*,* [*CYP2C19*](http://www.ensembl.org/bos_taurus/Gene/Summary?db=core;g=ENSBTAG00000023549)*,* [*PDLIM1*](http://www.ensembl.org/bos_taurus/Gene/Summary?db=core;g=ENSBTAG00000011182) |
| 26 | 41,730,000 | 42,340,000 | 610,001 | Karvadi | [*FGFR2*](http://www.ensembl.org/bos_taurus/Gene/Summary?db=core;g=ENSBTAG00000014064)*,* [*ATE1*](http://www.ensembl.org/bos_taurus/Gene/Summary?db=core;g=ENSBTAG00000003178)*,* [*NSMCE4A*](http://www.ensembl.org/bos_taurus/Gene/Summary?db=core;g=ENSBTAG00000019166)*,* [*TACC2*](http://www.ensembl.org/bos_taurus/Gene/Summary?db=core;g=ENSBTAG00000010786) |
| 27 | 18,860,000 | 19,960,000 | 1,100,001 | Godhavari | [*MTMR7*](http://www.ensembl.org/bos_taurus/Gene/Summary?db=core;g=ENSBTAG00000010372)*,* [*VPS37A*](http://www.ensembl.org/bos_taurus/Gene/Summary?db=core;g=ENSBTAG00000010355)*,* [*CNOT7*](http://www.ensembl.org/bos_taurus/Gene/Summary?db=core;g=ENSBTAG00000018036)*,* [*ZDHHC2*](http://www.ensembl.org/bos_taurus/Gene/Summary?db=core;g=ENSBTAG00000044194)*,* [*MICU3*](http://www.ensembl.org/bos_taurus/Gene/Summary?db=core;g=ENSBTAG00000020468)*,* [*FGF20*](http://www.ensembl.org/bos_taurus/Gene/Summary?db=core;g=ENSBTAG00000044043) |
| 28 | 18,760,000 | 19,880,000 | 1,120,001 | Godhavari | [*ADO*](http://www.ensembl.org/bos_taurus/Gene/Summary?db=core;g=ENSBTAG00000045717)*,* [*EGR2*](http://www.ensembl.org/bos_taurus/Gene/Summary?db=core;g=ENSBTAG00000046409)*,* [*NRBF2*](http://www.ensembl.org/bos_taurus/Gene/Summary?db=core;g=ENSBTAG00000012476)*,* [*JMJD1C*](http://www.ensembl.org/bos_taurus/Gene/Summary?db=core;g=ENSBTAG00000001573)*,* [*REEP3*](http://www.ensembl.org/bos_taurus/Gene/Summary?db=core;g=ENSBTAG00000019755) |
| ^1^ BTA: *Bos taurus* autosome. | | | | |  |
