# Supplementary material for: Autozygosity islands and ROH patterns in Nellore lineages: evidence of selection for functionally important traits
Source: BMC Genomics. 2018 Sep 17;19:680. doi: 10.1186/s12864-018-5060-8 (PMC6142381; doi:10.1186/s12864-018-5060-8)
Supplement: Supplementary file 5 — Autozygosity islands within the genotyped animals (red) and those with lineages records (black). (PDF 29 kb) [file 12864_2018_5060_MOESM5_ESM.pdf]

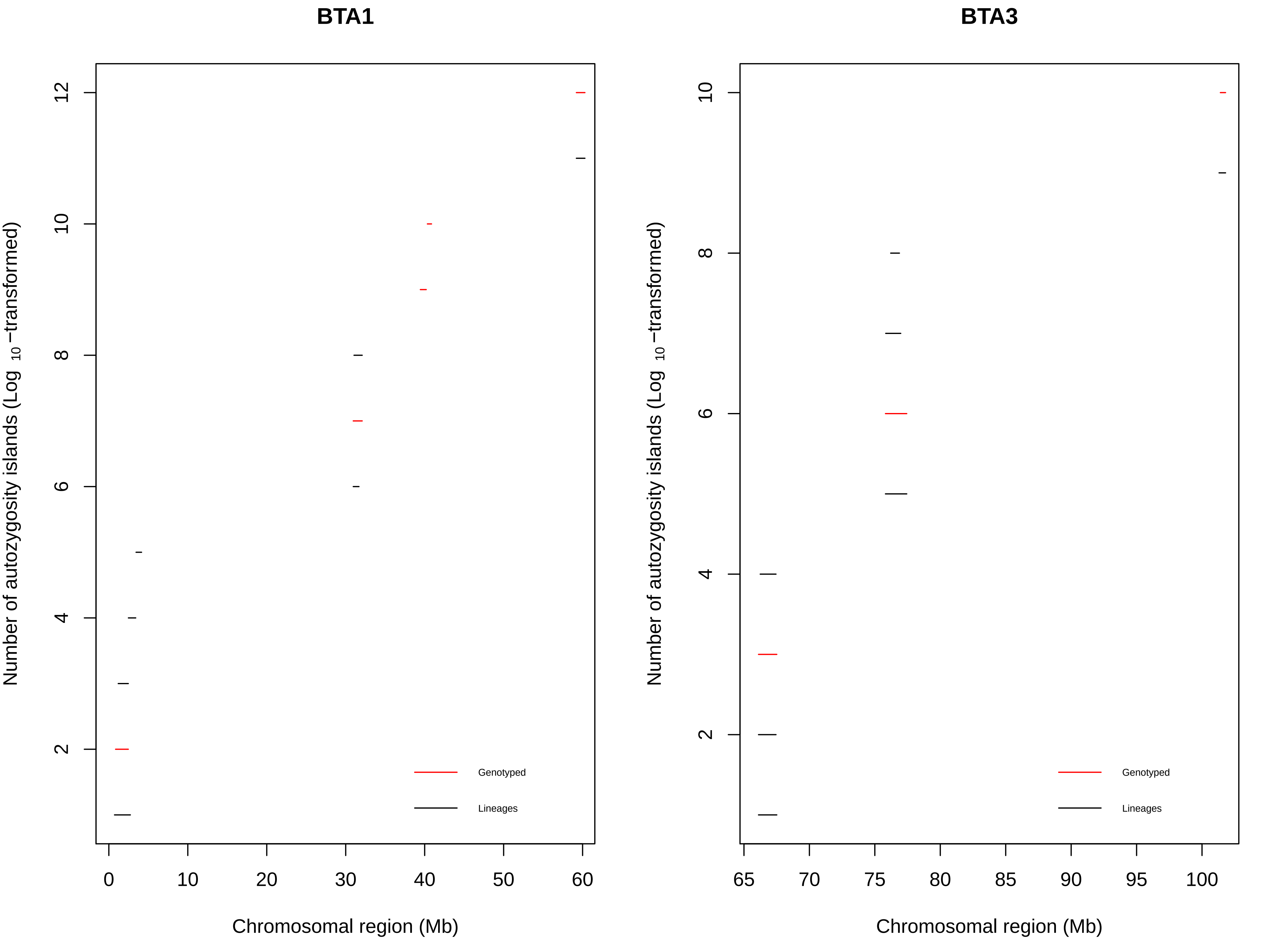
BTA1
BTA3
Number of autozygosity islands (Log 10 −transformed)
Chromosomal region (Mb)
Genotyped
Lineages
0
10
20
30
40
50
60
65
70
75
80
85
90
95
100
2
4
6
8
10
12

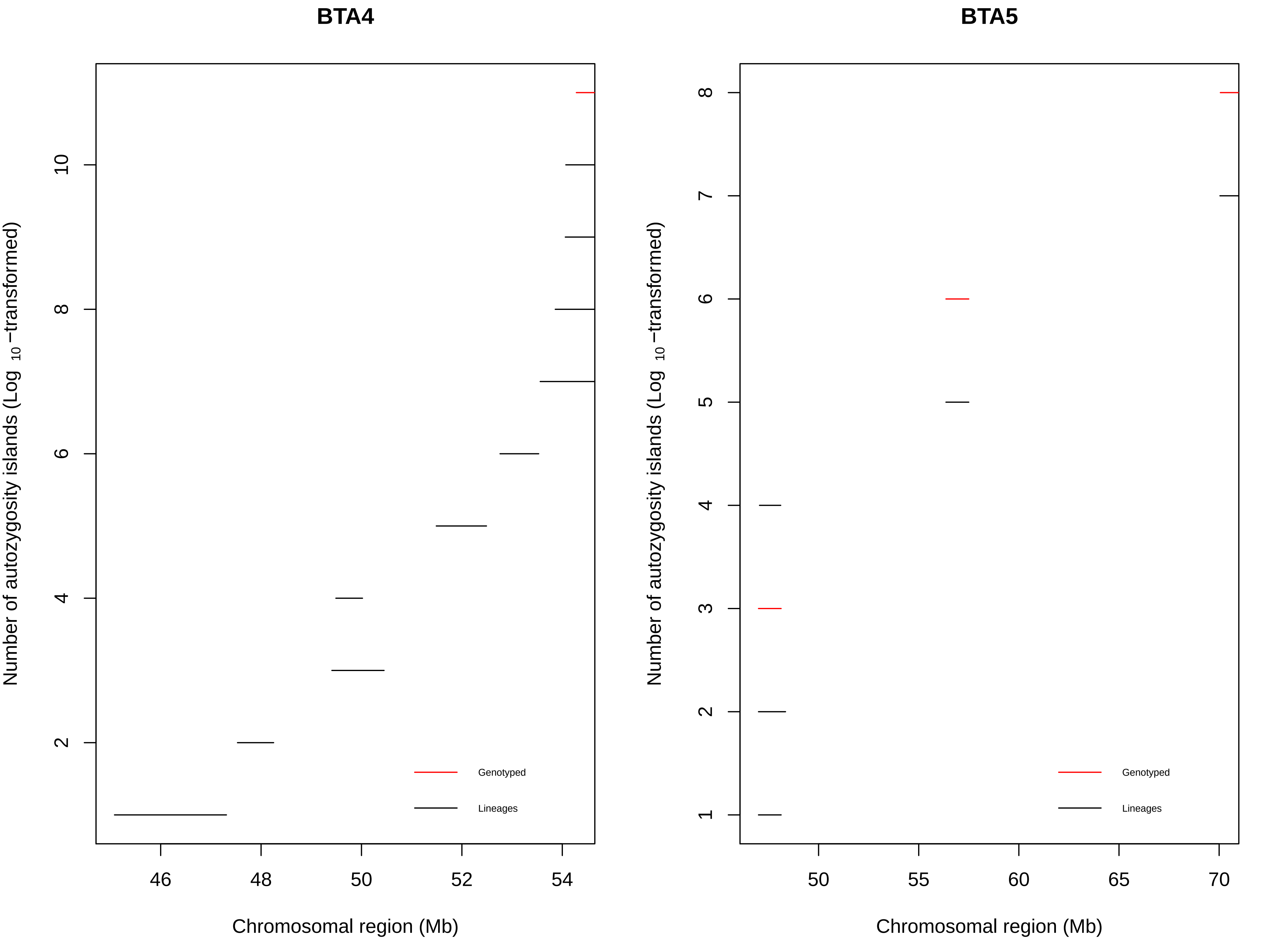

BTA4
BTA5
Number of autozygosity islands (Log 10 −transformed)
Chromosomal region (Mb)
Genotyped
Lineages
2
4
6
8
10
46
48
50
52
54
1
2
3
4
5
6
7
8
50
55
60
65
70

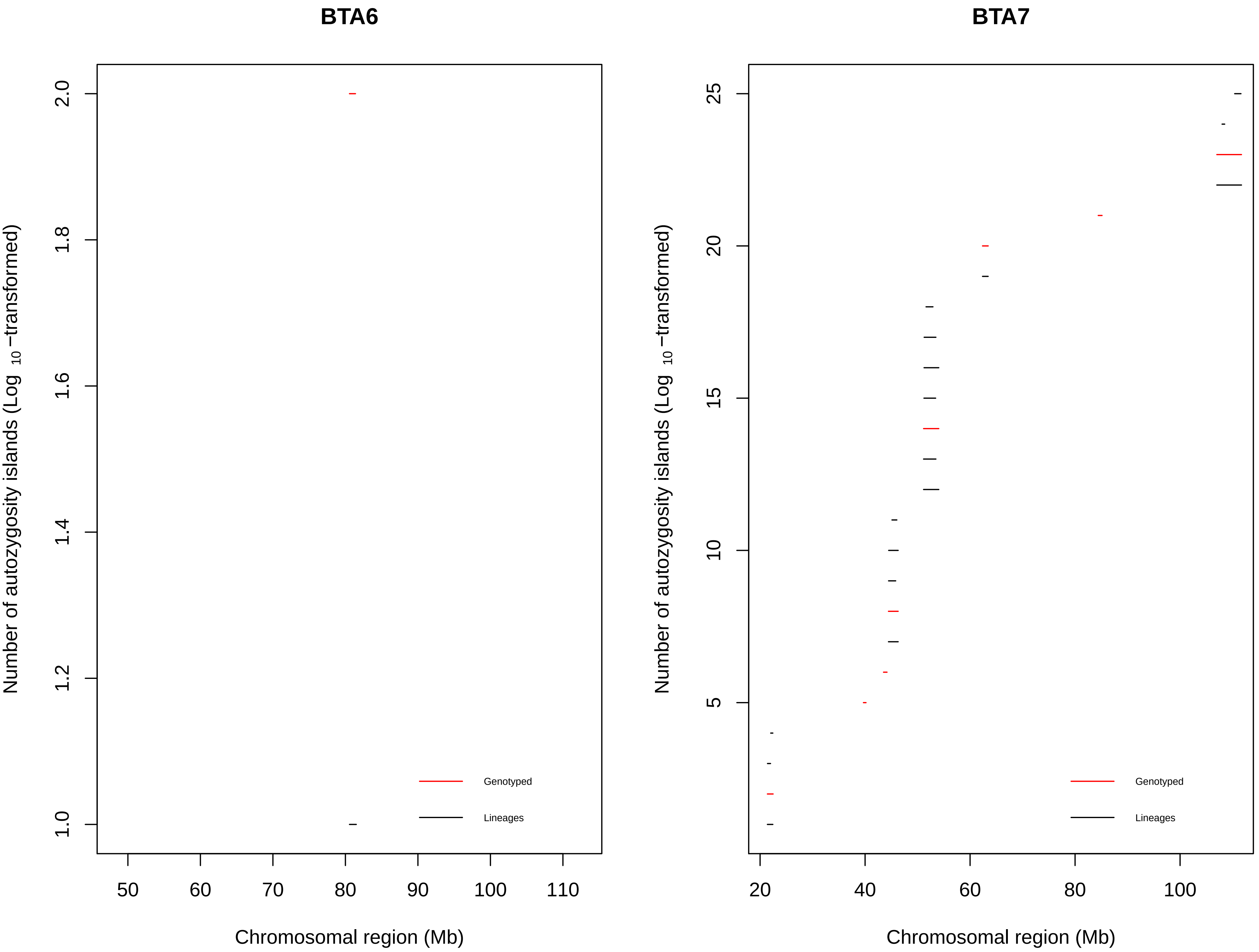

BTA6
Number of autozygosity islands (Log 10 −transformed)
2.0
1.8
1.6
1.4
1.2
1.0
50
60
70
80
90
100
110
Chromosomal region (Mb)
Genotyped
Lineages
BTA7
25
20
15
10
5
20
40
60
80
100

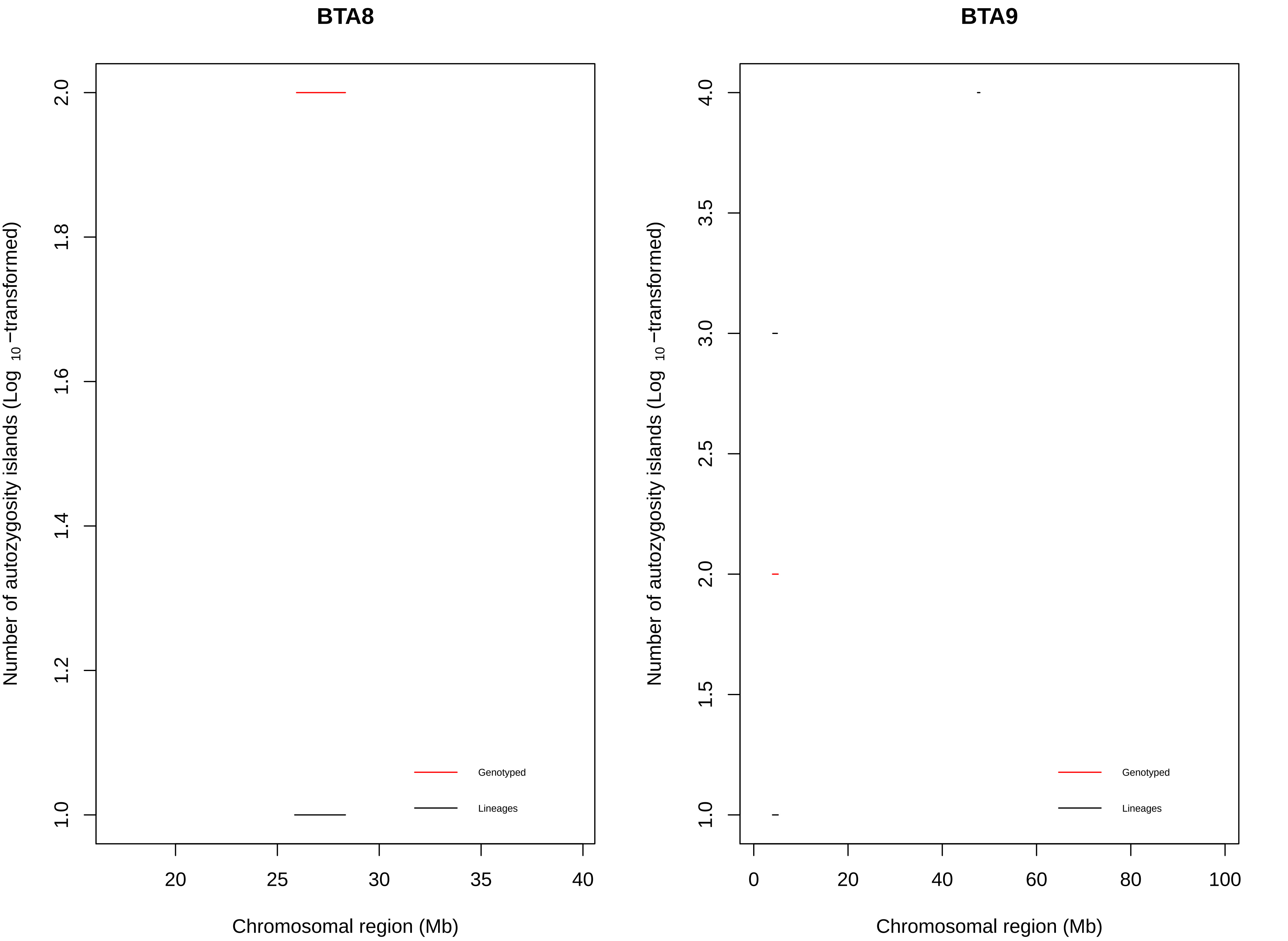
BTA8
BTA9
Number of autozygosity islands (Log 10 −transformed)
Chromosomal region (Mb)
Genotyped
Lineages
2.0
1.8
1.6
1.4
1.2
1.0
20
25
30
35
40
4.0
3.5
3.0
2.5
2.0
1.5
0
60
80
100

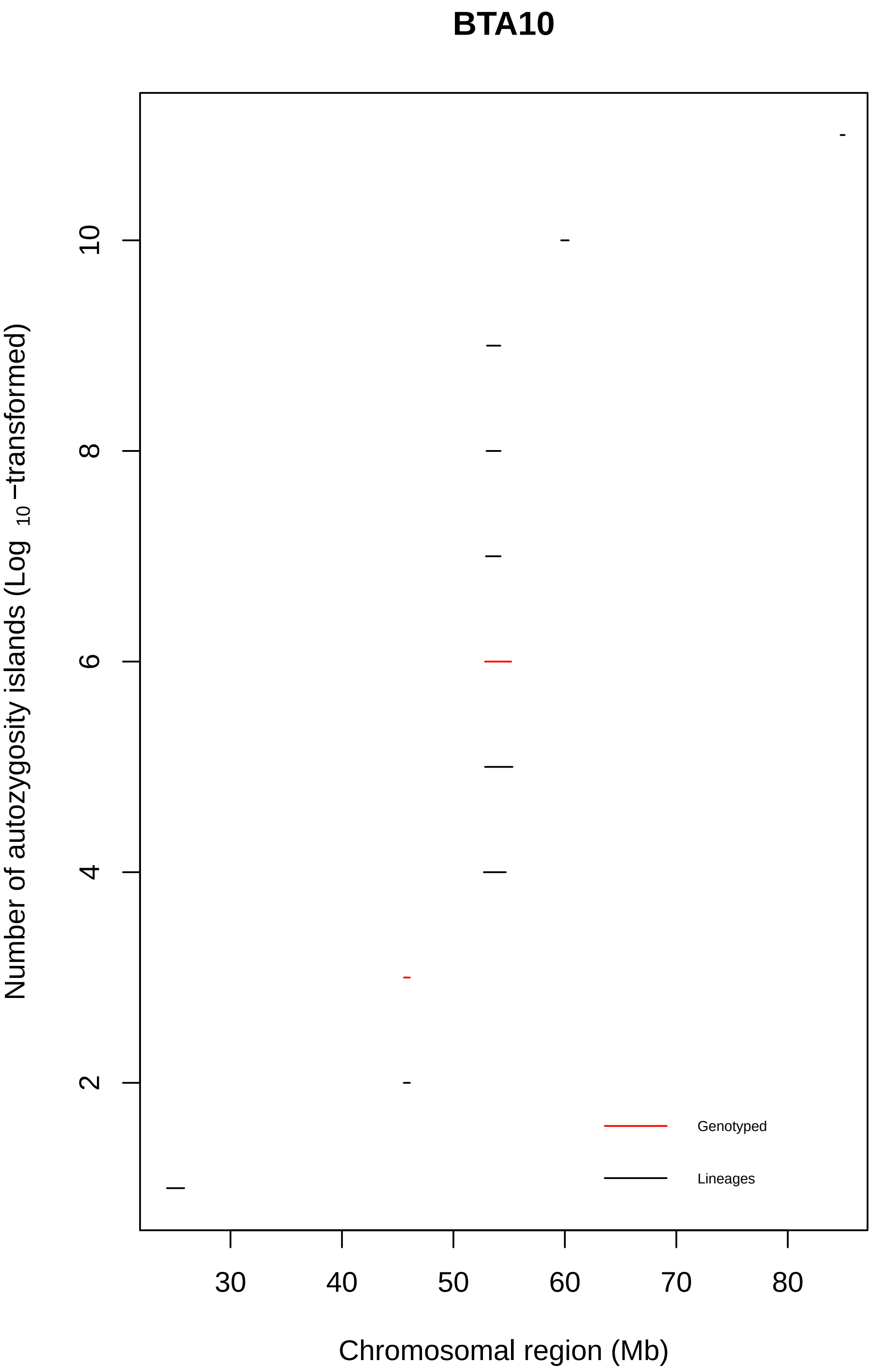
BTA10
Number of autozygosity islands (Log 10 −transformed)
2
4
6
8
10
30
40
50
60
70
80
Chromosomal region (Mb)
Genotyped
Lineages

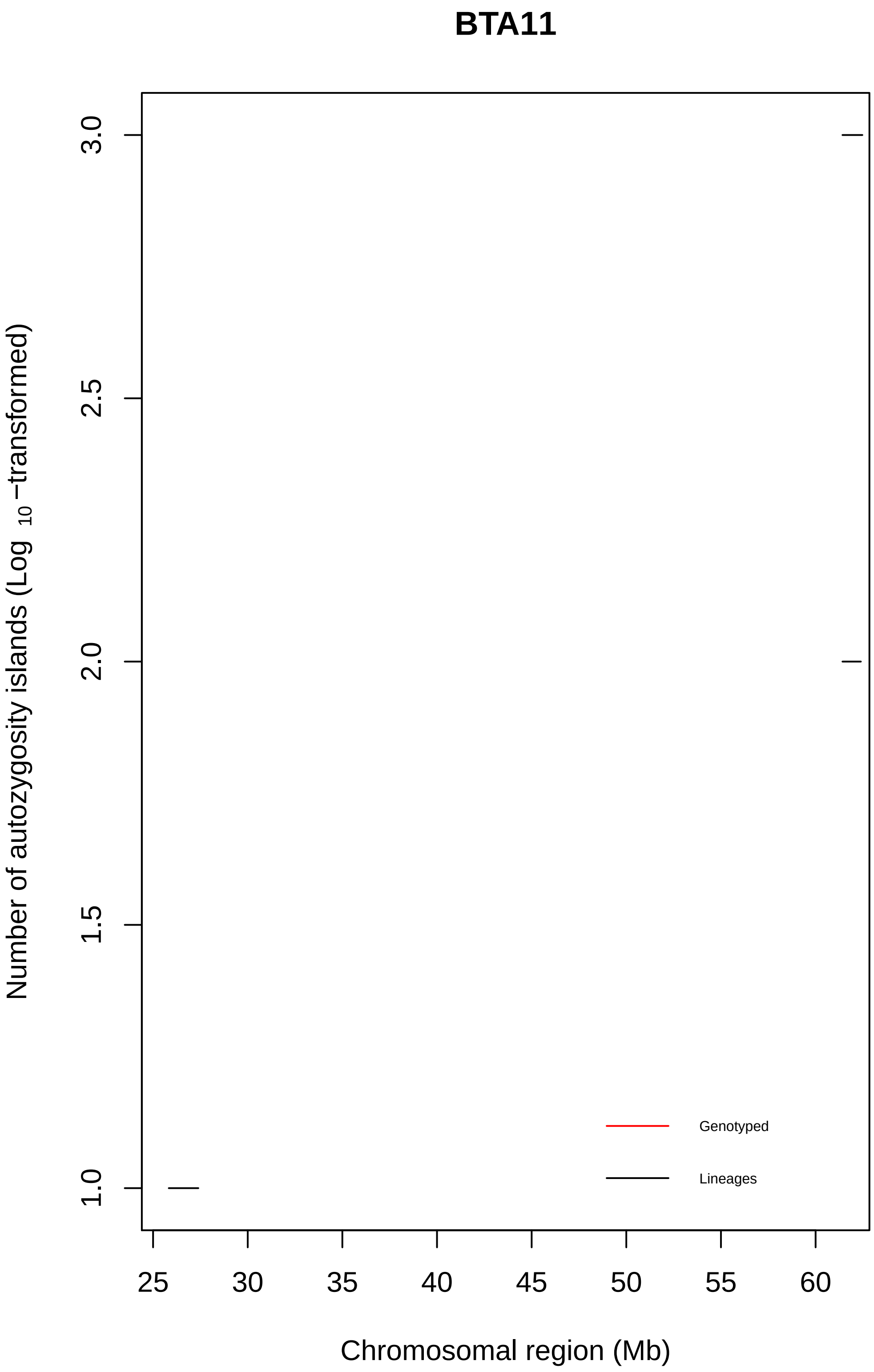
BTA11
Number of autozygosity islands (Log 10 −transformed)
1.0
1.5
2.0
2.5
3.0
25
30
35
40
45
50
55
60
Chromosomal region (Mb)
Genotyped
Lineages

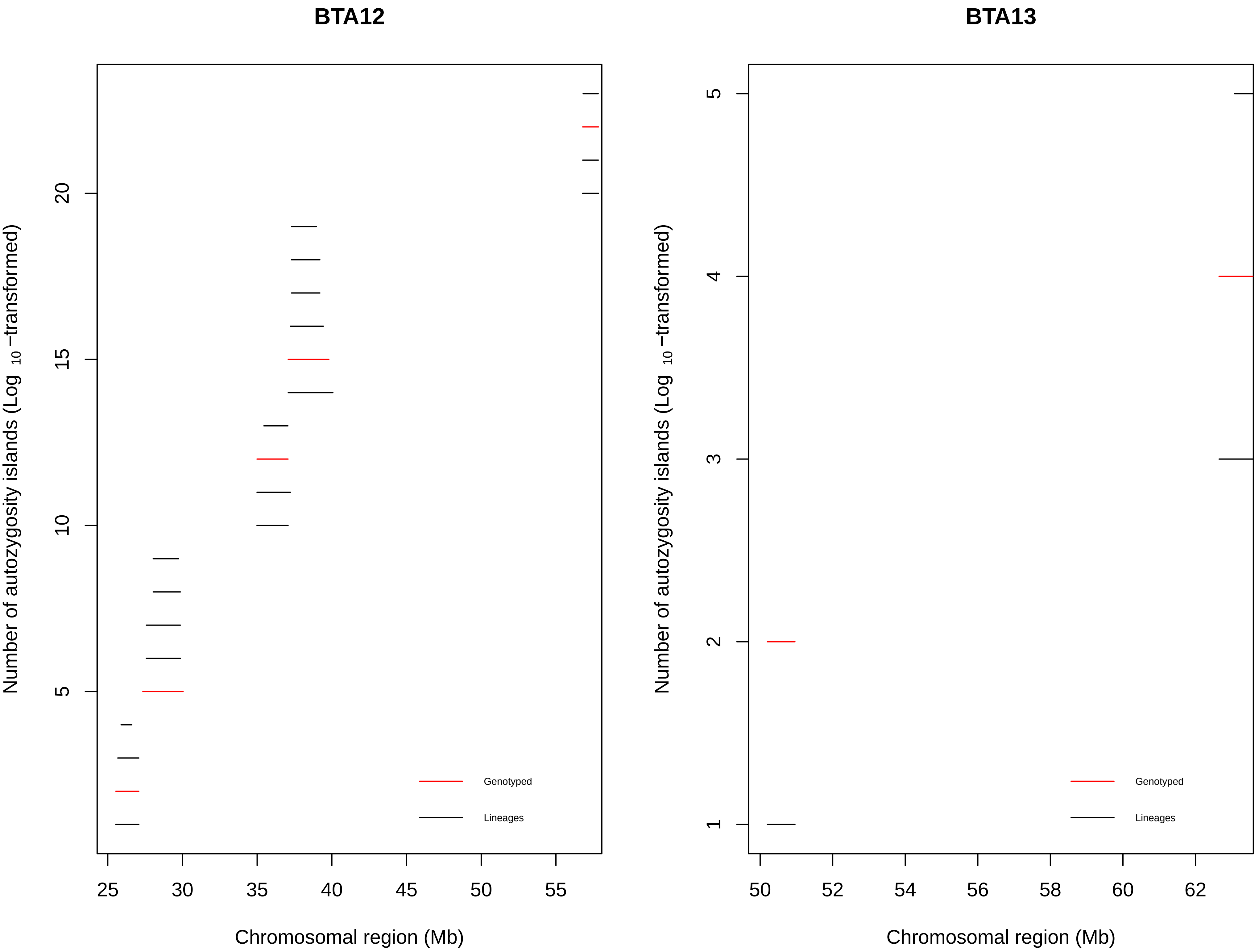
BTA12
BTA13
Number of autozygosity islands (Log 10 −transformed)
Chromosomal region (Mb)
Genotyped
Lineages
25
30
35
40
45
50
55
5
10
15
20
50
52
54
56
58
60
62
1
2
3
4
5

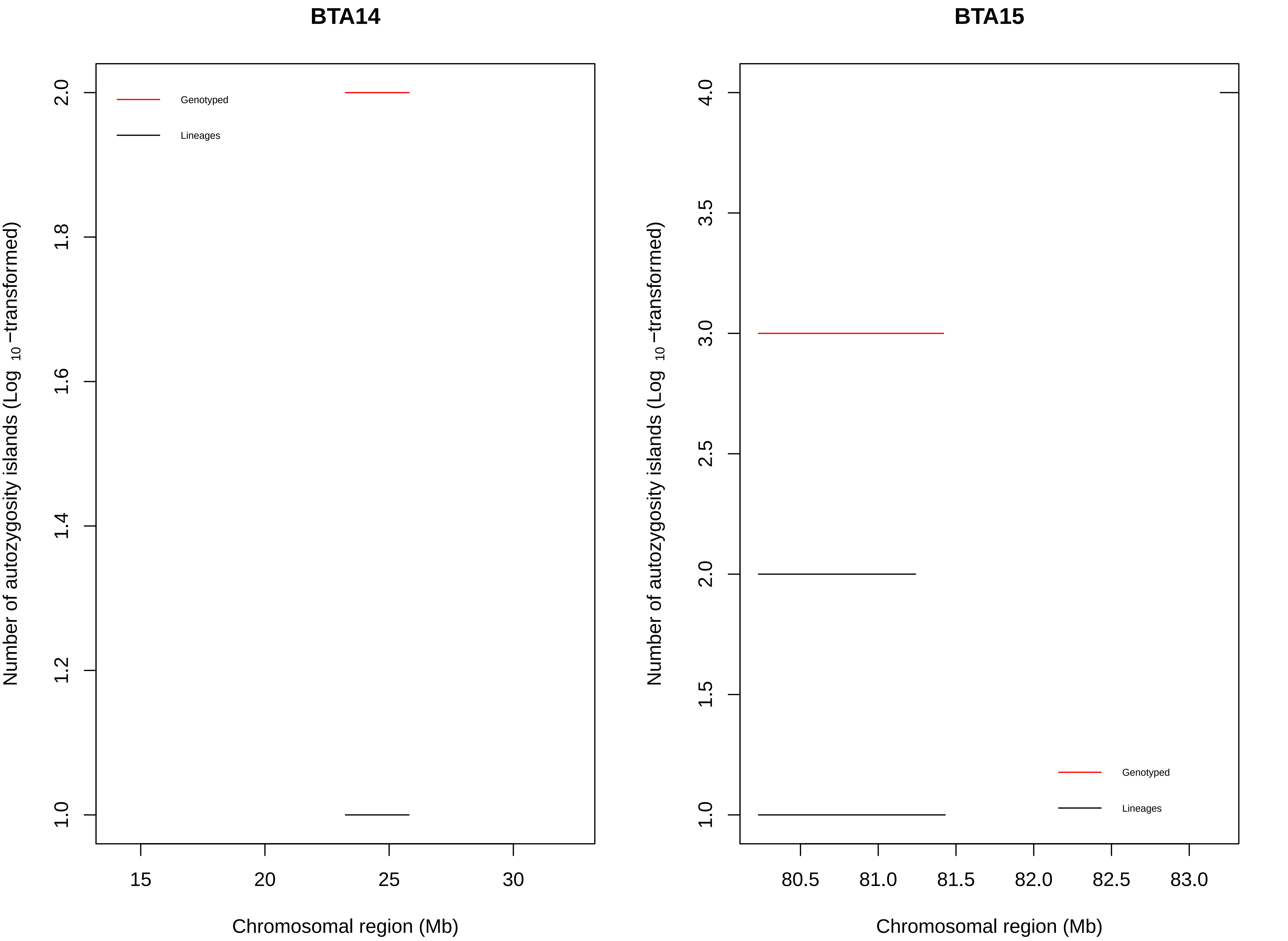

BTA14
Genotyped
Lineages
2.0
1.8
1.6
1.4
1.2
1.0
15
20
25
30
Number of autozygosity islands (Log 10 −transformed)
Chromosomal region (Mb)
BTA15
Genotyped
Lineages
4.0
3.5
3.0
2.5
2.0
1.5
1.0
80.5
81.0
81.5
82.0
82.5
83.0
Number of autozygosity islands (Log 10 −transformed)
Chromosomal region (Mb)

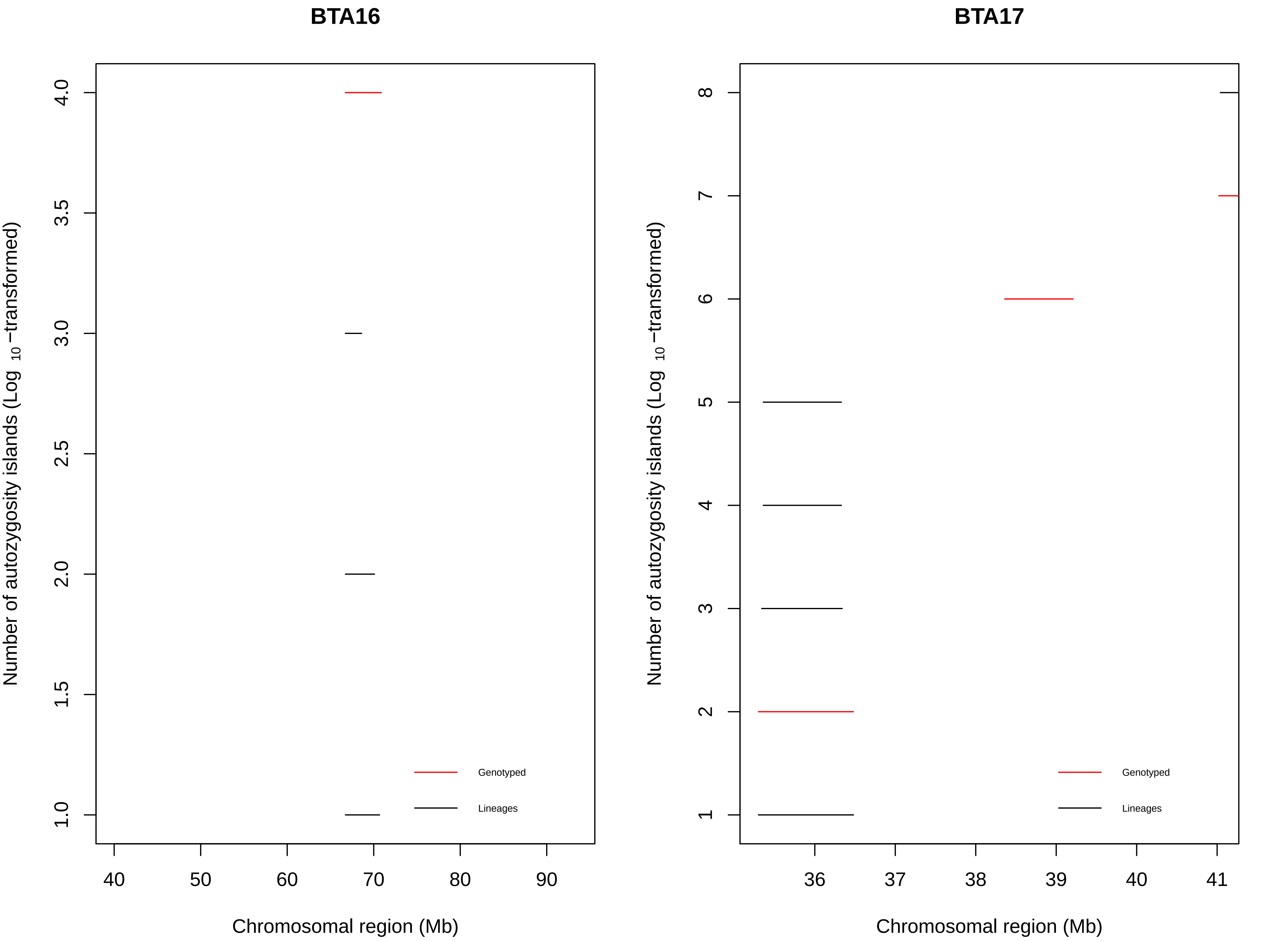
BTA16
Number of autozygosity islands (Log 10 −transformed)
4.0
3.5
3.0
2.5
2.0
1.5
1.0
40
50
60
70
80
90
Chromosomal region (Mb)
Genotyped
Lineages
BTA17
Number of autozygosity islands (Log 10 −transformed)
8
7
6
5
4
3
2
1
36
37
38
39
40
41
Chromosomal region (Mb)
Genotyped
Lineages

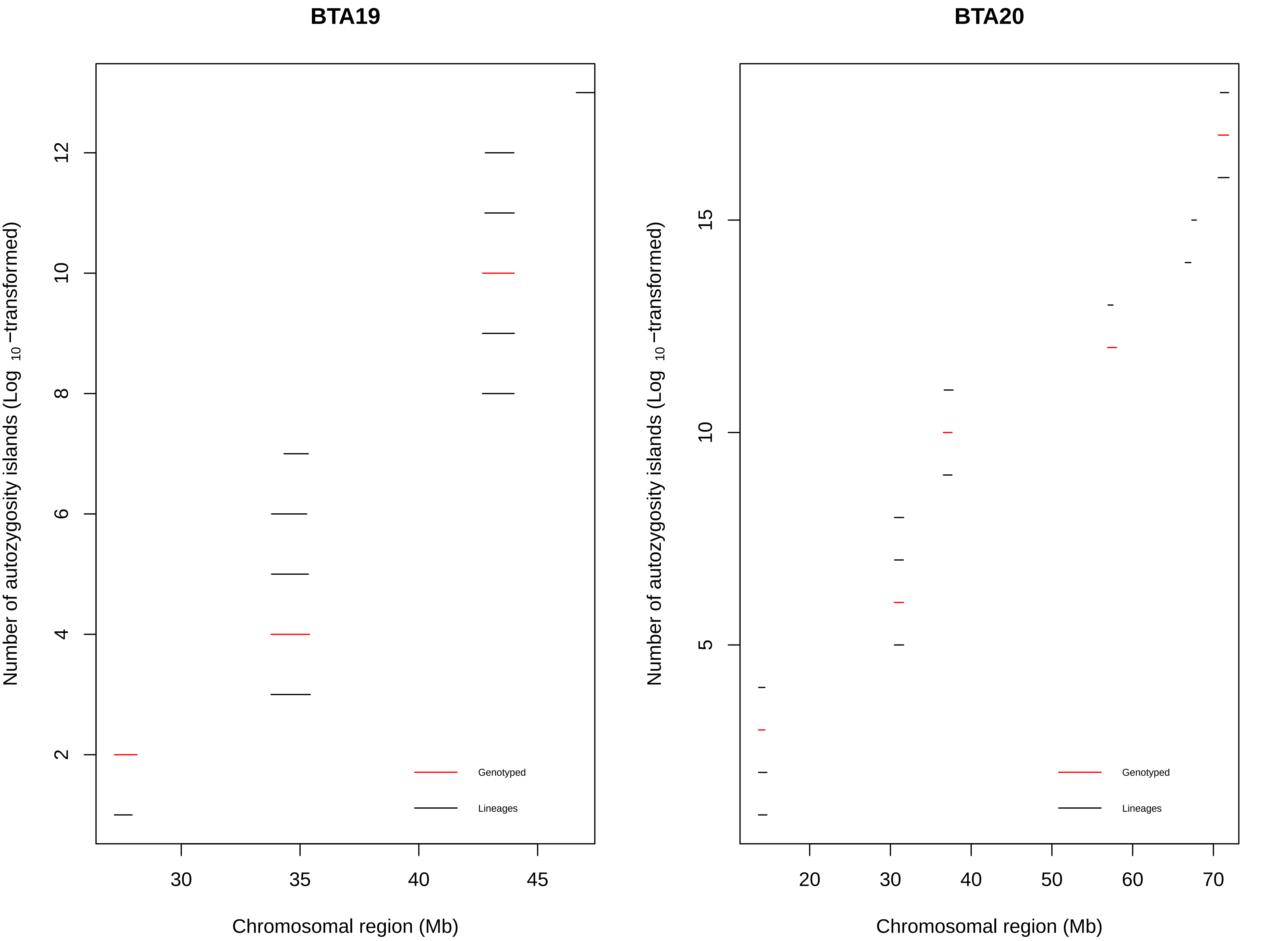

BTA19
BTA20
Number of autozygosity islands (Log 10 −transformed)
Chromosomal region (Mb)
Genotyped
Lineages
2
4
6
8
10
12
30
35
40
45
5
10
15
20
30
40
50
60
70

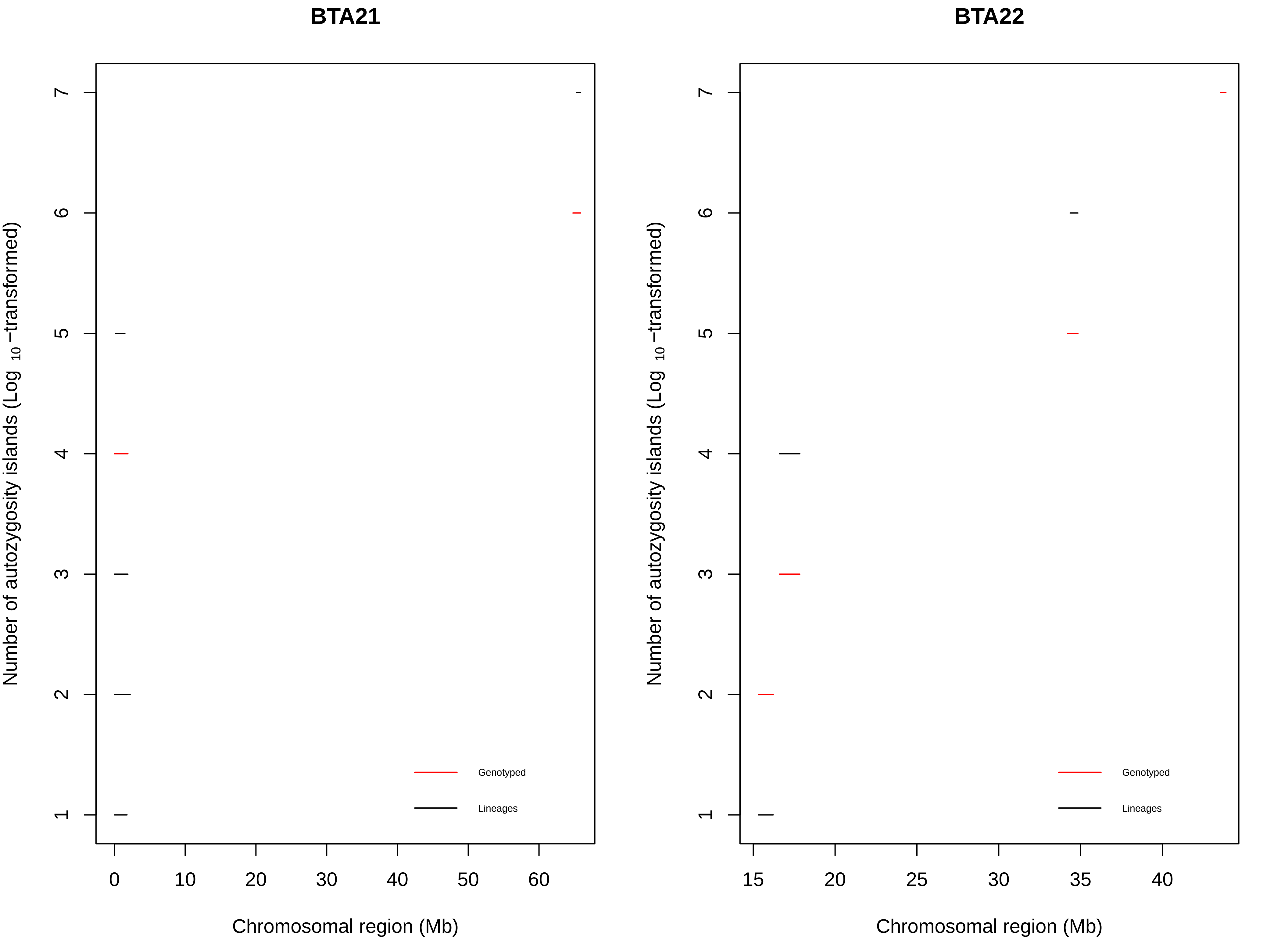

BTA21
BTA22
Number of autozygosity islands (Log 10 −transformed)
1
2
3
4
5
6
7
0
10
20
30
40
50
60
15
20
25
30
35
40
Chromosomal region (Mb)
Genotyped
Lineages

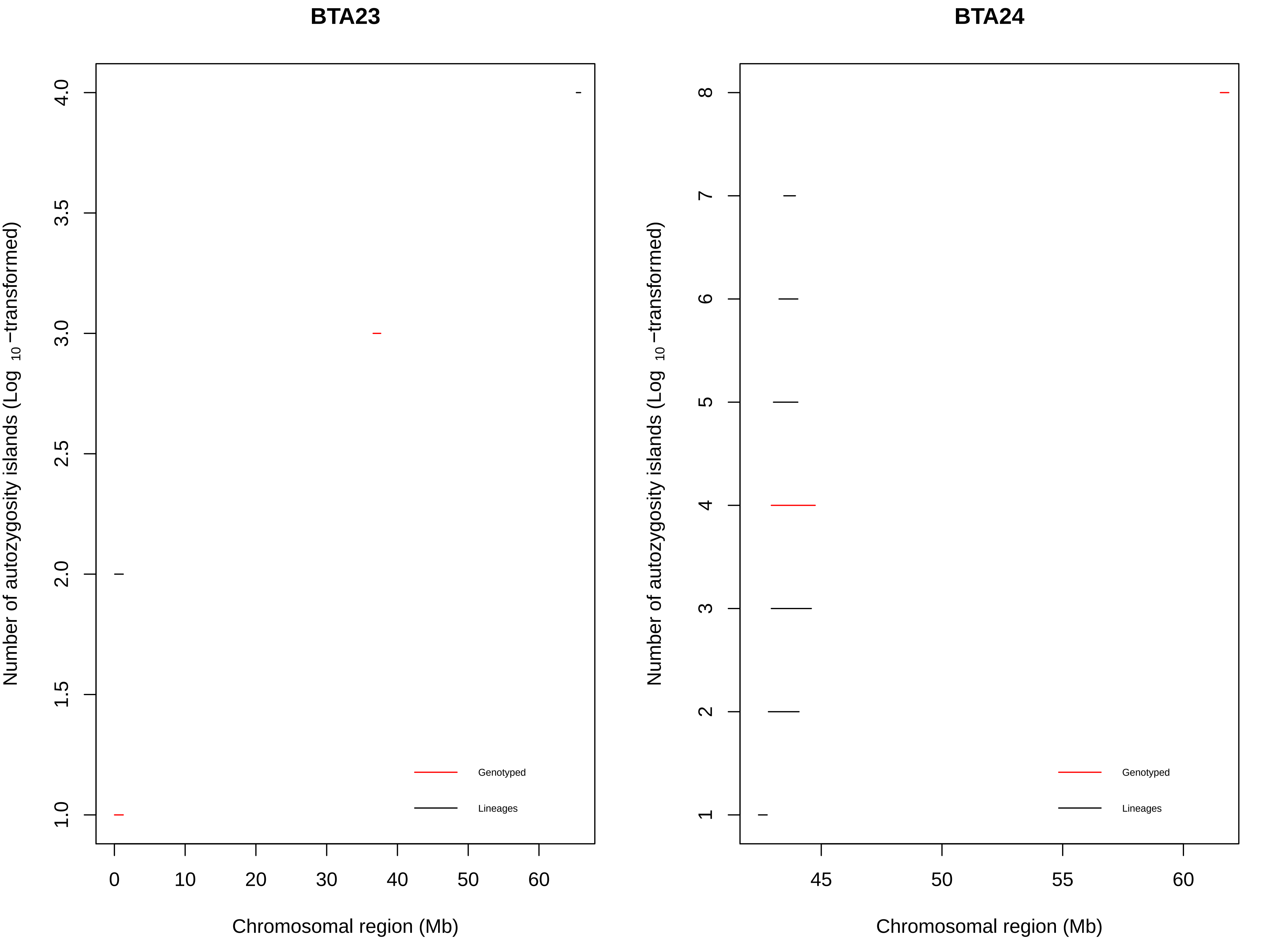

BTA23
BTA24
Number of autozygosity islands (Log 10 −transformed)
Chromosomal region (Mb)
Genotyped
Lineages
1.0
1.5
2.0
2.5
3.0
3.5
4.0
0
10
20
30
40
50
60
1
2
3
4
5
6
7
8
45
50
55
60

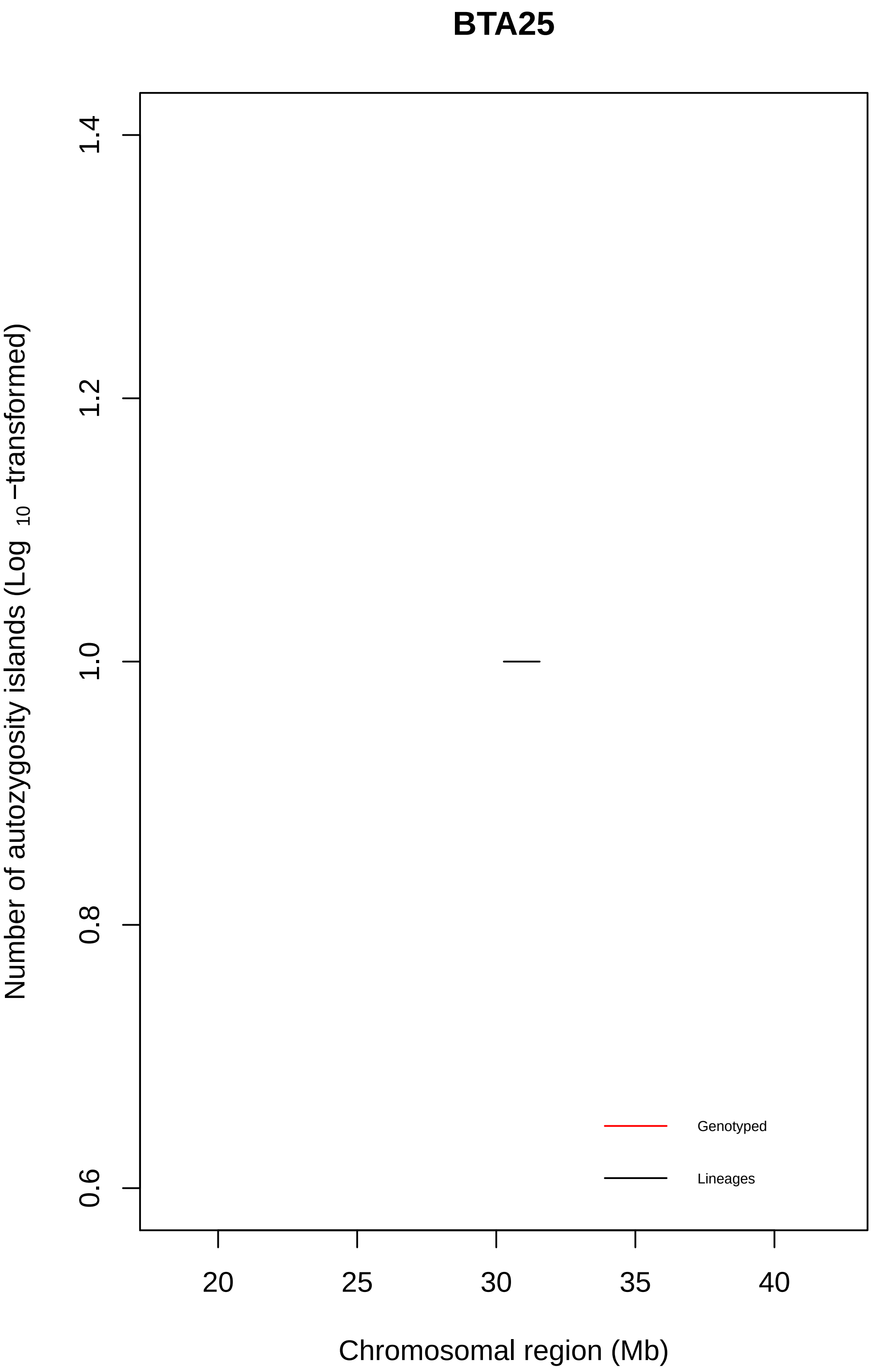

BTA25
Number of autozygosity islands (Log 10 −transformed)
1.4
1.2
1.0
0.8
0.6
20
25
30
35
40
Chromosomal region (Mb)
Genotyped
Lineages

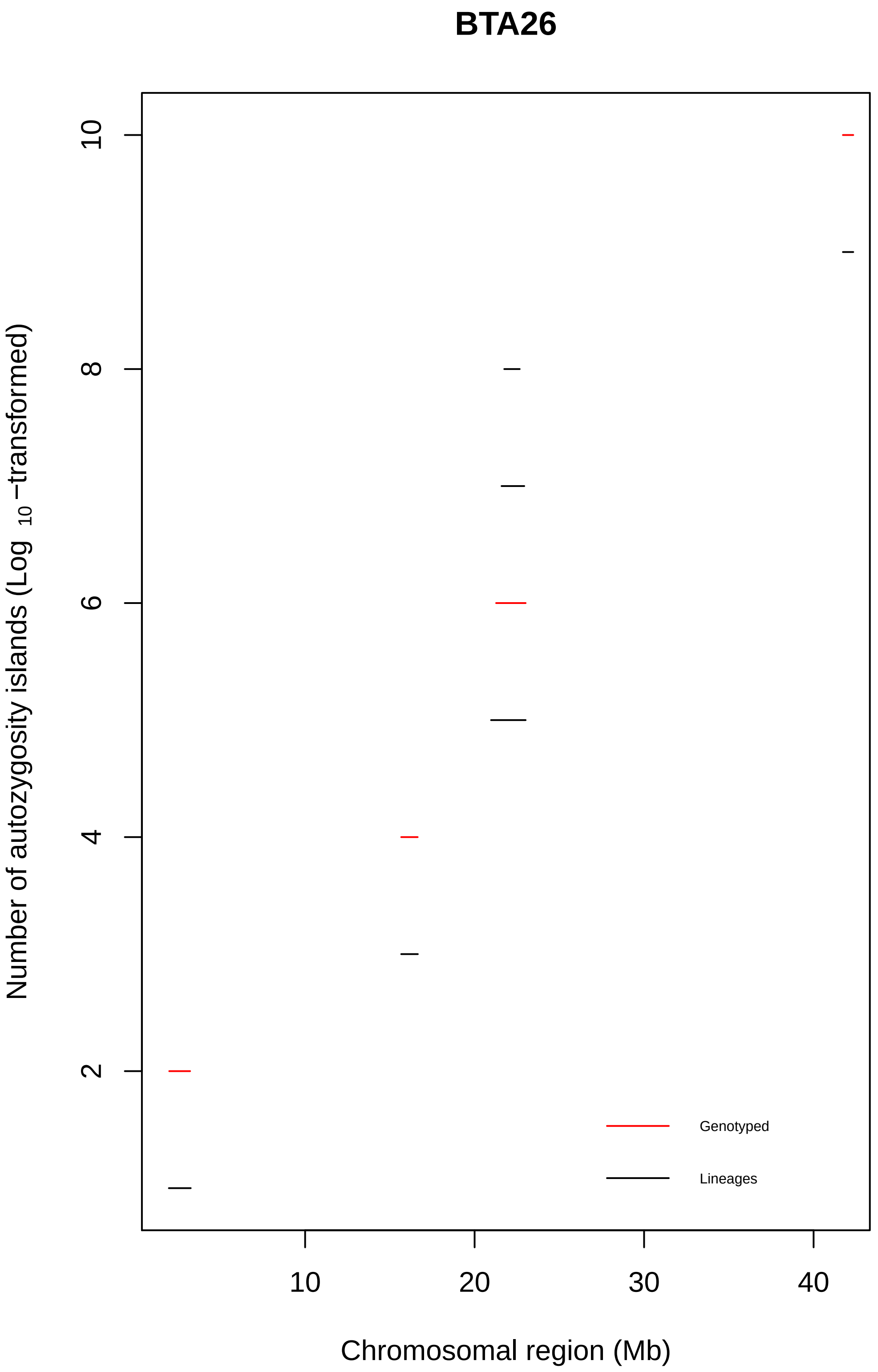

BTA26
Number of autozygosity islands (Log 10 −transformed)
10
8
6
4
2
10
20
30
40
Chromosomal region (Mb)
Genotyped
Lineages

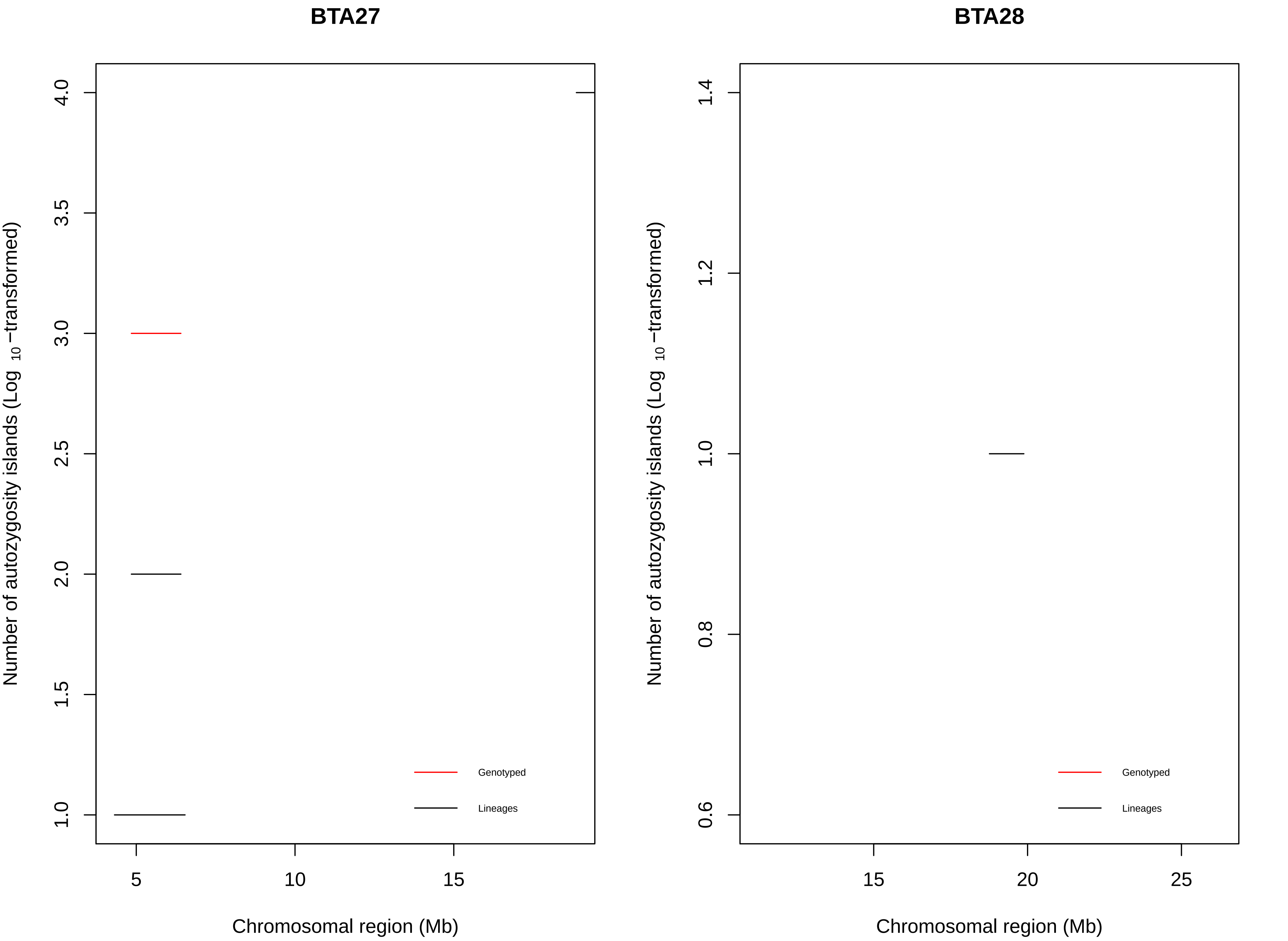
BTA27
BTA28
Number of autozygosity islands (Log 10 −transformed)
Chromosomal region (Mb)
Genotyped
Lineages
4.0
3.5
3.0
2.5
2.0
1.5
1.0
5
10
15
1.4
1.2
1.0
0.8
0.6
20
25

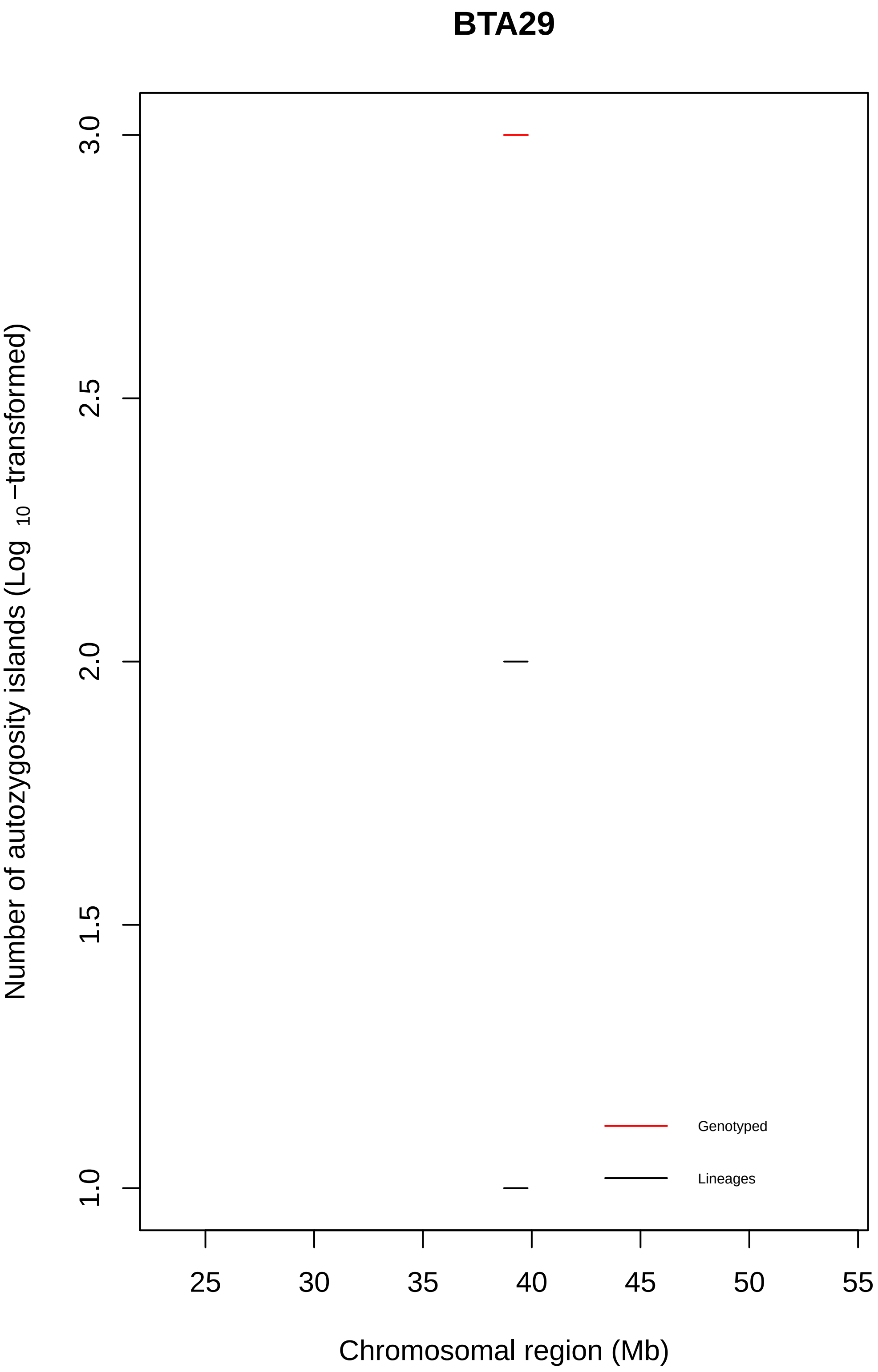

BTA29
Number of autozygosity islands (Log 10 −transformed)
1.0
1.5
2.0
2.5
3.0
25
30
35
40
45
50
55
Chromosomal region (Mb)
Genotyped
Lineages
