# Supplementary material for: Autozygosity islands and ROH patterns in Nellore lineages: evidence of selection for functionally important traits
Source: BMC Genomics. 2018 Sep 17;19:680. doi: 10.1186/s12864-018-5060-8 (PMC6142381; doi:10.1186/s12864-018-5060-8)
Supplement: Supplementary file 6 — Gene Ontology terms and KEGG pathways annotation analysis enriched (P < 0.01) based on autozygosity islands set of genes identified for the genotyped animals (n = 9386). (DOCX 17 kb) [file 12864_2018_5060_MOESM6_ESM.docx]

| Additional file 6. Genes from GO terms and KEGG pathways annotation analysis enriched (P<0.01) based on autozygosity islands set of genes identified for the genotyped animals (n=9,386) | |
| --- | --- |
| **Terms** | **Genes** |
| **GO Biological Process** |  |
| (GO:0042742) defense response to bacteria | *ELANE, ROMO1, TAP, DEFB4A, LEAP2, DEFB6, DEFB5, DEFB7, EBD, PENK, LAP, DEFB13, DEFB10, DEFB1* |
| (GO:0030163) protein catabolic process | *PAG17, PAG19, MGC157405, PAG16, MGC157408, PAG21, PAG20, PAG4, PAG1* |
| (GO:0070200) establishment of protein localization to telomere | *NABP2, WRAP53, BRCA2, TERT* |
| (GO:0040014) regulation of multicellular organism growth | *FGFR2, DRD3, GDF5, GAMT, AFG3L2, STAT3* |
| (GO:0045647) negative regulation of erythrocyte differentiation | *STAT5A, LDB1, STAT5B, HSPA9* |
| (GO:0030901) midbrain development | *FGFR2, KAT2A, RFX4, WLS, PITX3, UQCRQ* |
| **GO Molecular Function** |  |
| (GO:0008289) lipid binding | *BPIFB1, BPIFB2, BPIFA3, BPIFB3, BPIFB4, BPIFA1, BPIFB5, BPIFB6, BPIFA2A, BPIFA2B, FER, BPIFA2C, STARD13* |
| (GO:0004190) aspartic-type endopeptidase activity | *PAG17, PAG19, MGC157405, PAG16, MGC157408, PAG21, PAG20, PAG4, PAG1* |
| **GO Cellular Component** |  |
| (GO:0005776) autophagosome | *TBC1D12, MAP1LC3A, BECN1, NBR1, RAB24, USP33, TP53INP2, GABARAP* |
| (GO:0005634) nucleus | *RALY, RNMT, BTRC, STAT5A, STAT5B, DNASE1L3, TRMT1L, MIER2, RBMS2, ITCH, DND1, PITX3, CRY1, TGS1, SPATA24, PAN2, POLL, MAGEL2, LBX1, CSNK1G1, SUCLG2, CSNK1G2, PTBP1, PPARGC1B, DPCD, HSPB9, RFC3, NABP2, ARRB2, ZWINT, PYGO1, RAD18, MAPK7, FGFR2, STK11, SLF2, ZNF131, NOC3L, MUM1, AFAP1L1, IFI35, IRAK3, HECTD3, MNS1, TCTEX1D4, TCF3, HELLS, REEP6, SREBF1, PLAG1, DVL2, TRIP4, RFX4, PTPN2, BECN1, MICU2, SRA1, RFX7, ARID3A, BRCA2, DONSON, SMYD2, FXR2, SPRYD4, BRCA1, CIDEC, ATE1, R3HDM2, NSMCE4A, PPIB, R3HDM4, NEDD4, MLX, PPID, CPNE1, PPRC1, BCL6B, CIRBP, MAB21L1, NCOR1, TCF12, RAI1, FAM96A, ELF3, PHF23, NFKB2, CBFA2T2, LATS2, AES, TUBB6, FAM83G, IRAK2, EGR1, LYN, ELP5, TP53, TLE2, MBD3, HMGA2, SENP3, ZNF341, ZNF692, FANCD2, TPPP, NAB2, TOP3A, RBM39, UBB, GADD45B, CUEDC2, IRX4, NACA, POLR2E, NDN, POLR2K, ADAD1, PAXBP1, IVNS1ABP, STAT6, RNF126, RAX2, SNRK, BCL11B, TCEA1, KDM3B, NIM1K, TLX1, CSNK1A1, SHMT2, PDS5B, VHL, CS, CDC25C, TRIM23, CENPK, STAT3, STAT2, GPS2, RNF112, RGS20, PSMG2, BNC2, PSPC1, ZBTB4, PDC, GAMT, APBB3, NFIC, OGG1, TJP3, SCAND1, ALKBH5, MPHOSPH8, TP53INP2, ZMYND15* |
| (GO:0005815) microtubule organizing center | *CSNK1A1, SUCLG2, FLII, TCTEX1D4, AK5, RBM39, MAPRE1, PXK, LATS2, FNIP2* |
| (GO:0005730) nucleolus | *EIF6, ZNF554, MIDN, RNMT, NOC3L, NFS1, TIMM13, ZNF346, YBX2, CRYL1, WDR55, URB1, NPM3, TCEA1, RSL24D1, SDR9C7, TERT, RPS23, HSPA9, IK, VHL, TP53, ARID3A, THUMPD3, FGF22, TACC2, ACADVL, SENP3, PLK3, LRP1, NOLC1, TIMELESS, FANCD2, PPID, LLPH, PSPC1, ZZZ3, NFIC, ARL4D, MPHOSPH8, VPS25* |
| **KEGG pathway** |  |
| (bta01100) Metabolic pathways | *PTGES3, IMPAD1, IMPA2, PTGS2, ALOX12E, CYP2C18, SYNJ1, SAT2, PIP5K1C, ACSS2, UQCRQ, PRIM1, NDUFS7, GSS, NDUFS6, CRYL1, PIGL, UQCR11, PIGB, SUCLG2, PIGU, ATP6V1H, ACADVL, MAN2A1, NME5, PLCE1, G6PC, ALOX15, MTMR14, NNT, AOC2, UROD, AOC3, ALOX12, COASY, NAGLU, POLR2E, AHCY, HSD17B1, POLR2K, NDUFB8, NFS1, HMGCS1, CYP2C87, ALDH3A2, POLR2A, PLPP2, ALDH3A1, GLS2, SAO, LPCAT1, PEMT, HSD17B6, DNMT3B, SHMT1, SHMT2, NDUFA2, KL, NDUFA4L2, CS, AK5, ACLY, POLR3B, GART, PLA2G4A, GGT7, MBOAT1, ATP6V0A1, GAMT, CYP8B1, RDH16* |
