# Supplementary material for: Autozygosity islands and ROH patterns in Nellore lineages: evidence of selection for functionally important traits
Source: BMC Genomics. 2018 Sep 17;19:680. doi: 10.1186/s12864-018-5060-8 (PMC6142381; doi:10.1186/s12864-018-5060-8)
Supplement: Supplementary file 7 — Gene Ontology terms annotation analysis enriched (P < 0.01) based on copy number variation regions (CNVRs) and autozygosity islands overlapping regions set of genes identified for the genotyped animals (n = 9386). (DOCX 15 kb) [file 12864_2018_5060_MOESM7_ESM.docx]

| Additional file 7. Gene Ontology (GO) terms annotation analysis enriched (P<0.01) based on copy number variation regions (CNVRs) and autozygosity islands overlapping regions set of genes identified for the genotyped animals (n=9,386). | | | |
| --- | --- | --- | --- |
| **Gene Ontology Terms** | ***n* (Genes)** | **P-value** | **Genes** |
| **Biological Process** |  |  |  |
| GO:0040018 | 5 | 0.003 | *NIPBL, STAT5A, SLC6A3, STAT5B, HMGA2* |
| GO:0070200 | 3 | 0.006 | *NABP2, WRAP53, TERT* |
| GO:0042742 | 7 | 0.006 | *DEFB6, DEFB5, DEFB7, EBD, LAP, DEFB1, LEAP2* |
| GO:0007286 | 6 | 0.009 | *NME5, RNF17, STK11, ADAD1, AFF4, ZMYND15* |
| **Molecular Function** |  |  |  |
| GO:0004871 | 9 | 0.003 | *STAT6, GNAL, GNA11, STAT5A, ACAP1, STAT5B, CXXC5, STAT3, GNG7* |
| **KEGG** |  |  |  |
| bta05166 | 12 | 0.007 | *KAT2A, EGR1, DVL2, E2F3, APC2, STAT5A, STAT5B, TP53, NFKB2, TCF3, TERT, GPS2* |
