# Supplementary material for: Autozygosity islands and ROH patterns in Nellore lineages: evidence of selection for functionally important traits
Source: BMC Genomics. 2018 Sep 17;19:680. doi: 10.1186/s12864-018-5060-8 (PMC6142381; doi:10.1186/s12864-018-5060-8)
Supplement: Supplementary file 8 — Runs of homozygosity islands described in several cattle breeds located within those observed in the present study. (DOCX 22 kb) [file 12864_2018_5060_MOESM8_ESM.docx]

| Additional file 8. Runs of homozygosity islands described in several cattle breeds located within those observed in the present study. | | | |
| --- | --- | --- | --- |
| **Author** | **Cattle Breed** | **BTA** | **Physical Position (bp)** |
| (SÖLKNER et al., 2014) | Brahman, Gyr, and Nellore | 7 | 51,502,500:52,353,000^1^ |
|  |  | 12 | 28,434,000:29:628,100 |
|  |  | 21 | 1,360,390 :1,853,150^1^ |
| (GASPA et al., 2014) | Italian Holstein | 21 | 898,385:1,829,761^1^ |
|  |  | 26 | 211,146,794:23,000,155 |
| (SZMATOŁA et al., 2016) | Holstein | 7 | 42,440,064:43,592,173^1^ |
|  |  | 7 | 51,574,295:52,419,683^1^ |
|  |  | 14 | 24,220,070:25,351,733 |
|  |  | 20 | 28,329,720:32,293,167 |
|  |  | 22 | 22,004,775:23,984,012 |
|  |  | 29 | 37,782,301:39,905,644 |
|  | Red Polish | 1 | 31,206,393:31,659,179 |
|  |  | 7 | 51,574,295:54,081,460^1^ |
|  | Simmental | 7 | 42,645,056:45,383,502^1^ |
|  |  | 7 | 51,157,314:53,101,552^1^ |
|  |  | 14 | 23,853,811:24,326,513 |
|  | Limousin | 1 | 31,239,593:32,036,293 |
|  |  | 5 | 47,752,157:49,103,647 |
|  |  | 7 | 42,765,700:43,808,593^1^ |
|  |  | 7 | 53,101,552:53,859,609^1^ |
|  |  | 14 | 23,122,719:28,548,600 |
| (PERIPOLLI et al., 2018) | Gyr | 6 | 70,117,799:81,603,050 |
| ^1^ Autozygosity islands overlapping between these studies – current study. | | | |
