# Supplementary figures and images for: Autozygosity islands and ROH patterns in Nellore lineages: evidence of selection for functionally important traits
Source: BMC Genomics. 2018 Sep 17;19:680. doi: 10.1186/s12864-018-5060-8 (PMC6142381; doi:10.1186/s12864-018-5060-8)

Number of times a SNP appeared in a ROH

8

7

6

5

Log<sub>10</sub>-transformed

1

2

3

4

5

6

7

8

9

10

11

12

13

14

15

16

17

18

19

20

21

22

23

24

25

26

27

28

Chromosome

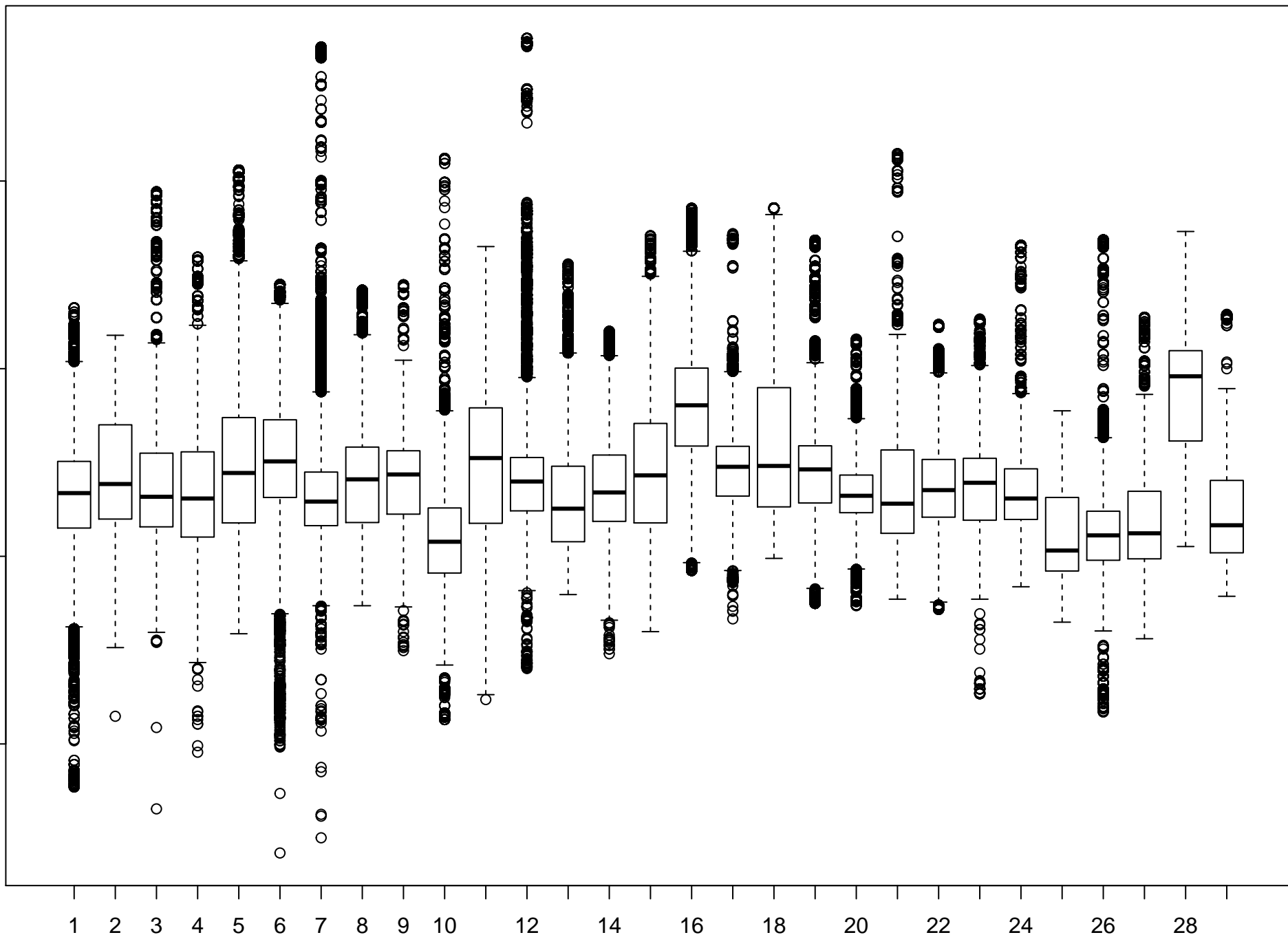

Supplement: Supplementary file 9 — Outliers SNPs for the genotyped animals (n = 9386) according to Boxplot distribution. (PDF 227 kb) [file 12864_2018_5060_MOESM9_ESM.pdf]
