# Supplementary material for: Autozygosity islands and ROH patterns in Nellore lineages: evidence of selection for functionally important traits
Source: BMC Genomics. 2018 Sep 17;19:680. doi: 10.1186/s12864-018-5060-8 (PMC6142381; doi:10.1186/s12864-018-5060-8)
Supplement: Supplementary file 10 — Outliers SNPs for each Nellore lineage (n = 8646) according to Boxplot distribution. (PDF 340 kb) [file 12864_2018_5060_MOESM10_ESM.pdf]

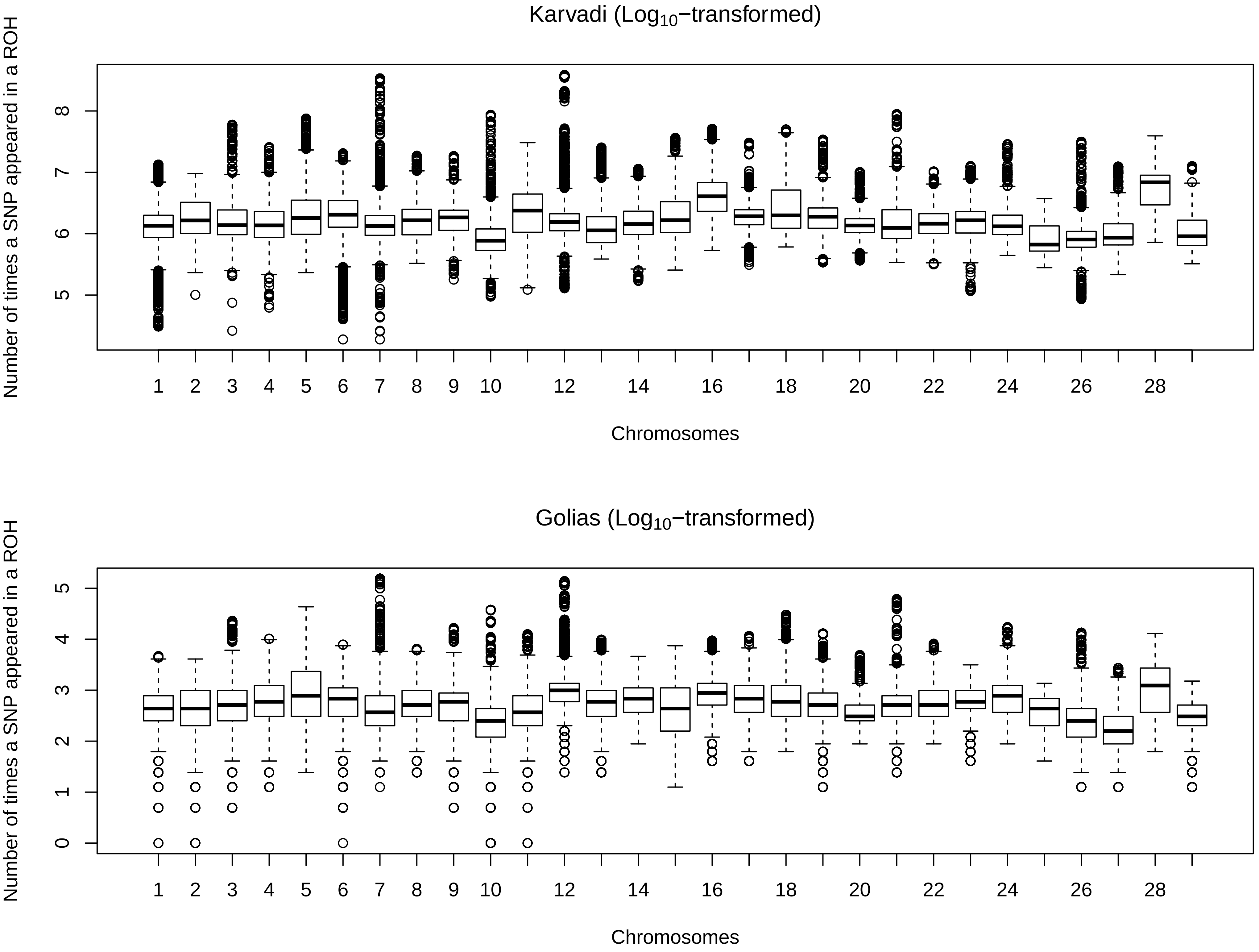

Karvadi (Log$_{10}$−transformed)
Number of times a SNP appeared in a ROH
Chromosomes
Golias (Log$_{10}$−transformed)
Number of times a SNP appeared in a ROH
Chromosomes

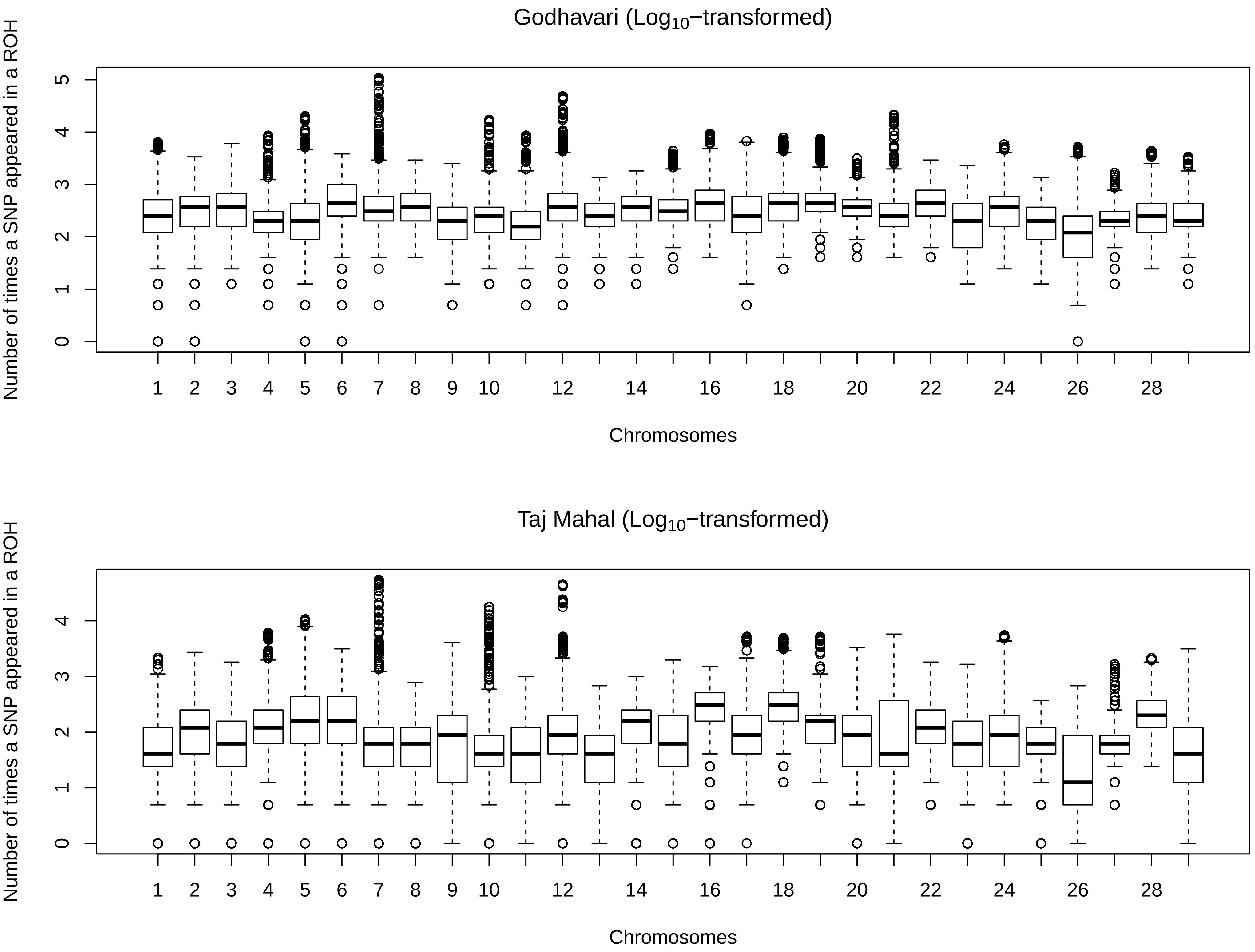
Godhavari (Log10−transformed)
Number of times a SNP appeared in a ROH
0
1
2
3
4
5
1 2 3 4 5 6 7 8 9 10 12 14 16 18 20 22 24 26 28
Chromosomes
Taj Mahal (Log10−transformed)
Number of times a SNP appeared in a ROH
0
1
2
3
4
1 2 3 4 5 6 7 8 9 10 12 14 16 18 20 22 24 26 28
Chromosomes

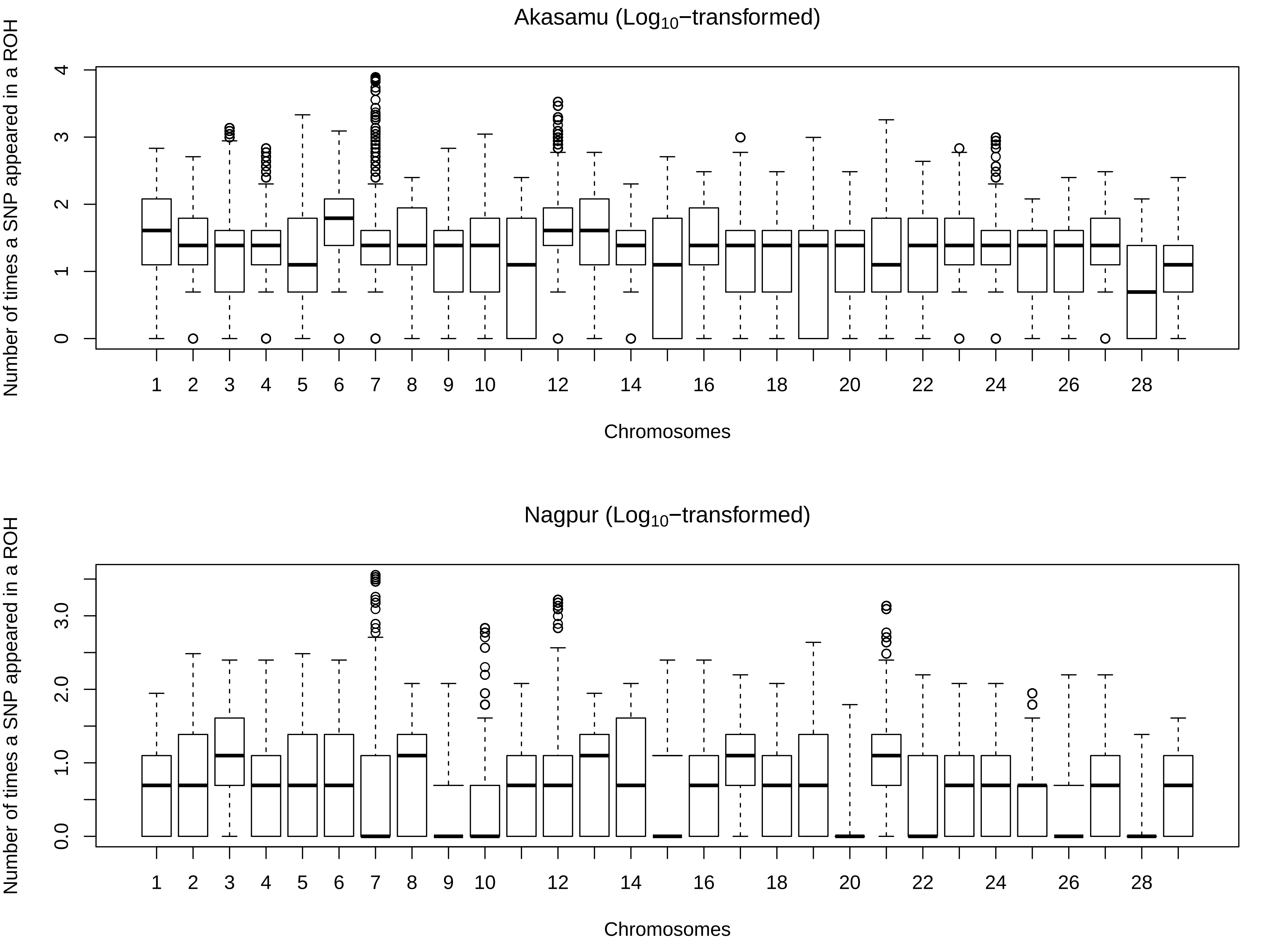

Akasamu (Log$_{10}$−transformed)
Number of times a SNP appeared in a ROH
0
1
2
3
4
1 2 3 4 5 6 7 8 9 10 12 14 16 18 20 22 24 26 28
Chromosomes
Nagpur (Log$_{10}$−transformed)
Number of times a SNP appeared in a ROH
0.0
1.0
2.0
3.0
1 2 3 4 5 6 7 8 9 10 12 14 16 18 20 22 24 26 28
Chromosomes
